# SARS-CoV-2 mutant spectra as variant of concern nurseries: endless variation?

**DOI:** 10.3389/fmicb.2024.1358258

**Published:** 2024-03-14

**Authors:** Brenda Martínez-González, María Eugenia Soria, Pablo Mínguez, Ramón Lorenzo-Redondo, Llanos Salar-Vidal, Alberto López-García, Mario Esteban-Muñoz, Antoni Durán-Pastor, Pilar Somovilla, Carlos García-Crespo, Ana Isabel de Ávila, Jordi Gómez, Jaime Esteban, Ricardo Fernández-Roblas, Ignacio Gadea, Esteban Domingo, Celia Perales

**Affiliations:** ^1^Department of Molecular and Cell Biology, Centro Nacional de Biotecnología (CNB-CSIC), Consejo Superior de Investigaciones Científicas (CSIC), Campus de Cantoblanco, Madrid, Spain; ^2^Department of Clinical Microbiology, Instituto de Investigación Sanitaria-Fundación Jiménez Díaz University Hospital, Universidad Autónoma de Madrid (IIS-FJD, UAM), Madrid, Spain; ^3^Centro de Biología Molecular “Severo Ochoa” (CSIC-UAM), Campus de Cantoblanco, Madrid, Spain; ^4^Department of Genetics and Genomics, Instituto de Investigación Sanitaria-Fundación Jiménez Díaz University Hospital, Universidad Autónoma de Madrid (IIS-FJD, UAM), Madrid, Spain; ^5^Centre for Biomedical Network Research on Rare Diseases (CIBERER), Instituto de Salud Carlos III, Madrid, Spain; ^6^Bioinformatics Unit, Instituto de Investigación Sanitaria-Fundación Jiménez Díaz University Hospital, Universidad Autónoma de Madrid (IIS-FJD, UAM), Madrid, Spain; ^7^Division of Infectious Diseases, Center for Pathogen Genomics and Microbial Evolution, Feinberg School of Medicine, Northwestern University, Chicago, IL, United States; ^8^Centre for Biomedical Network Research on Infectious Diseases (CIBERINFEC), Madrid, Spain; ^9^Health Research Institute IIS-FJD, Fundación Jiménez Diaz University Hospital, Madrid, Spain; ^10^Departamento de Biología Molecular, Universidad Autónoma de Madrid, Campus de Cantoblanco, Madrid, Spain; ^11^Instituto de Parasitología y Biomedicina “López-Neyra” (CSIC), Parque Tecnológico Ciencias de la Salud, Granada, Spain

**Keywords:** COVID-19, viral quasispecies, ultra-deep sequencing, viral emergence, clade-discordant residues, variant of concern

## Abstract

**Introduction:**

SARS-CoV-2 isolates of a given clade may contain low frequency genomes that encode amino acids or deletions which are typical of a different clade.

**Methods:**

Here we use high resolution ultra-deep sequencing to analyze SARS-CoV-2 mutant spectra.

**Results:**

In 6 out of 11 SARS-CoV-2 isolates from COVID-19 patients, the mutant spectrum of the spike (S)-coding region included two or more amino acids or deletions, that correspond to discordant viral clades. A similar observation is reported for laboratory populations of SARS-CoV-2 USA-WA1/2020, following a cell culture infection in the presence of remdesivir, ribavirin or their combinations. Moreover, some of the clade-discordant genome residues are found in the same haplotype within an amplicon.

**Discussion:**

We evaluate possible interpretations of these findings, and reviewed precedents for rapid selection of genomes with multiple mutations in RNA viruses. These considerations suggest that intra-host evolution may be sufficient to generate minority sequences which are closely related to sequences typical of other clades. The results provide a model for the origin of variants of concern during epidemic spread─in particular Omicron lineages─that does not require prolonged infection, involvement of immunocompromised individuals, or participation of intermediate, non-human hosts.

## Introduction

Mutant spectra of RNA viral quasispecies are repositories of genomes whose individual behavior may differ from that displayed by the complete population where they reside. Documented examples of a difference between the progeny of an individual genome and the ensemble which contains that genome include resistance to antibodies or antiviral agents, altered cell tropism or virulence, or capacity to induce interferon (or respond to it), among others (reviewed in the study by Domingo et al., 2021b). SARS-CoV-2 isolates have evidenced yet another feature of their mutant spectra, consisting in the presence of low-frequency amino acids (using as reference the consensus sequence of the corresponding isolate), which are typical of a SARS-CoV-2 clade that differs from the clade assigned to the isolate (Sun et al., 2021; Martínez-González et al., 2022a,b). This has been established with virus from independent COVID-19 patient cohorts and vaccine breakthrough COVID-19 cases (Martínez-González et al., 2022c).

The question we address in the present study is whether the intra-host variation of SARS-CoV-2 is sufficiently frequent and extensive to be able to generate in mutant spectra sequences which are atypical of the clade of the isolate. The question is relevant to the origin of variants of interest (VOIs) and variants of concern (VOCs),^1^ for which several models have been proposed (Bashor et al., 2021; Harari et al., 2022; Mallapaty, 2022; Chaguza et al., 2023; Riddell and Cutino-Moguel, 2023). Some of these models assume that replicative or environmental singularities (i.e., viral replication in immunocompromised individuals, chronicity of the infection, or participation of intermediary non-human hosts) may be required to attain a number of SARS-CoV-2 genome modifications that is necessary to produce an epidemiologically fit virus whose progeny qualifies as a new viral clade.

In an attempt to clarify this question, we have analyzed the SARS-CoV-2 mutant spectra of the spike (S) genomic region in search of clade-discordant residues. The analysis has been performed with diagnostic samples of 11 COVID-19 cases from a patient cohort in Madrid (Spain) and 11 laboratory populations derived from isolate USA-WA1/2020. A more stringent criterion has been the presence of combinations of two or more amino acids or deletions that correspond to a different clade from the one defined by the consensus sequence of the isolate. The objective is to explore the presence in mutant spectra of multiple genomic changes that may guide the population through mutational pathways toward clade modification. Cases involving single amino acid changes or deletions have been previously documented with other SARS-CoV-2 isolates (Sun et al., 2021; Martínez-González et al., 2022a,b,c).

The study has been carried out with six amplicons of the S-coding region, which covers encoded amino acid 7,062 of nsp16 (ORF1b) to amino acid 694 of S; some of the amino acids in the consensus sequence of the regions analyzed have been utilized for clade identification.^2^ A high resolution, ultra-deep sequencing that reaches a cutoff value of 0.1% for point mutations and deletions has been used. The progeny of a single infection of Vero E6 cells with SARS-CoV-2 USA-WA1/2020 in the absence or presence of remdesivir (Rdv) and/or ribavirin (Rib) has been also analyzed with the same methodology and objective.

We document that the mutant spectrum of the virus in the diagnostic sample of 6 out of 11 COVID-19 patients included two or more amino acids and deletions that are diagnostic of a clade different from that assigned to the isolate. In several amplicon patients, this diagnostic criterion was met with amino acids and deletions present in one haplotype. Clade-discordant residues have been identified also in the mutant spectrum of laboratory populations of SARS-CoV-2 USA-WA1/2020, following a cell culture infection in the presence of Rdv and Rib but not in parallel infections in absence of the inhibitors. From the evaluation of possible factors that may contribute to the presence of such outlier sequences in mutant spectra, we suggest that such sequences arise during intra-host virus multiplication, and that, upon further mutation and recombination (Jackson et al., 2021; Sekizuka et al., 2022), they can originate genomes that later define new viral clades.

## Materials and methods

### SARS-CoV-2 patient samples, laboratory populations, and infections

The origin of the nasopharyngeal swab samples from COVID-19 patients and the clinical profile of the patients used in the present study are shown in Table 1. The 11 patient samples were collected at the University Hospital Fundación Jiménez Díaz (Madrid, Spain); the patients were diagnosed as positive for SARS-CoV-2 using a specific RT-PCR analysis, and the viral load (Ct) was measured by the specific real-time PCR VIASURE; these procedures have been previously described for other isolates of the Madrid cohort (Soria et al., 2021). None of the patients were qualified as immunocompromised, and none were treated with viral polymerase inhibitors.

**Table 1:** Demographic data of patients and classification of the consensus sequence of the samples analyzed.

Patient ID^a^	Ct average^b^	Date of collection	Clade^c^	Lineage^c^	Variant^c^
Pt454	18.5	20/01/2021	20I	B.1.1.7	Alpha
Pt455	13.5	12/02/2021	20I	B.1.1.7	Alpha
Pt456	9.5	11/02/2021	20I	B.1.1.7	Alpha
Pt457	13	13/02/2021	20I	B.1.1.7	Alpha
Pt458	20	15/01/2021	20I	B.1.1.7	Alpha
Pt459	27	02/02/2021	20E	B.1.177	20E (EU1)/B.1.177
Pt460	16.7	01/02/2021	20E	B.1.177	20E (EU1)/B.1.177
Pt461	22	02/02/2021	20C	B.1.575.1	20C/B.1.575.1
Pt462	17.3	02/02/2021	20E	B.1.177	20E (EU1)/B.1.177
Pt463	13.7	30/01/2021	20A	B.1.160	20A/B.1.160
Pt500	24	10/08/2021	20H	B.1.351.5	Beta

a In the patient cohort there were 7 females and 4 males; 9 patients were non-smokers, 1 of them was a smoker, and we do not have information for other patients. None of the patients were diabetic except one. The average age was 52 years.

b The average of Ct (cycle threshold, which is inversely correlated with viral RNA level) has been calculated based on the Ct data for ORF1b, S and N genes.

c Classification according to the consensus sequence of these isolates analyzed by COVIDSeq (Illumina).

The laboratory populations analyzed resulted from infection of Vero E6 by SARS-CoV-2 USA-WA1/2020 (NCBI reference sequence NR-52281_70036318) (clade 19B) at a multiplicity of infection of 0.001 PFU/cell (monolayers of 2 × 10^5^–1 × 10^6^ Vero E6 cells) as part of our ongoing project on lethal mutagenesis of SARS-CoV-2. The infections were carried out either in absence of drugs or in the presence of Rdv (5 μM or 10 μM) alone, Rib (100 μM or 150 μM) alone, or combinations of the two drugs (Rdv 2.5 μM or 5 μM; Rib 80 μM or 100 μM); the duration of the infection was 48 h. Drug concentrations were chosen according to their CC_50_ and EC_50_ values, to allow the presence of sufficient viral RNA for ultra-deep sequencing analysis (Somovilla et al., 2023; García-Crespo et al., 2024). All passage series in the absence and presence of drugs and all titrations for virus quantifications were carried out in triplicate (García-Crespo et al., 2024).

### Consensus sequencing by COVIDSeq

SARS-CoV-2 RNA was extracted from 140 μL medium from a nasopharyngeal swab sample or the laboratory samples using the QIAamp Viral RNA Mini Kit 250 (QIAGEN), according to the manufacturer’s instructions. Then, libraries were prepared using the Illumina COVIDSeq protocol (Illumina). The SARS-CoV-2 genome was amplified using COVIDSeq Primer Pool-1 and 2 provided by Illumina, which was designed to amplify the entire genome. PCR amplicons were fragmented and tagmented with adapter sequences before being amplified. Adaptor-ligated amplicons were amplified using IDT for Illumina PCR Indexes Set 1. The individual library was quantified using Qubit 2.0 fluorometer (Invitrogen, Inc) and pooled in equimolar concentration as recommended by Illumina. The library was prepared using Illumina COVIDSeq Assay Box 1 – 96 samples, and library products were sequenced using the Illumina MiSeq sequencing platform with MiSeq v2 Reagent Kit 300 cycles PE.

FASTQ data from MiSeq were analyzed using the Basespace (Illumina) for quality check (QC), FASTA generation, genome assembly, and identification of SARS-CoV-2 variants.^3^ DRAGEN COVID Lineage (Version: 3.5.4) was used for variant determination (NextClade v2.14.1) and lineage assignment [Phylogenetic Assignment of Named Global Outbreak Lineages (PANGOLIN)]. All sequences ranked as “good” according to the QC overall score. The consensus genomic sequences allowed the assignment of each isolate to a specific clade (indicated in the relevant tables and figures of the Results section) using the Nextstrain tools Nextclade^4^ and CoVariants.^5^

### Amplicon-based ultra-deep sequencing

The oligonucleotide primers used for amplification of viral RNA from the patients covered the end of ORF1b and the S-coding region (nucleotides 21,448–23,645; numbering according to the genome of Wuhan-Hu-1 NCBI sequence NC_045512.2). Primers were designed based on 663 SARS-CoV-2 sequences from the SARS-CoV-2 database of NCBI, which were aligned with the NC_045512.2 sequence (Supplementary Table S1).

SARS-CoV-2 RNA was extracted using the QIAamp Viral RNA Mini Kit 250 (QIAGEN). Amplification was performed using 5 μL of RNA mixed with 2 μL forward and 2 μL reverse primers at 50 ng/μl, 10 μL 5x buffer, and 1 μL polymerase for each amplicon with the Transcriptor One-Step RT-PCR Kit (Roche Applied Science). As negative control, amplification in the absence of RNA was carried out in parallel for each amplicon. Amplification was carried out with RNA samples diluted 1/10, 1/100, and 1/1,000; only when dilution 1/1,000 produced a visible DNA band, the sequence analysis was carried out using the undiluted sample. The amplification products were analyzed by 2% agarose gel electrophoresis, including the Gene Ruler 1 Kb Plus DNA Ladder (Thermo Fisher Scientific) as the molar mass standard. PCR products were purified using the QIAquick Gel Extraction Kit (QIAGEN), quantified using the Qubit dsDNA Assay Kit (Thermo Fisher Scientific), and tested for quality (TapeStation System) prior to nucleotide sequencing.

PCR products were adjusted to a concentration of 4 × 10^9^ molecules/μl and were purified using KAPA Pure Beads (Kapa biosystems, Roche) to obtain the DNA pool; the latter was quantified using Qubit and then adjusted to 1.5 ng/μl. The pool was indexed using the SeqCap Adapter Kit A/B (Nimblegen; 24 Index) during the library preparation with KAPA HyperPrep Kit (Roche). The final DNA pool was quantified and sequenced using the Illumina MiSeq sequencing platform with the MiSeq Reagent kit version 3 (2 × 300 bp mode with the 600-cycle kit).

For the bioinformatics analysis, the fastq files derived from the amplicon-based ultra-deep sequencing were analyzed with the SeekDeep pipeline (Hathaway et al., 2018) using the following options: --extraExtractorCmds = -- checkRevComplementForPrimers –primerNumOfMismatches 3″ “—extraProcessClusterCmds = --fracCutOff 0.001 –rescueExcludedOneOffLowFreqHaplotypes.”

### Deep sequencing reliability and precautions during handling of SARS-CoV-2 samples

Reliability of the mutations and haplotypes identified with a point mutation and deletion frequency cutoff of 0.1% is based on the average clean read coverage of 97,437 (range 32,390–253,780) attained in the present study (Supplementary Table S2). Furthermore, 92.3% of the residues that were sequenced yielded a quality score of Q > 30, according to Illumina specifications.^6^

In addition, we previously described the following arguments for reliability of the 0.1% percentage sequencing cutoff: (i) A total of 96 out of 97 mutations and all the 10 deletions that were recorded in the mutant spectrum of virus from COVID-19 patients with a 0.5% mutation frequency cutoff were also identified with the 0.1% cutoff. (ii) The mutation type bias and the distribution of mutations among the first, second, and third codon positions of the nucleotide triplets were the same when setting the mutation frequency cutoff at 0.5 and 0.1%. (iii) There is a similar percentage of amino acid substitutions that are represented in the GISAID data bank, with the two cutoff values. (iv) There is a similar average acceptability and predicted functional effects (using the PAM250 substitution matrix and the SNAP2 predictor, respectively) of the amino acid substitutions deduced from the ultra-deep sequencing data, with the two resolution values. (v) Upon lowering the point mutation frequency cutoff from 0.5 to 0.1%, the total number of different mutations counted separately for three COVID-19 patient groups (that exhibited mild, moderate, and severe COVID-19, with the criteria described in the study by Martínez-González et al., 2022b) increased 55-fold for the nsp12 (polymerase)-coding region and 97-fold for the S-coding region. None of the values, agreements, and differences listed in points (i) to (v) would be expected if the mutations recorded with the 0.1% frequency cutoff were the result of sequencing mistakes (see Martínez-González et al., 2022a; Domingo et al., 2023; Delgado et al., 2024 for additional details on these arguments).

Regarding procedures and controls to avoid cross-contamination during laboratory handling of the samples or the extracted RNA, the following actions are established in our laboratory. Care is taken to avoid cross-contaminations at the time of sample collection and transport to the BSL-3 facility at Centro de Biología Molecular Severo Ochoa (CSIC-UAM). In the BSL-3 laboratory, each sample is handled separately using high containment Class II, type A biological safety cabins that comply with European regulation EN-12469. Furthermore, an experiment to evaluate the likelihood of within-cabin cross-contamination was performed as follows: one open tube containing water was maintained during the several successive extractions of nasopharyngeal swabs. Upon completion of these manipulations, the contents of the open tube were subjected to the sequential manipulations for RT-PCR amplification; no DNA band was obtained. Non-recyclable, non-disposable laboratory materials (including micro-pipette bodies) were disinfected with VIRKON (Rely+On^™^ Virkon^™^ LANXESS) solution, following the handling of a sample. Preparation of reagents for RT-PCR amplifications was carried in an isolation cabin located in the standard biochemistry laboratory (where no SARS-CoV-2 samples are stored). All cabins were maintained under UV irradiation when not in use. In addition, a negative RT-PCR amplification control consisting of a sample with all the reagents, except for RNA, was included for each amplicon and amplification assay. All experiments were conducted with low binding barrier sterile tips (Corning) that prevent aerosol contamination. No evidence of contamination with undesired templates was obtained in any of the control assays. Furthermore, following the established protocol, during library preparation for ultra-deep sequencing, the amplicons of each sample were indexed to identify the set of sequences for each sample.

For all these considerations regarding reliability of mutation and deletion identification using a 0.1% cutoff value, our workflow, the precautions taken for sample collection and transport, biosafety measures, and the experimental controls (either performed or included in sample processing), laboratory contamination to account for the presence of outlier haplotypes in SARS-CoV-2 mutant spectra is exceedingly unlikely (see Discussion for additional arguments).

## Results

### Mutant spectra of SARS-CoV-2 from COVID-19 patients with multiple amino acids or deletions typical of a different viral clade

**Figure 1:**
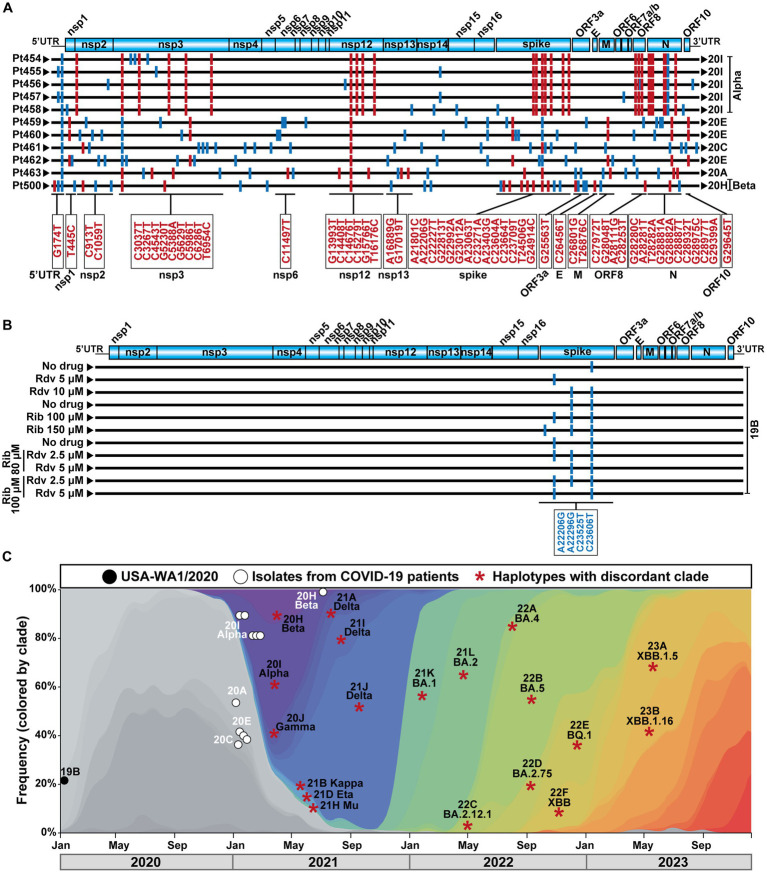
SARS-CoV-2 genomic sequences, clade identification, and epidemiological context. **(A)** Representation of the SARS-CoV-2 genome, with encoded proteins (top), followed by the consensus genomic sequence of the isolates from the 11 patients analyzed in the present work. Sequences were obtained by COVIDSeq. The patient code is given on the left, and the clade assigned to each isolate is written on the right. Mutations relative to the sequence of the Wuhan-Hu-1 genome (NCBI sequence NC_045512.2) are indicated as short vertical lines; mutations that served for clade identification are in red color, and their location in the genome is depicted in the boxes below the alignment. Other mutations are depicted in blue. Several mutations used for clade identification are also present in virus that belong to a different clade; these mutations are drawn as blue lines. All mutations (and corresponding amino acid substitutions) and deletions are listed in Supplementary Table S3. **(B)** Schematic representation of the consensus genomic sequence of the laboratory populations derived from SARS-CoV-2 USA-WA1/2020 analyzed in the present study. The absence or presence of drug (Rdv, remdesivir; Rib, ribavirin) and its concentration is indicated on the left. Mutations relative to the Wuhan-Hu-1 genome (NCBI sequence NC_045512.2) are indicated as short vertical blue lines; their position in the genome is given in the box below the alignment. Mutations A22206G and C23525T have been used to define VOCs (https://clades.nextstrain.org). Mutations relative to the Wuhan-Hu-1 isolate that were present in the parental USA-WA1/2020 are not included. **(C)** Temporal position of the SARS-CoV-2 analyzed in the present study, and of the clade-discordant lineages identified in their mutant spectrum, that compiled with the inclusion criteria. Symbol code is given in the upper box, and epidemiological time is depicted at the bottom. Each asterisk identifies a discordant clade and lineage, that may be represent several times in our analysis (see text).

The COVID-19 diagnostic samples of Pt454 to Pt463 were collected during January and February 2021, and the sample of Pt500 was collected in August 2021 (Table 1). The 11 isolates were assigned to clades 20A, 20C, 20E, 20H, or 20I. Their genomic consensus nucleotide sequence exhibited multiple mutations relative to the reference Wuhan-Hu-1 sequence (NCBI NC_045512.2) (mutations are presented schematically in Figure 1A and shown in Supplementary Table S3). Some mutations are those that serve for clade assignment, a few mutations mark the diversification relative to the Wuhan-Hu-1 isolate, and the majority of additional mutations were scattered along the genome. No mutations were located within the nsp9-nsp10-nsp11-coding region. Temporal clade position along the COVID-19 pandemics is shown in Figure 1C.

**Figure 2:**
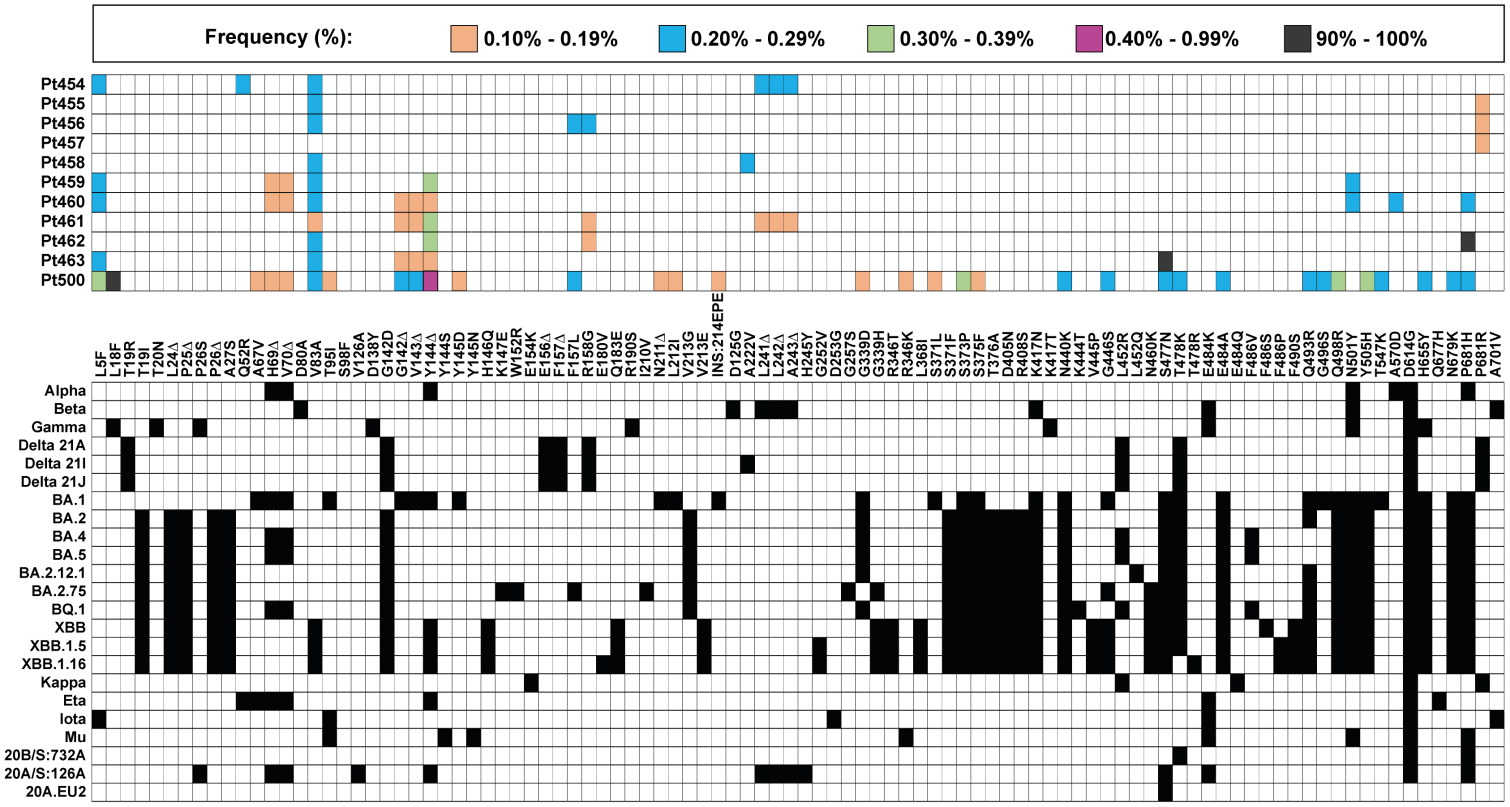
Heat map of all clade-discordant amino acid substitutions and deletions found in the S-coding region of virus from the 11 patients analyzed in the present study. The top box gives the color code for the frequency of each substitution or deletion (Δ) (indicated between the two grid panels); INS:214EPE means an insertion of amino acids EPE in position 214. Patient code is given on the left of the top panel. The bottom grid panel depicts as filled squares the substitutions used to define lineages and sub-lineages (according to https://clades.nextstrain.org) (region covered by A1–A6 amplicons of S-coding region). The subset of clade discordant amino acids that meet the inclusion criteria detailed in the text are listed in Table 2.

The mutant spectrum of the S-coding region of the 11 isolates was analyzed using the Illumina MiSeq sequencing platform, in search of amino acids in the S protein and of deletions in the S-coding region, whose presence did not match the corresponding residues of the clade assigned to the isolate. A heat map overview shows that the majority of clade-discordant amino acids and deletions were found at frequencies in the range of 0.10–0.39% (Figure 2).

A more stringent criterion to accept a patient’s virus as positive for clade-discordant residues was established. It consisted in that the mutant spectrum of the virus from a patient should contain at least two genome features (either amino acid or deletions) that were previously assigned to a clade which is different from the clade assigned to the isolate. The discrepant amino acid and deletion should be the result of independent genome modifications (that is, an amino acid change should not be a consequence of a deletion). The search criterion was fulfilled for SARS-CoV-2 of 6 out of the 11 patients analyzed. Clade-discordant amino acids in S or deletions in the S-coding region belonged mainly to Omicron lineages (87.5% of the total), but some were found that corresponded to Alpha, Beta, or Delta lineages (Table 2) (the complete list of haplotypes for each sample is shown in Supplementary Tables S4–S14). The search criterion was met even for 9 out of 32 different haplotypes (highlighted in Table 2); they were identified in virus from 3 patients, notably in patient Pt500, with 6 out of the 10 haplotypes including clade-discordant residues.

**Table 2:** Amino acids in the spike SARS-CoV-2 mutant spectra that are typical of a different viral clade^a^.

Patient^b^	Lineage (OMS)/clade^c^	Spike amplicon^d^	Haplotype number (frequency %)^e^	Discordant amino acid or deletion^f^	Discordant lineage/clade^g^	Outbreak.info presence (%)^h^
Pt456	B.1.1.7 (Alpha)/20I	A2	23 (0.22)	R158G	Delta (B.1.617.2/21A, 21I, 21 J)	27/1.157.771 (0.002%)
		A6	64 (0.18)	P681R	Delta (B.1.617.2/21A, 21I, 21 J); Kappa (B.1.617.1/21B)	299/1.157.771 (0.026%)
Pt459	B.1.177/20E	A2	29 (0.20)	V83A	Omicron (XBB/22F; XBB.1.5/23A; XBB.1.16/23B)	2/78.042 (0.002%)
			*33 (0.16)	21,765–21,770 (H69∆, V70∆) + 21,992–21,994 (Y144∆)	Alpha (B.1.1.7/20I); Omicron (BA.1/21 K); Eta (B.1.515)/21D; S:126A/20A	0/78.042 (0%)
			26 (0.19)	21,992–21,994 (Y144∆)	Alpha (B.1.1.7/20I); Omicron (BA.1/21 K; XBB/22F; XBB.1.5/23A; XBB.1.16/23B); Eta (B.1.515/21D); S:126A/20A	0/78.042 (0%)
		A5	3 (0.28)	N501Y	Alpha (B.1.1.7/20I); Omicron (BA.1/21 K; BA.2/21 L; BA.4/22A; BA.5/22B; BA.2.12.1/22C; BA.2.75/22D; BQ.1/22E; XBB/22F; XBB.1.5/23A; XBB.1.16/23B); Mu (B.1.621/21H)	31/78.042 (0.040%)
Pt460	B.1.177/20E	A1	11 (0.22)	21,765–21,770 (H69∆, V70∆)	Alpha (B.1.1.7/20I); Omicron (BA.1/21 K; BA.4/22A; BA.5/22B; BQ.1/22E); Eta(B.1.525/21D); S:126A/20A	67/78.042 (0.089%)
		A2	14 (0.23)	V83A	Omicron (XBB/22F; XBB.1.5/23A; XBB.1.16/23B)	2/78.042 (0.002%)
			*34 (0.11)	21,765–21,770 (H69∆, V70∆) + 21,992–21,994 (Y144∆)	Alpha (B.1.1.7/20I); Omicron (BA.1/21 K); Eta (B.1.525/21D); S:126A/20A	0/78.042 (0%)
			33 (0.11)	21,992–21,994 (Y144∆)	Alpha (B.1.1.7/20I); Omicron (BA.1/21 K; XBB/22F; XBB.1.5/23A; XBB.1.16/23B); Eta (B.1.525/21D); S:126A/20A	0/78.042 (0%)
			35 (0.11)	21,980–21,990 (F140∆, L141∆, G142∆; V143∆)	Omicron (BA.1/21 K)	0/78.042 (0%)
		A5	4 (0.29)	N501Y	Alpha (B.1.1.7/20I); Omicron (BA.1/21 K; BA.2/21 L; BA.4/22A; BA.5/22B; BA.2.12.1/22C; BA.2.75/22D; BQ.1/22E; XBB/22F; XBB.1.5/23A; XBB.1.16/23B); Mu (B.1.621/21H)	31/78.042 (0.040%)
		A6	*11 (0.21)	A570D + P681H	Alpha (B.1.1.7/20I)	4/78.042 (0.005%)
Pt461	B.1.575.1/20C	A2	29 (0.22)	V83A	Omicron (XBB/22F; XBB.1.5/23A; XBB.1.16/23B)	0/3.567 (0%)
			2 (0.36)	21,992–21,993 (Y144∆)	Alpha (B.1.1.7/20I); Omicron (BA.1/21 K; XBB/22F; XBB.1.5/23A; XBB.1.16/23B); Eta (B.1.525/21D); S:126A/20A	0/3.567 (0%)
Pt462	B.1.177/20E	A2	62 (0.18)	V83A	Omicron (XBB/22F; XBB.1.5/23A; XBB.1.16/23B)	2/78.042 (0.002%)
			11 (0.25)	21,980–21,990 (F140∆, L141∆, G142∆; V143∆)	Omicron (BA.1/21 K)	0/78.042 (0%)
			45 (0.19)	21,983–21,991 (L141∆, G142∆; V143∆)	Omicron (BA.1/21 K)	0/78.042 (0%)
			4 (0.30)	21,992–21,993 (Y144∆)	Alpha (B.1.1.7/20I); Omicron (BA.1/21 K; XBB/22F; XBB.1.5/23A; XBB.1.16/23B); Eta (B.1.525/21D); S:126A/20A	0/78.042 (0%)
		A3	41 (0.11)	22,283–22,291 (L241∆, L242∆, A243∆)	Beta (B.1.351/20H); S:126A/20A	0/78.042 (0%)
Pt500	B.1.351 (Beta)/20H	A1	*7 (0.16)	A67V + ∆21,765–21,770 (H69∆, V70∆)	Omicron (BA.1/21 K); Eta (B.1.525/21D)	0/36.239 (0%)
		A2	*40 (0.12)	A67V + T95I + ∆21,765–21,770 (H69∆, V70∆) + ∆21,987–21,995 (G142∆, V143∆, Y144∆) + Y145D^i^	Omicron (BA.1/21 K)	0/36.239 (0%)
			19 (0.23)	V83A	Omicron (XBB/22F; XBB.1.5/23A; XBB.1.16/23B)	0/36.239 (0%)
			30 (0.20)	F157L	Omicron (BA.2.75/22D)	15/36.239 (0.041%)			
1 (0.42)	∆21,992–21,993 (Y144∆)	Alpha (B.1.1.7/20I); Omicron (BA.1/21 K; XBB/22F; XBB.1.5/23A; XBB.1.16/23B); Eta (B.1.525/21D); S:126A/20A	0/36.239 (0%)						
39 (0.13)	∆21,983–21,994 (L141∆, G142∆, V143∆, Y144∆)	Omicron (BA.1/21 K)	0/36.239 (0%)					
A3	*30 (0.16)	L212I + ∆22,194–22,196 (N211∆) + INS:214EPE	Omicron (BA.1/21 K)	0/36.239 (0%)				
A4	*26 (0.16)	G339D + S371L + S373P + S375F	Omicron (BA.1/21 K)	1/36.239 (0.003%)				
	15 (0.21)	S373P	Omicron (BA.1/21 K; BA.2/21 L; BA.4/22A; BA.5/22B; BA.2.12.1/22C; BA.2.75/22D; BQ.1/22E; XBB/22F; XBB.1.5/23A; XBB.1.16/23B)	5/36.239 (0.014%)				
A5	*14 (0.22)	N440K + G446S + S477N + T478K + E484A + Q493R + G496S + Q498R + N501Y + Y505H + T547K	Omicron (BA.1/21 K)	0/36.239 (0%)				
	36 (0.12)	Y505H	Omicron (BA.1/21 K; BA.2/21 L; BA.4/22A; BA.5/22B; BA.2.12.1/22C; BA.2.75/22D; BQ.1/22E; XBB/22F; XBB.1.5/23A; XBB.1.16/23B)	1/36.239 (0.003%)				
A6	5 (0.27)	H655Y + N679K + P681H	Omicron (BA.1/21 K; BA.2/21 L; BA.4/22A; BA.5/22B; BA.2.12.1/22C; BA.2.75/22D; BQ.1/22E; XBB/22F; XBB.1.5/23A; XBB.1.16/23B)	0/36.239 (0%)

a Only cases with two or more discordant amino acids variations [mutations, deletions (∆) and/or insertions (INS)] in the same or different amplicons are tabulated. The complete list of haplotypes in the six amplicons of the S-coding region for each sample is given in Supplementary Tables S4–S14.

b Patient clinical profile, diagnostic sample collection date, and amount of viral RNA (Ct value) are compiled in Table 1.

c Clade and lineage are assigned according to Nextstrain (https://nextstrain.org/ncov/gisaid/global/). Those lineages with a Greek letter designed by WHO are indicated.

d The S amplicons covered the following residues: A1: nucleotides 21,448–21,841; A2: nucleotides 21,727–21,217; A3: nucleotides 22,111–22,515; A4: nucleotides 22,487–22,882; A5: nucleotides 22,827–23,268; A6: nucleotides 23,259–23,645. These six amplicons spanned amino acids residue 7,062 of nsp16 (ORF1b) to 694 of S.

e Asterisks indicate haplotypes that included two clade-discordant variations [mutations, deletions (∆) and/or insertions (INS)]. The sequence of the haplotypes identified in the present analysis is given in Supplementary Tables S4–S14. The frequency of the haplotype has been calculated as percentage of all clean reads, given by the ultra-deep sequencing procedure that attained a 0.1% mutation frequency cut-off (see Materials and Methods).

f Amino acids that are not represented in the consensus sequence of the isolate (clade/lineage as explained in footnote c), and that are present in the clades/lineages indicated in the next column (footnote g). Underlined amino acids are not characteristic of the variant indicated in the next column (footnote g).

g The discordant clades/lineages in which their consensus sequence includes the amino acid indicated in the previous column (footnote f). Those lineages with a Greek letter designed by WHO are indicated.

h Number of sequences with the corresponding clade-discordant residues that are found in the indicated number of consensus sequences of this clade deposited in database outbreak.info (https://outbreak.info/); the percentage is given in parenthesis.

i Substitution Y145D is caused by the deletion ∆21,987–21,995.

Thus, the mutant spectrum of the S-coding region of SARS-CoV-2 isolates of a given clade may contain multiple clade-discordant amino acids and deletions. Some of them are present in the same haplotype.

### Discordant amino acids and deletions in laboratory preparations of SARS-CoV-2

Clade-discordant amino acids and deletions found in the diagnostic samples might have arisen in the course of the intra-host evolution of the virus of the patient we have analyzed, despite the patient having been infected by a clade-defined SARS-CoV-2. Once generated, genomes with discordant residues might have remained at a frequency range of 0.11–0.42% for individual haplotypes (Table 2). This frequency range might be influenced by the fitness balance of the newly arising versus the resident swarm or by suppressive effects exerted by the population ensemble, despite the new genomes having high fitness (de la Torre and Holland, 1990; Domingo et al., 2023). As an alternative to this within-patient origin, the genomes with clade-discordant residues might have been present already in the infecting virus, either because they arose in (and were transmitted from) the infecting contact or there was a low level, general circulation of variants that co-infected patients but remained epidemiologically undetected (Gregory et al., 2022).

A possibility to gain insight into the origin of clade-discordant residues was provided by laboratory populations derived from the initial USA-WA1/2020 isolate that belongs to clade 19B (Harcourt et al., 2020). With this isolate, we have recently documented the synergistic antiviral activity exerted by the nucleoside analogs Rdv and Rib in cell culture (García-Crespo et al., 2024). Rib and, to a lesser extent, Rdv increased the mutation frequency of SARS-CoV-2 (Somovilla et al., 2023; García-Crespo et al., 2024). In that prior study, we reported the mutant spectrum composition of residues 22,872–23,645 (also with a mutation and deletion frequency cutoff of 0.1%) of 11 populations of USA-WA1/2020. This virus was subjected to a single passage in the absence of drugs or in the presence of either Rdv or Rib alone (four samples treated with Rdv or Rib at different concentrations) or in the presence of a combination of Rdv and Rib (four samples). The consensus genomic sequences of the different populations confirmed their minimal diversification relative to the parental USA-WA1/2020 virus (Figure 1B). The ultra-deep sequencing results of the S-coding region of these laboratory populations were previously reported (García-Crespo et al., 2024). Here, we have re-analyzed the same data in search of clade-discordant residues, with the same inclusion criteria used for the mutant spectrum of patients’ virus.

Interestingly, no clade-discordant residues that fulfilled our inclusion criteria were present in any of the three populations obtained in the infection in the absence of drugs. In contrast, clade-discordant amino acids (but not clade-discordant deletions) were present in 2 out of 4 populations obtained in the presence of one of the drugs and in 3 out of 4 populations obtained in the presence of the two drugs (Table 3) (the complete list of haplotypes for each sample is shown in Supplementary Tables S15–S17). There is a tendency (that borders on statistical significance) for the amplicons retrieved from virus replicated in the presence of drugs to exhibit more clade-discordant residues than amplicons from virus replicated in the absence of drugs (*p* = 0.057, proportion test).

**Table 3:** Amino acids in the spike SARS-CoV-2 mutant spectra that are typical of a different viral clade in SARS-CoV-2 USA-WA1/2020 populations following an infection in the presence of remdesivir (Rdv) and/or ribavirin (Rib)^a^.

Population^b^	Spike amplicon^c^	Haplotype number (frequency %)^d^	Discordant amino acid^e^	Discordant lineage/clade^f^
Rdv 10 μM	A5	27 (0.11)	Q498R	Omicron (BA.1/21K; BA.2/21L; BA.4/22A; BA.5/22B; BA.2.12.1/22C; BA.2.75/22D; BQ.1/22E; XBB/22F; XBB.1.5/23A; XBB.1.16/23B)
	A6	1/4 (30.05/2.19)	H655Y	Gamma/20J; Omicron (BA.1/21K; BA.2/21L; BA.4/22A; BA.5/22B; BA.2.12.1/22C; BA.2.75/22D; BQ.1/22E; XBB/22F; XBB.1.5/23A; XBB.1.16/23B)
Rib 100 μM	A6	9 (0.23)	D614G	All variants
		1/10/40 (16.21/0.22/0.11)	H655Y	Gamma/20J; Omicron (BA.1/21K; BA.2/21L; BA.4/22A; BA.5/22B; BA.2.12.1/22C; BA.2.75/22D; BQ.1/22E; XBB/22F; XBB.1.5/23A; XBB.1.16/23B)
Rdv 2.5 μM + Rib 80 μM	A5	5 (0.11)	Q498R	Omicron (BA.1/21K; BA.2/21L; BA.4/22A; BA.5/22B; BA.2.12.1/22C; BA.2.75/22D; BQ.1/22E; XBB/22F; XBB.1.5/23A; XBB.1.16/23B)
		*4 (0.21)	N440K + K444T + L452R + N460K + S477N + T478K + E484A + F486V + Q498R + N501Y + Y505H	Omicron BQ.1/22E
	A6	1 (13.66)	H655Y	Gamma/20J; Omicron (BA.1/21K; BA.2/21L; BA.4/22A; BA.5/22B; BA.2.12.1/22C; BA.2.75/22D; BQ.1/22E; XBB/22F; XBB.1.5/23A; XBB.1.16/23B)
		*4 (0.21)	D614G + H655Y + N679K + P681H	Omicron (BA.1/21K; BA.2/21L; BA.4/22A; BA.5/22B; BA.2.12.1/22C; BA.2.75/22D; BQ.1/22E; XBB/22F; XBB.1.5/23A; XBB.1.16/23B)
Rdv 2.5 μM + Rib 100 μM	A5	*10 (0.14)	E484K + N501Y	Beta/20H; Gamma/20J; Mu/21H
	A6	1 (14.62)	H655Y	Gamma/20J; Omicron (BA.1/21K; BA.2/21L; BA.4/22A; BA.5/22B; BA.2.12.1/22C; BA.2.75/22D; BQ.1/22E; XBB/22F; XBB.1.5/23A; XBB.1.16/23B)
		*11 (0.14)	D614G + P681H	20A; 20B; Alpha/20I; Mu/21H; Omicron (BA.1/21K; BA.2/21L; BA.4/22A; BA.5/22B; BA.2.12.1/22C; BA.2.75/22D; BQ.1/22E; XBB/22F; XBB.1.5/23A; XBB.1.16/23B)
Rdv 5 μM + Rib 100 μM	A5	19 (0.11)	Q493R	Omicron (BA.1/21K; BA.2/21L; BA.2.12.1/22C; BA.2.75/22D; BQ.1/22E; XBB/22F; XBB.1.5/23A; XBB.1.16/23B)
		21 (0.10)	Q498R	Omicron (BA.1/21K; BA.2/21L; BA.4/22A; BA.5/22B; BA.2.12.1/22C; BA.2.75/22D; BQ.1/22E; XBB/22F; XBB.1.5/23A; XBB.1.16/23B)
		20 (0.11)	T547K	21 K/Omicron, BA.1
		*6 (0.22)	N440K + G446S + S477N + T478K + E484A + Q493R + G496S + Q498R + N501Y + Y505H + T547K	Omicron BA.1/21K
	A6	1/7 (10.84/1.64)	H655Y	Gamma/20J; Omicron (BA.1/21K; BA.2/21L; BA.4/22A; BA.5/22B; BA.2.12.1/22C; BA.2.75/22D; BQ.1/22E; XBB/22F; XBB.1.5/23A; XBB.1.16/23B)

a Only cases with two or more discordant amino acids in the same or different amplicons are tabulated; no deletions were identified. The complete list of haplotypes in the two amplicons of the S-coding region for each sample is given in Supplementary Tables S15–S17.

b Populations resulting from infection of Vero E6 in presence of the indicated concentrations of Rdv and/or Rib. The virus belongs to clade 19B according to Nextstrain (https://nextstrain.org/ncov/gisaid/global).

c The S amplicons covered the following residues: A5: nucleotides 22,827–23,268; A6: nucleotides 23,259–23,645. These two amplicons spanned the amino acid residues 438–694.

d Asterisks indicate haplotypes that included two clade-discordant amino acids. The sequence of the haplotypes identified in the present analysis is given in Supplementary Tables S15–S17. The frequency of the haplotype has been calculated as percentage of all clean reads, given by the ultra-deep sequencing procedure that attained a 0.1% mutation frequency cut-off (see Materials and Methods). Different haplotypes that contain the same discordant amino acid are separated by a tilted dash.

e Amino acids that are not represented in the consensus sequence of the isolate (clade/lineage as explained in footnote c), and that are present in the clades/lineages indicated in the next column (footnote f).

f The discordant clades/lineages in which their consensus sequence includes the amino acid indicated in the previous column (footnote e).

**Figure 3:**
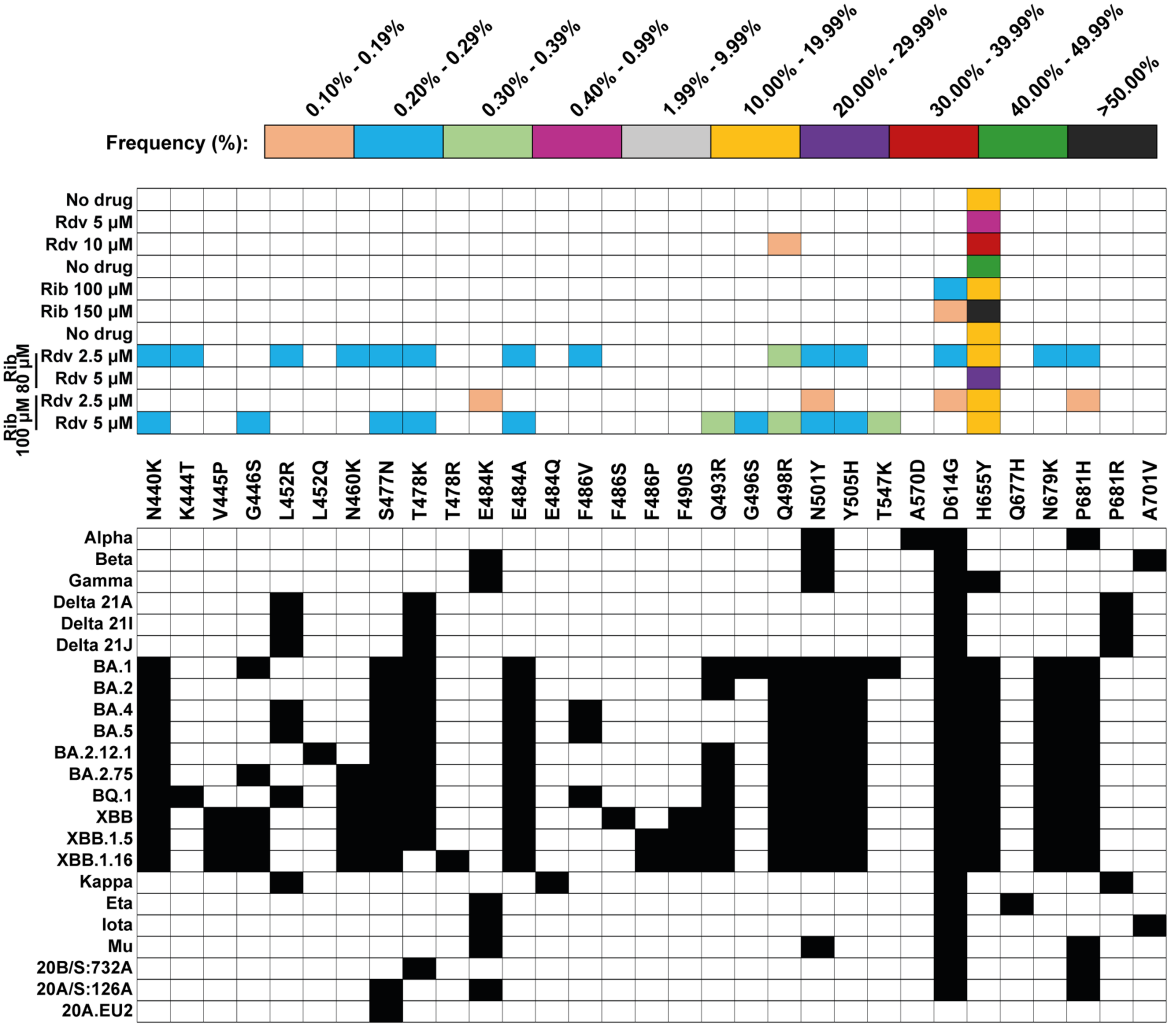
Heat map of all clade-discordant amino acid substitutions found in the S-coding region of virus from SARS-CoV-2 USA-WA1/2020 in absence or presence of drug (Rdv, remdesivir; Rib, ribavirin). The top box gives the color code for the frequency of each substitution (indicated between the two grid panels); no deletions were identified in the mutant spectrum of these populations. Drug concentration is given on the left. The bottom grid panel depicts as filled squares the substitutions used to define lineages and sub-lineages (according to https://clades.nextstrain.org) (region covered by A5 and A6 amplicons of S-coding region). The subset of clade discordant amino acids that meet the inclusion criteria detailed in the text are listed in Table 3. All passage series in the absence and presence of drugs, and all titrations for virus quantifications were carried out in triplicate (*n* = 3) (García-Crespo et al., 2024). Differences in infectious progeny production between absence and presence of drugs were statistically significant for Rdv 10 μM, Rib 150 μM, and for the combination of Rdv 5 μM + Rib 100 μM; Kruskal-Wallis with Dunn’s multiple comparison test.

Most clade-discordant amino acids (93.7% of the total) were diagnostic of Omicron lineages, but some corresponded to Alpha, Beta, Gamma, or Mu lineages. The search criterion was met even for 5 out of 20 different haplotypes identified (highlighted in Table 3). A heat map overview of the individual substitutions (Figure 3) shows a wider range of mutation frequencies than for the patient’s isolates (compared with Figure 2).

The conclusion is that multiple clade-discordant haplotypes, in particular several that have been assigned to Omicron lineages, were detected at frequencies of 0.10–30.05% in the S-mutant spectrum of SARS-CoV-2 USA-WA1/2020 populations, resulting from the infection of Vero E6 in the presence of Rdv, Rib, or both (Table 3). The presence of clade-discordant residues in laboratory populations renders unnecessarily that their presence in virus from infected patients is attributed to co-circulating cryptic lineages that merged with the major viral lineage in infected patients.

## Discussion

At least two (and often multiple) clade-discordant amino acids or deletions have been found in the mutant spectrum of virus from several COVID-19 patients and in laboratory populations that resulted from a single infection of Vero E6 cells by the USA-WA1/2020 isolate in the presence of Rdv and Rib. The authenticity of such clade-discordant traits rest on two major requisites: (i) the reliability of mutation and haplotype identification using the cutoff frequency value of 0.1% for point mutations and deletions, and (ii) that the clade-discordant residues are not the result of laboratory contamination of a pure clade virus by small amount of virus from other clades. We have carried out several experimental and bioinformatics controls to ascertain the reliability of the 0.1% cutoff frequency and evaluate the likelihood of laboratory contamination; they are presented in Materials and Methods. The present study on clade-discordant residues contributes additional arguments to sequencing reliability. Specifically, for the patients’ isolates, the discordant clades never belonged to Gamma (20 J), Iota (21F), or Lambda (21G) lineages, while 10 of them corresponded to the Alpha (20I) lineage and 9 corresponded to the Eta (21D) lineage (data in Table 2). However, the total number of synonymous and non-synonymous mutations (relative to the Wuhan-Hu-1 genomic sequence) that have been used to define lineages Gamma (20 J), Iota (21F), Lambda (21G), Alpha (20I), and Eta (21D) are 34, 22, 35, 31, and 33, respectively.^7^ The observed bias in discordant clade assignment is incompatible with sequencing mistakes being responsible for the detection of clade-discordant residues. The repeated presence of clade-discordant amino acids cannot be considered a chance occurrence since the probability of mutating toward a specific amino acid is exceedingly low. The number of amino acids encoded by a typical amplicon (approximately 400 nucleotides) in our ultra-deep sequencing analyses (see Materials and Methods) is approximately 133. Assuming a random occurrence of mutations, with the restriction that any of the 133 amino acid positions may be occupied by a maximum of 4 different amino acids, the probability of obtaining a specific amino acid by mutation is very low (1.19 × 10^−80^); if no restrictions on amino acid occupation are implemented, the probability is close to negligible (1 × 10^−173^). Therefore, some sequences appear to be favored among the vast number of those that are theoretically possible (Domingo et al., 2023).

In the laboratory populations derived from USA-WA1/2020, where they were found, the discordant clades never belonged to Delta (21A, 21I, and 21J), Kappa (21B), Eta (21D), Iota (21F), or Lambda (21G) lineages, while the number of mutations (counted relative to the Wuhan-Hu-1 virus) used to define these clades is similar to the number of mutations used to define clade Gamma (20J)^8^ (data in Table 3). A role of the mutation rate in the generation of clade-discordant residues (as opposite to alternative mechanisms based on the presence of low-frequency cryptic lineages) is suggested by their occurrence only in populations replicated in the presence of drugs. Mainly Rib, but also to some extent Rdv, exert a mutagenic activity on SARS-CoV-2 (García-Crespo et al., 2024). The increased mutation input imposed by Rdv and Rib may have pushed sequence space exploration toward genomic sequences similar to those of distant clades.

Regarding cross-contamination among samples at the time of nasopharyngeal swab collection, their handling and transport to the BSL-3 facility, or later during RNA extraction, amplification, and sequencing, the experimental controls performed and precautions to prevent contaminations are presented in Materials and Methods. No Omicron lineages were circulating at the time when viruses from patients Pt454 to Pt463 were collected or when the working stock of USA-WA1/2020 was prepared. For patient Pt500, half of the clade-discordant Omicron sequences differ from those represented in the consensus sequences of Omicron isolates that were analyzed in our laboratory.

The two main possibilities for the origin of the clade-discordant residues (Tables 2, 3) are: (i) they were present in the viruses that infected the patients we studied, as well as in the USA-WA1/2020 sample; in both cases as a result of unnoticed co-infections, or (ii) they were produced as a result of intra-host evolution in patients and infection in the presence of Rdv and Rib, respectively. We next consider possibility (ii), since possibility (i) merely relocates the same problem to a previous infection.

The issue is whether the virus mutation rate and the ensuing population complexity (combined with the selective coefficients of genomes with mutation and deletion clusters) are compatible with intra-host evolution, producing the observed clade-discordant sequences. The basic multiplication parameters of SARS-CoV-2 in vivo, and the broad cell and tissue tropism of the virus (Trypsteen et al., 2020; Sender et al., 2021; Barthe et al., 2023) render difficult to estimate the number of replication rounds that the virus underwent prior to its excretion into the nasopharyngeal fluid where it was collected. For the laboratory populations, considering the input and final virus titer (García-Crespo et al., 2024), the number of net genome doublings can be estimated in a maximum of 10, a value that was probably exceeded in the virus from patients. Previous data with other RNA viruses render realistic that with such an estimate of replication rounds, if genomes with clade-discordant residues displayed a modest selective advantage over the resident virus, they could become a detectable part of the mutant spectrum. Previous evidence of effective selection in other RNA viruses include the precise and rapid reversion in different clonal poliovirus populations of four silent mutations that were engineered in the poliovirus genome (de la Torre et al., 1992), and the rapid and systematic selection of foot-and-mouth disease virus (FMDV) mutants with two amino acid substitutions in the viral capsid in a single infection of a BHK-21 cell line that posed some resistance to the wild type virus (Escarmís et al., 1998). Thus, divergent genomes with mutation clusters are within the scope of reported selective sweeps described in RNA viruses.

To what extent the 3′ to 5′ exonuclease domain included in protein nsp14 is effective in reducing the error rate during infection is unknown. However, the heterogeneity—and therefore the potential of sequence space exploration—of SARS-CoV-2 is comparable to that displayed by mutant spectra of other RNA viruses that have been analyzed by comparable methodology (Domingo et al., 2021a, 2023; Martínez-González et al., 2022a). Selection of mutation clusters of the type described for other RNA viruses (de la Torre et al., 1992; Escarmís et al., 1998), followed by recombination events (Jackson et al., 2021; Sekizuka et al., 2022), may give rise to genomes carrying the clade-discordant residues. Some of the recombinants may display sufficient fitness to be maintained in the population. Random drift of the genomes (through bottleneck events), and acquisition of additional fitness enhancing mutations, may result in becoming epidemiologically dominant. This model is compatible with mechanisms that operate in RNA viral quasispecies dynamics and that drive short-term virus evolution (Domingo, 2020; Domingo et al., 2021b, 2023; Sun et al., 2021; Harari et al., 2022).

The observations reported in the present study are in agreement that out of the vast sequence space available to SARS-CoV-2, only a limited portion can be actually occupied. Ongoing work shows the repetition of haplotype sequences in different patients, suggesting the intriguing possibility that mutant spectrum composition may play a role as watchtower of permitted genomic sequences of the virus as it spreads in the human population (Domingo et al., 2023). This possibility has, as precedent, the observation that in the course of fitness gain of low fitness FMDV biological clones, mutant spectra included mutations that became dominant at a later stage of the evolution of the virus (Arias et al., 2001). The term “harbinger” was coined to refer to mutations with predictive value of evolutionary pathways (Domingo, 2020). Given the millions of consensus sequences deposited in data banks (i.e., in NCBI, ENA, and GISAID), and the feasibility of high-resolution mutant spectrum analysis, SARS-CoV-2 offers interesting prospects to evaluate the predictive usefulness of mutant spectrum composition.

## Data availability statement

The data presented in the study are deposited in the European Nucleotide Archive (ENA) repository, under accession numbers PRJEB49400, PRJEB64922 and PRJEB66908.

## Ethics statement

The studies involving humans were approved by the Institutional Review Board of the University Hospital Fundación Jiménez Díaz (no. PIC166-21-FJD and PIC080-23-FJD). The institutional Bioethics Committee from Consejo Superior de Investigaciones Científicas (CSIC), in accordance with Spanish regulations, also approved the study (no. 082/2021). The studies were conducted in accordance with the local legislation and institutional requirements. The human samples used in this study were acquired from primarily isolated as part of your previous study for which ethical approval was obtained. Written informed consent for participation was not required from the participants or the participants’ legal guardians/next of kin in accordance with the national legislation and institutional requirements.

## Author contributions

BM-G: Formal analysis, Investigation, Methodology, Writing – original draft, Writing – review & editing. MS: Formal analysis, Investigation, Writing – review & editing. PM: Formal analysis, Software, Writing – review & editing. RL-R: Formal analysis, Software, Writing – review & editing. LS-V: Data curation, Formal analysis, Writing – review & editing. AL-G: Data curation, Formal analysis, Writing – review & editing. ME-M: Methodology, Writing – review & editing. AD-P: Formal analysis, Methodology, Writing – review & editing. PS: Formal analysis, Methodology, Writing – review & editing. CG-C: Formal analysis, Methodology, Writing – review & editing. AÁ: Formal analysis, Methodology, Writing – review & editing. JG: Formal analysis, Software, Writing – review & editing. JE: Data curation, Formal analysis, Writing – review & editing. RF-R: Data curation, Formal analysis, Writing – review & editing. IG: Data curation, Formal analysis, Writing – review & editing. ED: Conceptualization, Funding acquisition, Investigation, Project administration, Resources, Supervision, Writing – original draft, Writing – review & editing. CP: Conceptualization, Funding acquisition, Investigation, Project administration, Resources, Supervision, Writing – original draft, Writing – review & editing.

## Footnotes

^1^ https://www.who.int/activities/tracking-SARS-CoV-2-variants

^2^ https://clades.nextstrain.org/

^3^ https://basespace.illumina.com

^4^ https://clades.nextstrain.org/

^5^ https://covariants.org/

^6^ https://emea.illumina.com/systems/sequencing-platforms/miseq/specifications.html

^7^ https://clades.nextstrain.org/

^8^ https://clades.nextstrain.org/
