# Supplementary material for: SARS-CoV-2 mutant spectra as variant of concern nurseries: endless variation?
Source: Front Microbiol. 2024 Mar 14;15:1358258. doi: 10.3389/fmicb.2024.1358258 (PMC10979541; doi:10.3389/fmicb.2024.1358258)
Supplement: Supplementary file 1 [file Data_Sheet_1.PDF]

Supplemental Material for:

SARS-CoV-2 mutant spectra as variant of concern nurseries: endless variation?

Brenda Martínez-González[1,2], María Eugenia Soria[2,3], Pablo Mínguez[4,5,6], Ramón Lorenzo-Redondo[7], Llanos Salar-Vidal[2,8], Alberto López-García[9], Mario Esteban-Muñoz[2], Antoni Durán-Pastor[1], Pilar Somovilla[3,10], Carlos García-Crespo[3], Ana Isabel de Ávila[3], Jordi Gómez[11], Jaime Esteban[2,8], Ricardo Fernández-Roblas[2,8], Ignacio Gadea[2,8], Esteban Domingo[3]* and Celia Perales[1,2]*

[1]Department of Molecular and Cell Biology, Centro Nacional de Biotecnología (CNB-CSIC), Consejo Superior de Investigaciones Científicas (CSIC), Campus de Cantoblanco, Madrid, Spain, [2]Department of Clinical Microbiology, Instituto de Investigación Sanitaria-Fundación Jiménez Díaz University Hospital, Universidad Autónoma de Madrid (IIS-FJD, UAM) Av. Reyes Católicos 2, Madrid, Spain, [3]Centro de Biología Molecular "Severo Ochoa" (CSIC-UAM), Campus de Cantoblanco, Madrid, Spain, [4]Department of Genetics & Genomics, Instituto de Investigación Sanitaria-Fundación Jiménez Díaz University Hospital, Universidad Autónoma de Madrid (IIS-FJD, UAM), Av. Reyes Católicos 2, Madrid, Spain, [5]Centre for Biomedical Network Research on Rare Diseases (CIBERER), Instituto de Salud Carlos III, Madrid, Spain, [6]Bioinformatics Unit, Instituto de Investigación Sanitaria-Fundación Jiménez Díaz University Hospital, Universidad Autónoma de Madrid (IIS-FJD, UAM), Madrid, Spain, [7]Division of Infectious Diseases, Center for Pathogen Genomics and Microbial Evolution, Feinberg School of Medicine, Northwestern University, Chicago, IL, USA, [8]Centre for Biomedical Network Research on Infectious Diseases (CIBERINFEC), Madrid, Spain, [9]Health Research Institute IIS-FJD, Fundación Jiménez Diaz University Hospital, Madrid, Spain, [10]Departamento de Biología Molecular, Universidad Autónoma de Madrid, Campus de Cantoblanco, , Madrid, Spain,, [11]Instituto de Parasitología y Biomedicina 'López-Neyra' (CSIC), Parque Tecnológico Ciencias de la Salud, Armilla, Granada, Spain

*Correspondence:

Celia Perales, celia.perales@cnb.csic.es

Esteban Domingo, edomingo@cbm.csic.es

Table S1. Specific oligonucleotides designed to amplify and sequence the spike (S)-coding region of SARS-CoV-2.

Oligonucleotide name	Sense	Sequence (5'-3')	Genome positions[a]	Tm (ºC)[b]
**Spk-CoV2-u21424**	Fw	GGTACTGCTGTTATGTCTTTAAAA	21424-21447	50.6
**Spk-CoV2-d21867**	Rv	CTTATTATGTTAGACTTCTCAGTGGA	21867-21842	53.2
**Spk-CoV2-u21701**	Fw	GTTTTACATTCAACTCAGGACTTGTT	21701-21726	53.2
**Spk-CoV2-d22154**	Rv	CAATATTCTTAAACACAAATTCCCAA	22154-22128	50.6
**Spk-CoV2-u22088**	Fw	CTTATGGACCTTGAAGGAAAACA	22088-22110	51.7
**Spk-CoV2-d22536**	Rv	GATTCTGTTGGTTGGACTCTA	22536-22516	50.5
**Spk-CoV2-u22464**	Fw	GTACGTTGAAATCCTTCACTGTA	22464-22486	51.7
**Spk-CoV2-d22902**	Rv	CCACCAACCTTAGAATCAAGAT	22902-22881	51.1
**Spk-CoV2-u22853**	Fw	GGCTGCGTTATAGCTTGGA	22853-22871	51.1
**Spk-CoV2-d23288**	Rv	CAGCATCAGTAGTGTCAGCA	23288-23269	51.8
**Spk-CoV2-u23236**	Fw	GTTTCTGCCTTTCCAACAATTTG	23236-23258	51.7
**Spk-CoV2-d23666**	Rv	CTGCACCAAGTGACATAGTGT	23666-23646	52.4

[a]The SARS-CoV-2 residue numbering is according to the NCBI reference sequence (NC_045512.2)

[b]Tm: Temperature of melting.

Table S2. Number of clean reads obtained in samples from SARS-CoV-2-infected patients, and in samples from SARS-CoV-2 USA-WA1/2020 populations following an infection in the absence and presence of remdesivir (Rdv) and/or ribavirin (Rib) using a haplotype frequency cut-off of 0.1%.

	Spike amplicons						
**Sample ID**	**A1**	**A2**	**A3**	**A4**	**A5**	**A6**	**Total**
**Pt454**	98,034	79,759	113,547	82,898	55,047	53,590	**482,875**
**Pt455**	37,154	55,772	105,885	45,112	141,538	220,457	**605,918**
**Pt456**	210,689	59,321	65,460	32,390	74,475	86,410	**528,745**
**Pt457**	144,781	55,892	39,526	33,077	77,340	67,113	**417,729**
**Pt458**	83,065	109,296	140,443	89,832	71,487	49,741	**543,864**
**Pt459**	108,799	94,024	124,724	55,890	65,717	121,445	**570,599**
**Pt460**	95,805	78,841	102,103	100,169	119,463	110,652	**607,033**
**Pt461**	46,561	47,220	63,267	137,852	253,780	59,996	**608,676**
**Pt462**	117,917	40,916	201,469	49,799	67,029	52,932	**530,062**
**Pt463**	103,118	73,028	99,776	91,096	128,538	94,214	**589,770**
**Pt500**	85,436	73,577	90,055	69,086	72,072	89,868	**480,094**
**No drug**	nd	nd	nd	nd	125,026	107,985	**233,011**
**Rib 100 µM**	nd	nd	nd	nd	99,677	105,558	**205,235**
**Rib 150 µM**	nd	nd	nd	nd	145,938	122,238	**268,176**
**No drug**	nd	nd	nd	nd	97,245	84,190	**181,435**
**Rdv 5 µM**	nd	nd	nd	nd	117,824	111,830	**229,654**
**Rdv 10 µM**	nd	nd	nd	nd	93,504	104,556	**198,060**
**No drug**	nd	nd	nd	nd	139,957	131,571	**271,528**
**Rdv 2.5 µM + Rib 80 µM**	nd	nd	nd	nd	145,025	138,635	**283,660**
**Rdv 5 µM + Rib 80 µM**	nd	nd	nd	nd	115,035	115,607	**230,642**
**Rdv 2.5 µM + Rib 100 µM**	nd	nd	nd	nd	141,097	119,325	**260,422**
**Rdv 5 µM + Rib 100 µM**	nd	nd	nd	nd	121,174	126,115	**247,289**

nd: no data

Table S3. Repertoire of mutations, amino acid substitutions, and deleted positions detected in the consensus sequence of samples studied using COVIDSeq (Illumina).

			Sample ID										
**Region[a]**	**Mutation[b]**	**Amino acid substitution[c]**	**Pt 454**	**Pt 455**	**Pt 456**	**Pt 457**	**Pt 458**	**Pt 459**	**Pt 460**	**Pt 461**	**Pt 462**	**Pt 463**	**Pt 500**
**5'UTR**	G174T	-											Yes
	G204A	-		Yes		Yes							
	C241T	-	Yes	Yes	Yes	Yes	Yes	Yes	Yes	Yes	Yes	Yes	Yes
**nsp1**	C281T	P6S					Yes						
	T445C	Syn						Yes	Yes		Yes		
	G458A	E65K									Yes		
	C664T	Syn											Yes
**nsp2**	C913T	Syn	Yes	Yes	Yes	Yes	Yes						
	G922T	Syn							Yes				
	C1059T	T265I								Yes			Yes
	A1072G	Syn						Yes					
	A1180G	Syn								Yes			
	G1439T	G392C							Yes				
	G1463T	G400C											Yes
	T1861C	Syn									Yes		
	T2083C	Syn							Yes				
	G2095A	Syn									Yes		
	C2110T	Syn			Yes								
	A2692T	Syn											Yes
**nsp3**	C3037T	Syn	Yes	Yes	Yes	Yes	Yes	Yes	Yes	Yes	Yes	Yes	Yes
	G3091T	E942D									Yes		
	A3111G	Y949C	Yes										
	C3177T	P971L	Yes										
	C3267T	T1001I	Yes	Yes	Yes	Yes	Yes						
	T3268C	Syn										Yes	
	G4444T	Syn	Yes										
	C4543T	Syn										Yes	
	T4813C	Syn		Yes									
	C5170T	Syn						Yes					
	G5230T	K1655N											Yes
	C5388A	A1708D	Yes	Yes	Yes	Yes	Yes						
	G5629T	Syn										Yes	
	C5730T	T1822I									Yes		
	C5812T	Syn							Yes				
	C5986T	Syn	Yes	Yes	Yes	Yes	Yes						
	C6286T	Syn						Yes	Yes		Yes		
	C6354T	S2030L									Yes		
	C6730T	Syn								Yes			
	A6851C	T2196P								Yes			
	C6936T	S2224F								Yes			
	T6954C	I2230T	Yes	Yes	Yes	Yes	Yes						

	C7749T	T2495I								Yes			
	C7998T	S2578F										Yes	
**nsp4**	G9111A	R2949H									Yes		
	C9142T	Syn											Yes
	C9226T	Syn								Yes			
	G9526T	M3087I										Yes	
	C10029T	T3255I								Yes			
**nsp5**	A10323G	K3353R											Yes
	C10645T	Syn								Yes			
**nsp6**	A11089T	E3608D							Yes				
	A11090T	N3609Y							Yes				
	G11132T	A3623S						Yes					
	A11353C	Syn						Yes					
	C11497T	Syn										Yes	
	C11514T	T3750I								Yes			
	T11515C	Syn										Yes	
**nsp8**	C12676T	Syn						Yes					
**nsp12**	G13993T	A176S										Yes	
	C14120T	P218L			Yes								
	C14408T	P314L	Yes	Yes	Yes	Yes	Yes	Yes	Yes	Yes	Yes	Yes	Yes
	C14676T	Syn	Yes	Yes	Yes	Yes	Yes						
	C15279T	Syn	Yes	Yes	Yes	Yes	Yes						
	G15766T	V767L										Yes	
	T16176C	Syn	Yes	Yes	Yes	Yes	Yes						
**nsp13**	C16260T	Syn									Yes		
	C16338T	Syn											Yes
	C16375T	P970S								Yes			
	C16466T	P1000L										Yes	
	A16889G	K1141R										Yes	
	G16943T	S1159I							Yes				
	G17019T	E1184D										Yes	
	A17615G	K1383R					Yes						
**nsp14**	C18060T	Syn								Yes			
	C18657T	Syn											Yes
	C18877T	Syn										Yes	
	C19019A	P1851Q											Yes
	A19079G	E1871G					Yes						
	C19151T	A1895V									Yes		
	G19518T	L2017F		Yes		Yes							
	C19524T	Syn							Yes				
**nsp15**	C19854T	Syn					Yes						
	C20016T	Syn							Yes				
**nsp16**	T20748C	Syn								Yes			
	G21255C	Syn						Yes	Yes		Yes		
**spike**	T21579C	V6A					Yes						
	C21614T	L18F											Yes

	A21801C	D80A											Yes
	G21898A	Syn						Yes					
	A22206G	D215G											Yes
	C22227T	A222V						Yes	Yes		Yes		
	A22488G	E309G							Yes				
	A22622C	N354H							Yes				
	G22813T	K417N											Yes
	G22992A	S477N										Yes	
	G23012A	E484K											Yes
	T23042C	S494P								Yes			
	A23063T	N501Y	Yes	Yes	Yes	Yes	Yes						Yes
	C23271A	A570D	Yes	Yes	Yes	Yes	Yes						
	C23277T	T572I									Yes		
	A23403G	D614G	Yes	Yes	Yes	Yes	Yes	Yes	Yes	Yes	Yes	Yes	Yes
	C23604A	P681H	Yes	Yes	Yes	Yes	Yes			Yes			
	C23664T	A701V											Yes
	C23709T	T716I	Yes	Yes	Yes	Yes	Yes			Yes			
	C24109T	Syn											Yes
	G24197T	A879S							Yes				
	C24334T	Syn											Yes
	T24506G	S982A	Yes	Yes	Yes	Yes	Yes						
	G24764T	V1068F									Yes		
	G24914C	D1118H	Yes	Yes	Yes	Yes	Yes						
**ORF3a**	G25563T	Q57H								Yes		Yes	Yes
	G25660A	V90I											Yes
	C25710T	Syn										Yes	
	C25844T	T151I											Yes
	C25904T	S171L						Yes					Yes
**E**	C26456T	P71L											Yes
**M**	C26645T	Syn											Yes
	C26735T	Syn										Yes	
	T26767C	I82T								Yes			
	C26801G	Syn						Yes	Yes		Yes		
	T26876C	Syn										Yes	
	G27014T	Syn									Yes		
**ORF7a**	C27630T	Syn				Yes							
	G27659T	R89I										Yes	
**ORF8**	C27944T	Syn							Yes		Yes		
	C27972T	Q27*	Yes	Yes	Yes	Yes	Yes						
	G28027T	W45L										Yes	
	G28048T	R52I	Yes	Yes	Yes	Yes	Yes						
	A28095T	K68*			Yes								
	A28111G	Y73C	Yes	Yes	Yes	Yes	Yes						
	T28135C	V81A						Yes					
	C28253T	Syn											Yes
**N**	G28280C	D3L	Yes	Yes	Yes	Yes	Yes						

	A28281T		Yes	Yes	Yes	Yes	Yes						
	T28282A		Yes	Yes	Yes	Yes	Yes						
	C28291T	Syn							Yes				
	G28307T	A12S						Yes					
	C28310A	P13T										Yes	
	C28453T	Syn						Yes					
	C28751A	Q160K						Yes					
	G28881A	R203K	Yes	Yes	Yes	Yes	Yes						
	G28882A		Yes	Yes	Yes	Yes	Yes						
	G28883C	G204R	Yes	Yes	Yes	Yes	Yes						
	C28887T	T205I								Yes			Yes
	C28932T	A220V						Yes	Yes		Yes		
	G28975C	M234I										Yes	
	C28977T	S235F	Yes	Yes	Yes	Yes	Yes						
	G29399A	A376T										Yes	
	A29406T	E378V											Yes
	G29513T	A414S								Yes			
**ORF9b**	C28291T	P3L							Yes				
	G28307T	M8I						Yes					
	C28310A	H9Q										Yes	
	C28453T	A57V						Yes					
**Non coding region**	C29541T	-					Yes						
**ORF10**	C29614T	Syn								Yes			
	G29645T	V30L						Yes	Yes		Yes		
**3'UTR**	G29706C	-			Yes								
	C29719T	-								Yes			

			**Sample ID**										
**Region[a]**	**Positions deleted[b]**	**Amino acid deleted[c]**	**454**	**455**	**456**	**457**	**458**	**459**	**460**	**461**	**462**	**463**	**500**
**nsp6**	11288-11296	S3675Δ, G3676Δ, F3677Δ	Yes	Yes	Yes	Yes	Yes						Yes
**spike**	21765-21770	H69Δ, V70Δ	Yes	Yes	Yes	Yes	Yes						
	22283-22291	L241Δ, L242Δ, A243Δ											Yes
	21992-21994	Y144Δ	Yes	Yes	Yes	Yes	Yes						
**Non coding region**	28271	-	Yes	Yes	Yes	Yes	Yes			Yes			
**3'UTR**	29711	-								Yes			

[a]Genomic region under analysis.

[b]Genome residue numbering of SARS-CoV-2 is according to the NCBI reference sequence (accession number NC_045512.2).

[c]Amino acid residues are numbered from N- to the C- terminus of each open reading frame (ORF). Genes nsp1 up to nsp8 are numbered according to ORF1a and genes nsp12 up to nsp16 are numbered according to ORF1b.

Table S4. Haplotypes detected in amplicons A1 to A6 of S-coding region in sample from patient Pt454[a].

Spike A1

Haplotype number	Frequency	Nucleotide mutation[b]	Amino acid substitutions[c]	Nucleotide deletions[b]	Deleted amino acids[c]
Hpl 0	91.61%			21765-21770	H69Δ, V70Δ
Hpl 1	0.62%	C21557T	Syn		
Hpl 2	0.41%	T21737C	F59L		
Hpl 3	0.29%	T21596C	S12P		
Hpl 4	0.28%	A21779G	T73A		
Hpl 5	0.27%	T21475C	ORF1b:S2670P		
Hpl 6	0.27%	A21720G	D53G		
Hpl 7	0.26%	T21755C	F65L		
Hpl 8	0.26%	T21540C	Syn		
Hpl 9	0.25%	A21825G	D88G		
Hpl 10	0.24%	T21655C	Syn		
Hpl 11	0.24%	A21794G	R78G		
Hpl 12	0.24%	T21477C	Syn		
Hpl 13[d]	0.24%	C21575T	L5F		
Hpl 14	0.23%	A21489G	Syn		
Hpl 15	0.22%	T21542C	ORF1b:L2692P		
Hpl 16	0.22%	A21519G	Syn		
Hpl 17	0.22%	T21733C	Syn		
Hpl 18	0.22%	A21560G	Syn		
Hpl 19	0.22%	A21685G	Syn		
Hpl 20	0.21%	T21566C	F2L		
Hpl 21[d]	0.21%	A21717G	Q52R		
Hpl 22	0.21%	A21495G	Syn		
Hpl 23	0.21%	A21450G	Syn		
Hpl 24	0.21%	T21479C	ORF1b:L2671P		
Hpl 25	0.20%	T21702C	V47A		
Hpl 26	0.20%	A21792G	K77R		
Hpl 27	0.20%	T21722C	Syn		
Hpl 28	0.20%	T21594C	V11A		
Hpl 29	0.19%	T21539C	ORF1b:V2691A		
Hpl 30	0.19%	T21601C	Syn		
Hpl 31	0.19%	T21482C	ORF1b:L2672P		
Hpl 32	0.19%	A21620G	T20A		
Hpl 33	0.19%	A21626G	T22A		
Hpl 34	0.19%	C21811T	Syn		
Hpl 35	0.18%	T21497C	ORF1b:L2677P		

Spike A2

Haplotype number	Frequency	Nucleotide mutation[b]	Amino acid substitutions[c]	Nucleotide deletions[b]	Deleted amino acids[c]
Hpl 0	88.98%			21765-21770, 21992-21994	H69Δ, V70Δ, Y144Δ
Hpl 1	0.48%	T21737C	F59L		

Hpl 2	0.45%	T21773C	S71P		
Hpl 3	0.38%	T21755C	F65L		
Hpl 4	0.36%	A22102G	Syn		
Hpl 5	0.32%	A22095G	D178G		
Hpl 6	0.32%	T21841C	Syn		
Hpl 7	0.31%	T21797C	F79L		
Hpl 8	0.30%	A22025G	S155G		
Hpl 9	0.30%	A22029G	E156G		
Hpl 10	0.29%	T21733C	Syn		
Hpl 11	0.28%	A22107G	K182R		
Hpl 12	0.27%	T21908C	S116P		
Hpl 13	0.26%	A22108G	Syn		
Hpl 14	0.26%	T21842C	S94P		
Hpl 15	0.25%	T22074C	V171A		
Hpl 16	0.25%	T21808C	Syn		
Hpl 17	0.25%	T21831C	V90A		
Hpl 18	0.24%	T21955C	Syn		
Hpl 19	0.23%	A22008G	N149S		
Hpl 20	0.23%	T21735C	F58S		
Hpl 21	0.23%	T22084C	Syn		
Hpl 22	0.23%	A22106G	K182E		
Hpl 23	0.23%	T21747C	V62A		
Hpl 24[d]	0.23%	T21810C	V83A		
Hpl 25	0.22%	A22110G	Q183R		
Hpl 26	0.22%	T21756C	F65S		
Hpl 27	0.22%	T21829C	Syn		
Hpl 28	0.22%	T21837C	F92S		
Hpl 29	0.22%	A22101G	E180G		
Hpl 30	0.22%	T21889C	Syn		
Hpl 31	0.22%	A22105G	Syn		
Hpl 32	0.21%	A21900G	K113R		
Hpl 33	0.20%	A21849G	E96G		
Hpl 34	0.20%	A22013G	S151G		
Hpl 35	0.20%	A21902G	T114A		
Hpl 36	0.20%	T21990C	V143A		
Hpl 37	0.20%	T21832C	Syn		
Hpl 38	0.19%	T21734C	F58L		
Hpl 39	0.19%	T21728C	Syn		
Hpl 40[e]	0.19%	T21771C	V70T		I68Δ, H69Δ, Y144Δ
Hpl 41	0.19%	A22019G	M153V		H69Δ, V70Δ, Y144Δ
Hpl 42	0.19%	A21957G	E132G		
Hpl 43	0.18%	T21775C	Syn		
Hpl 44	0.18%	A21868G	Syn		

Spike A3

Haplotype number	Frequency	Nucleotide mutation[b]	Amino acid substitutions[c]	Nucleotide deletions[b]	Deleted amino acids[c]

Hpl 0	89.12%	Wild type			
Hpl 1	0.41%	T22209C	L216P		
Hpl 2	0.33%	T22384C	Syn		
Hpl 3	0.32%	A22496G	I312V		
Hpl 4	0.32%	A22412G	T284A		
Hpl 5	0.31%	A22443G	D294G		
Hpl 6	0.29%	T22291C	Syn		
Hpl 7	0.29%	A22457G	T299A		
Hpl 8	0.27%	T22219C	Syn		
Hpl 9	0.27%	A22337G	T259A		
Hpl 10	0.27%	T22228C	Syn		
Hpl 11	0.25%	A22492G	Syn		
Hpl 12	0.25%	T22514C	Syn		
Hpl 13	0.24%	T22274C	F238L		
Hpl 14	0.24%	A22420G	Syn		
Hpl 15	0.23%	T22447C	Syn		
Hpl 16	0.23%	T22207C	Syn		
Hpl 17	0.22%	A22411G	Syn		
Hpl 18	0.22%	A22422G	D287G		
Hpl 19	0.22%	T22435C	Syn		
Hpl 20[d]	0.22%			22283-22291	L241Δ, L242Δ, A243Δ
Hpl 21	0.22%	T22478C	F306L		
Hpl 22	0.22%	A22461G	K300R		
Hpl 23	0.21%	T22213C	Syn		
Hpl 24	0.21%	A22234G	Syn		
Hpl 25	0.21%	T22266C	I235T		
Hpl 26	0.21%	T22324C	Syn		
Hpl 27	0.21%	T22321C	Syn		
Hpl 28	0.21%	T22483C	Syn		
Hpl 29	0.20%	A22301G	S247G		
Hpl 30	0.20%	A22135G	Syn		
Hpl 31	0.20%	T22282C	Syn		
Hpl 32	0.20%	T22292C	Syn		
Hpl 33	0.20%	A22155G	D198G		
Hpl 34	0.19%	T22142C	F194L		
Hpl 35	0.19%	A22146G	K195R		
Hpl 36	0.19%	T22322C	S254P		
Hpl 37	0.19%	T22137C	F192S		
Hpl 38	0.19%	A22453G	Syn		
Hpl 39	0.19%	A22431G	D290G		
Hpl 40	0.19%	A22474G	Syn		
Hpl 41	0.19%	A22375G	Syn		
Hpl 42	0.18%	A22148G	N196D		
Hpl 43	0.18%	A22184G	T208A		
Hpl 44	0.18%	T22174C	Syn		
Hpl 45	0.18%	T22371C	L270P		
Hpl 46	0.18%	A22473G	K304R		

Hpl 47	0.18%	A22351G	Syn		
Hpl 48	0.18%	A22455G	E298G		

Spike A4

Haplotype number	Frequency	Nucleotide mutation[b]	Amino acid substitutions[c]	Nucleotide deletions[b]	Deleted amino acids[c]
Hpl 0	91.04%	Wild type			
Hpl 1	1.58%	T22690C	Syn		
Hpl 2	0.34%	T22736C	F392L		
Hpl 3	0.33%	A22780G	Syn		
Hpl 4	0.27%	A22812G	K417R		
Hpl 5	0.27%	T22507C	Syn		
Hpl 6	0.27%	A22753G	Syn		
Hpl 7	0.26%	A22705G	Syn		
Hpl 8	0.26%	T22514C	F318L		
Hpl 9	0.26%	A22852G	Syn		
Hpl 10	0.26%	G22487C	E309Q		
Hpl 11	0.25%	A22776G	D405G		
Hpl 12	0.23%	A22786G	Syn		
Hpl 13	0.23%	A22492G	Syn		
Hpl 14	0.23%	A22623G	N354S		
Hpl 15	0.23%	A22771G	Syn		
Hpl 16	0.23%	A22629G	K356R		
Hpl 17	0.22%	A22842G	D427G		
Hpl 18	0.22%	T22501C	Syn		
Hpl 19	0.22%	T22662C	V367A		
Hpl 20	0.22%	T22607C	S349P		
Hpl 21	0.21%	T22497C	I312T		
Hpl 22	0.21%	A22784G	R408G		
Hpl 23	0.21%	T22573C	Syn		
Hpl 24	0.20%	A22628G	K356E		
Hpl 25	0.20%	A22633G	Syn		
Hpl 26	0.20%	T22515C	F318S		
Hpl 27	0.20%	T22521C	V320A		
Hpl 28	0.20%	T22746C	V395A		
Hpl 29	0.19%	T22711C	Syn		
Hpl 30	0.19%	A22631G	R357G		
Hpl 31	0.19%	A22519G	Syn		
Hpl 32	0.18%	T22542C	V327A		
Hpl 33	0.18%	T22860C	V433A		

Spike A5

Haplotype number	Frequency	Nucleotide mutation[b]	Amino acid substitutions[c]	Nucleotide deletions[b]	Deleted amino acids[c]
Hpl 0	90.00%	A23063T	N501Y		
Hpl 1	0.38%	A23063T, A23265G	N501Y, D568G		

Hpl 2	0.31%	T22873C, A23063T	Syn, N501Y
Hpl 3	0.30%	T22874C, A23063T	S438P, N501Y
Hpl 4	0.29%	A23063T, A23263G	N501Y, Syn
Hpl 5	0.26%	T22888C, A23063T	Syn, N501Y
Hpl 6	0.26%	T22942C, A23063T	Syn, N501Y
Hpl 7	0.26%	A23063T, A23203G	N501Y, Syn
Hpl 8	0.25%	T23030C, A23063T	F490L, N501Y
Hpl 9	0.25%	A23063T, A23140G	N501Y, Syn
Hpl 10	0.25%	T22937C, A23063T	S459P, N501Y
Hpl 11	0.25%	A23063T, T23112C	N501Y, L517P
Hpl 12	0.25%	A23063T, T23104C	N501Y, Syn
Hpl 13	0.24%	A23063T, A23169G	N501Y, N536S
Hpl 14	0.24%	A23063T, T23100C	N501Y, L513P
Hpl 15	0.24%	A23063T, T23245C	N501Y, Syn
Hpl 16	0.24%	T22896C, A23063T	V445A, N501Y
Hpl 17	0.23%	A23063T, T23150C	N501Y, S530P
Hpl 18	0.23%	A23063T, A23209G	N501Y, Syn
Hpl 19	0.23%	A22960G, A23063T	Syn, N501Y
Hpl 20	0.23%	T22876C, A23063T	Syn, N501Y
Hpl 21	0.23%	A23063T, A23129G	N501Y, T523A
Hpl 22	0.23%	A22958G, A23063T	R466G, N501Y
Hpl 23	0.23%	A23063T, T23215C	N501Y, Syn
Hpl 24	0.22%	T22951C, A23063T	Syn, N501Y
Hpl 25	0.22%	T22944C, A23063T	L461P, N501Y
Hpl 26	0.22%	A23063T, A23223G	N501Y, E554G
Hpl 27	0.22%	A23063T, A23122G	N501Y, Syn
Hpl 28	0.22%	A22962G, A23063T	D467G, N501Y
Hpl 29	0.22%	T23017C, A23063T	Syn, N501Y
Hpl 30	0.22%	A23063T, T23225C	N501Y, S555P

Hpl 31	0.21%	A23063T, A23251G	N501Y, Syn		
Hpl 32	0.21%	T23026C, A23063T	Syn, N501Y		
Hpl 33	0.21%	T22999C, A23063T	Syn, N501Y		
Hpl 34	0.21%	T22882C, A23063T	Syn, N501Y		
Hpl 35	0.21%	A23063T, A23207G	N501Y, T549A		
Hpl 36	0.20%	T23050C, A23063T	Syn, N501Y		
Hpl 37	0.20%	T23032C, A23063T	Syn, N501Y		
Hpl 38	0.20%	A22935G, A23063T	K458R, N501Y		
Hpl 39	0.19%	A23056G, A23063T	Syn, N501Y		
Hpl 40	0.19%	A23063T, A23089G	N501Y, Syn		
Hpl 41	0.19%	A23063T, A23261G	N501Y, R567G		
Hpl 42	0.19%	T23010C, A23063T	V483A, N501Y		
Hpl 43	0.19%	A22948G, A23063T	Syn, N501Y		

Spike A6

Haplotype number	Frequency	Nucleotide mutation[b]	Amino acid substitutions[c]	Nucleotide deletions[b]	Deleted amino acids[c]
Hpl 0	94.69%	C23271A, A23403G, C23604A	A570D, D614G, P681H		
Hpl 1	0.52%	C23271A, A23403G, C23533T, C23604A	A570D, D614G, Syn, P681H		
Hpl 2	0.48%	C23271A, A23403G, A23416T, C23604A	A570D, D614G, Syn, P681H		
Hpl 3	0.42%	C23271A, A23403G, A23544G, C23604A	A570D, D614G, E661G, P681H		
Hpl 4	0.30%	C23271A, T23332C, A23403G, C23604A	A570D, Syn, D614G, P681H		
Hpl 5	0.28%	C23271A, A23403G, A23550G, C23604A	A570D, D614G, D663G, P681H		
Hpl 6	0.27%	C23271A, A23403G, A23594G, C23604A	A570D, D614G, T678A, P681H		

Hpl 7	0.27%	C23271A, A23403G, A23588G, C23604A	A570D, D614G, T676A, P681H	
Hpl 8	0.24%	C23271A, T23350C, A23403G, C23604A	A570D, Syn, D614G, P681H	
Hpl 9	0.24%	C23271A, T23289C, A23403G, C23604A	A570D, V576A, D614G, P681H	
Hpl 10	0.24%	C23271A, A23403G, A23495G, C23604A	A570D, D614G, T645A, P681H	
Hpl 11	0.24%	C23271A, A23403G, T23452C, C23604A	A570D, D614G, Syn, P681H	
Hpl 12	0.23%	C23271A, T23346C, A23403G, C23604A	A570D, V595A, D614G, P681H	
Hpl 13	0.21%	C23271A, T23293C, A23403G, C23604A	A570D, Syn, D614G, P681H	
Hpl 14	0.21%	C23271A, T23391C, A23403G, C23604A	A570D, V610A, D614G, P681H	
Hpl 15	0.21%	C23271A, T23344C, A23403G, C23604A	A570D, Syn, D614G, P681H	
Hpl 16	0.20%	C23271A, T23287C, A23403G, C23604A	A570D, Syn, D614G, P681H	
Hpl 17	0.20%	C23271A, A23403G, A23419G, C23604A	A570D, D614G, Syn, P681H	
Hpl 18	0.19%	C23271A, A23403G, T23466C, C23604A	A570D, D614G, V635A, P681H	
Hpl 19	0.19%	C23271A, A23403G, T23427C, C23604A	A570D, D614G, V622A, P681H	
Hpl 20	0.19%	C23271A, T23374C, A23403G, C23604A	A570D, Syn, D614G, P681H	

[a]The genomic region covered by amplicons A1 to A6 of the S-coding region is: A1: nucleotides 21,448 to 21,841; A2: nucleotides 21,727 to 21,217; A3: nucleotides 22,111 to 22,515; A4: nucleotides 22,487 to 22,882;

A5: nucleotides 22,827 to 23,268; A6: nucleotides 23,259 to 23,645. Residue numbering according to NCBI reference sequence: NC_045512.2.

[b]The SARS-CoV-2 genome residue numbering is according to the NCBI reference sequence: NC_045512.2. Those haplotypes which do not present any variation respect the reference sequence are called Wild type.

[c]Amino acid residues (single-letter code) are numbered from N- to C- terminus of each protein (ORF1b or S). Syn: synonymous mutation. All substitutions were located in S except those indicated with ORF1b. For amplicon A1, all haplotypes included deletion 21,765-21,770 that gives rise to deletion of amino acids H69 and V70. For amplicon A2 all haplotypes included deletion 21,765-21,770 that gives rise to deletion of amino acids H69, V70, and 21,992-21,994 that gives rise to deletion of amino acid Y144.

[d]Haplotypes with amino acid substitutions or deletions characteristic of a different variant that the consensus variant.

[e]For Hpl 40, the V70T amino acid is due to the combination of deletion 21,765-21,770 and mutation T21,771C; and the deleted amino acids are I68 and H69.

Table S5. Haplotypes detected in amplicons A1 to A6 of S – coding region in sample from patient Pt455[a].

Spike A1

Haplotype number	Frequency	Nucleotide mutation[b]	Amino acid substitutions[c]	Nucleotide deletions[b]	Deleted amino acids[c]
Hpl 0	99.09%				
Hpl 1	0.24%	A21779G	T73A		
Hpl 2	0.23%	T21477C	Syn	21765-21770	H69Δ, V70Δ
Hpl 3	0.23%	T21594C	V11A		
Hpl 4	0.21%	T21755C	F65L		

Spike A2

Haplotype number	Frequency	Nucleotide mutation[b]	Amino acid substitutions[c]	Nucleotide deletions[b]	Deleted amino acids[c]
Hpl 0	92.59%				
Hpl 1	0.38%	T21773C	S71P		
Hpl 2	0.36%	T21755C	F65L		
Hpl 3	0.36%	T21737C	F59L		
Hpl 4	0.31%	A22102G	Syn		H69Δ, V70Δ, Y144Δ
Hpl 5	0.29%	T21733C	Syn		
Hpl 6	0.27%	T21831C	V90A		
Hpl 7	0.26%	A22025G	S155G		
Hpl 8	0.26%	A22107G	K182R		
Hpl 9	0.25%	T21854C	S98P		
Hpl 10[d]	0.25%	T21771C	V70T		I68Δ, H69Δ, Y144Δ
Hpl 11	0.25%	T21756C	F65S		
Hpl 12	0.24%	A22106G	K182E		
Hpl 13	0.24%	T21990C	V143A		
Hpl 14	0.22%	T21908C	S116P		
Hpl 15	0.22%	A22108G	Syn	21765-21770, 21992-22012	
Hpl 16[e]	0.22%	T21810C	V83A		
Hpl 17	0.21%	T21797C	F79L		
Hpl 18	0.21%	T21747C	V62A		
Hpl 19	0.21%	A22101G	E180G		
Hpl 20	0.21%	T21841C	Syn		
Hpl 21	0.20%	T21842C	S94P		H69Δ, V70Δ, Y144Δ
Hpl 22	0.20%	A22036G	Syn		
Hpl 23	0.20%	A21868G	Syn		
Hpl 24	0.20%	T21735C	F58S		
Hpl 25	0.20%	A22029G	E156G		
Hpl 26	0.20%	A22095G	D178G		
Hpl 27	0.20%	T21808C	Syn		
Hpl 28	0.20%	A21900G	K113R		
Hpl 29	0.19%	A21779G	T73A		
Hpl 30	0.19%	T21889C	Syn		
Hpl 31	0.19%	T22074C	V171A		

Spike A3

Haplotype number	Frequency	Nucleotide mutation[b]	Amino acid substitutions[c]	Nucleotide deletions[b]	Deleted amino acids[c]
Hpl 0	70.40%	Wild type			
Hpl 1	25.15%	C22450T	Syn		
Hpl 2	1.03%	C22187T	P209S		
Hpl 3	0.24%	T22209C	L216P		
Hpl 4	0.23%	T22228C	Syn		
Hpl 5	0.22%	A22411G	Syn		
Hpl 6	0.20%	A22412G	T284A		
Hpl 7	0.19%	A22496G	I312V		
Hpl 8	0.18%	A22443G	D294G		
Hpl 9	0.18%	T22478C	F306L		
Hpl 10	0.17%	T22219C	Syn		
Hpl 11	0.17%	T22384C	Syn		
Hpl 12	0.17%	A22337G	T259A		
Hpl 13	0.16%	A22492G	Syn		
Hpl 14	0.16%	T22274C	F238L		
Hpl 15	0.16%	A22457G	T299A		
Hpl 16	0.16%	T22207C	Syn		
Hpl 17	0.15%	A22455G	E298G		
Hpl 18	0.15%	A22310G	T250A		
Hpl 19	0.15%	T22213C	Syn		
Hpl 20	0.14%	A22234G	Syn		
Hpl 21	0.13%	T22291C	Syn		
Hpl 22	0.12%	T22142C	F194L		

Spike A4

Haplotype number	Frequency	Nucleotide mutation[b]	Amino acid substitutions[c]	Nucleotide deletions[b]	Deleted amino acids[c]
Hpl 0	93.56%	Wild type			
Hpl 1	1.60%	T22690C	Syn		
Hpl 2	0.51%	G22487C	E309Q		
Hpl 3	0.37%	A22780G	Syn		
Hpl 4	0.26%	A22776G	D405G		
Hpl 5	0.25%	T22736C	F392L		
Hpl 6	0.25%	A22623G	N354S		
Hpl 7	0.25%	A22629G	K356R		
Hpl 8	0.24%	A22771G	Syn		
Hpl 9	0.23%	A22786G	Syn		
Hpl 10	0.23%	A22812G	K417R		
Hpl 11	0.22%	A22810G	Syn		
Hpl 12	0.22%	T22514C	F318L		
Hpl 13	0.21%	A22852G	Syn		
Hpl 14	0.21%	T22746C	V395A		
Hpl 15	0.20%	T22709C	S383P		
Hpl 16	0.20%	A22582G	Syn		

Hpl 17	0.20%	T22565C	Syn		
Hpl 18	0.20%	A22519G	Syn		
Hpl 19	0.20%	A22753G	Syn		
Hpl 20	0.20%	A22628G	K356E		
Hpl 21	0.19%	A22878G	N439S		

Spike A5

Haplotype number	Frequency	Nucleotide mutation[b]	Amino acid substitutions[c]	Nucleotide deletions[b]	Deleted amino acids[c]
Hpl 0	83.72%	A23063T	N501Y		
Hpl 1	0.34%	A23063T, A23265G	N501Y, D568G		
Hpl 2	0.33%	T22874C, A23063T	S438P, N501Y		
Hpl 3	0.30%	A23063T, A23263G	N501Y, Syn		
Hpl 4	0.30%	T22951C, A23063T	Syn, N501Y		
Hpl 5	0.28%	T23030C, A23063T	F490L, N501Y		
Hpl 6	0.27%	T22944C, A23063T	L461P, N501Y		
Hpl 7	0.27%	T23050C, A23063T	Syn, N501Y		
Hpl 8	0.26%	A23063T, T23100C	N501Y, L513P		
Hpl 9	0.26%	A23063T, A23223G	N501Y, E554G		
Hpl 10	0.26%	A22958G, A23063T	R466G, N501Y		
Hpl 11	0.25%	T22876C, A23063T	Syn, N501Y		
Hpl 12	0.25%	A23063T, T23104C	N501Y, Syn		
Hpl 13	0.25%	T22888C, A23063T	Syn, N501Y		
Hpl 14	0.25%	A23063T, A23122G	N501Y, Syn		
Hpl 15	0.25%	T22942C, A23063T	Syn, N501Y		
Hpl 16	0.25%	A23063T, A23140G	N501Y, Syn		
Hpl 17	0.24%	A22962G, A23063T	D467G, N501Y		
Hpl 18	0.24%	A22960G, A23063T	Syn, N501Y		
Hpl 19	0.24%	A23063T, T23245C	N501Y, Syn		
Hpl 20	0.23%	T22937C, A23063T	S459P, N501Y		
Hpl 21	0.23%	A23063T, A23235G	N501Y, K558R		
Hpl 22	0.23%	A22948G, A23063T	Syn, N501Y		
Hpl 23	0.23%	T22999C, A23063T	Syn, N501Y		

Hpl 24	0.23%	A22956G, A23063T	E465G, N501Y
Hpl 25	0.22%	T23010C, A23063T	V483A, N501Y
Hpl 26	0.22%	T22896C, A23063T	V445A, N501Y
Hpl 27	0.22%	T22873C, A23063T	Syn, N501Y
Hpl 28	0.22%	A23063T, A23209G	N501Y, Syn
Hpl 29	0.22%	A23063T, T23178C	N501Y, V539A
Hpl 30	0.22%	A22935G, A23063T	K458R, N501Y
Hpl 31	0.22%	A23063T, T23225C	N501Y, S555P
Hpl 32	0.22%	A23063T, T23150C	N501Y, S530P
Hpl 33	0.22%	A23063T, A23201G	N501Y, T547A
Hpl 34	0.21%	A23063T, T23112C	N501Y, L517P
Hpl 35	0.21%	A23063T, T23214C	N501Y, V551A
Hpl 36	0.21%	T22882C, A23063T	Syn, N501Y
Hpl 37	0.21%	T23017C, A23063T	Syn, N501Y
Hpl 38	0.21%	A23014G, A23063T	Syn, N501Y
Hpl 39	0.21%	A23063T, A23261G	N501Y, R567G
Hpl 40	0.21%	A23063T, A23169G	N501Y, N536S
Hpl 41	0.21%	A23063T, A23110G	N501Y, Syn
Hpl 42	0.20%	A23063T, A23148G	N501Y, K529R
Hpl 43	0.20%	A22996G, A23063T	Syn, N501Y
Hpl 44	0.20%	A23063T, A23251G	N501Y, Syn
Hpl 45	0.20%	C22998T, A23063T	P479L, N501Y
Hpl 46	0.20%	A23063T, T23247C	N501Y, F562S
Hpl 47	0.20%	A23063T, T23189C	N501Y, F543L
Hpl 48	0.20%	A22934G, A23063T	K458E, N501Y
Hpl 49	0.20%	A23063T, A23125G	N501Y, Syn
Hpl 50	0.20%	T22889C, A23063T	S443P, N501Y
Hpl 51	0.20%	T22917C, A23063T	L452P, N501Y
Hpl 52	0.20%	T23026C, A23063T	Syn, N501Y

Hpl 53	0.20%	A23063T, A23203G	N501Y, Syn		
Hpl 54	0.19%	A23056G, A23063T	Syn, N501Y		
Hpl 55	0.19%	A23063T, A23229G	N501Y, N556S		
Hpl 56	0.19%	A23063T, T23102C	N501Y, S514P		
Hpl 57	0.19%	A23063T, T23241C	N501Y, L560P		
Hpl 58	0.19%	T23035C, A23063T	Syn, N501Y		
Hpl 59	0.19%	A23063T, T23074C	N501Y, Syn		
Hpl 60	0.19%	T23032C, A23063T	Syn, N501Y		
Hpl 61	0.19%	A23063T, A23267G	N501Y, Syn		
Hpl 62	0.19%	A23063T, A23180G	N501Y, N540D		
Hpl 63	0.18%	A23063T, A23087G	N501Y, R509G		
Hpl 64	0.18%	T23005C, A23063T	Syn, N501Y		
Hpl 65	0.18%	A23063T, T23176C	N501Y, Syn		
Hpl 66	0.18%	T23008C, A23063T	Syn, N501Y		
Hpl 67	0.18%	A23063T, A23168G	N501Y, N536D		
Hpl 68	0.18%	T22990C, A23063T	Syn, N501Y		
Hpl 69	0.18%	A23063T, A23207G	N501Y, T549A		
Hpl 70	0.18%	A23063T, A23089G	N501Y, Syn		
Hpl 71	0.18%	A23041G, A23063T	Syn, N501Y		
Hpl 72	0.18%	A23063T, A23118G	N501Y, H519R		
Hpl 73	0.17%	A23063T, T23163C	N501Y, V534A		
Hpl 74	0.17%	T22884C, A23063T	L441P, N501Y		
Hpl 75	0.15%	A23063T, T23182C	N501Y, Syn		

Spike A6

Haplotype number	Frequency	Nucleotide mutation[b]	Amino acid substitutions[c]	Nucleotide deletions[b]	Deleted amino acids[c]
Hpl 0	88.48%	C23271A, A23403G, C23604A	A570D, D614G, P681H		
Hpl 1	0.50%	C23271A, A23403G, A23544G, C23604A	A570D, D614G, E661G, P681H		

Hpl 2	0.32%	C23271A, T23332C, A23403G, C23604A	A570D, Syn, D614G, P681H
Hpl 3	0.30%	C23271A, T23374C, A23403G, C23604A	A570D, Syn, D614G, P681H
Hpl 4	0.29%	C23271A, A23403G, A23566G, C23604A	A570D, D614G, Syn, P681H
Hpl 5	0.29%	C23271A, T23352C, A23403G, C23604A	A570D, V597A, D614G, P681H
Hpl 6	0.28%	C23271A, T23346C, A23403G, C23604A	A570D, V595A, D614G, P681H
Hpl 7	0.28%	C23271A, T23391C, A23403G, C23604A	A570D, V610A, D614G, P681H
Hpl 8	0.28%	C23271A, A23403G, A23524G, C23604A	A570D, D614G, Syn, P681H
Hpl 9	0.27%	C23271A, T23394C, A23403G, C23604A	A570D, L611P, D614G, P681H
Hpl 10	0.27%	C23271A, A23403G, A23588G, C23604A	A570D, D614G, T676A, P681H
Hpl 11	0.26%	C23271A, A23403G, A23419G, C23604A	A570D, D614G, Syn, P681H
Hpl 12	0.25%	C23271A, A23366G, A23403G, C23604A	A570D, T602A, D614G, P681H
Hpl 13	0.25%	C23271A, A23403G, A23594G, C23604A	A570D, D614G, T678A, P681H
Hpl 14	0.25%	C23271A, A23403G, A23550G, C23604A	A570D, D614G, D663G, P681H
Hpl 15	0.25%	C23271A, A23403G, A23440G, C23604A	A570D, D614G, Syn, P681H
Hpl 16	0.25%	C23271A, A23403G,	A570D, D614G,

		A23495G, C23604A	T645A, P681H
Hpl 17	0.24%	C23271A, T23350C, A23403G, C23604A	A570D, Syn, D614G, P681H
Hpl 18	0.23%	C23271A, T23333C, A23403G, C23604A	A570D, S591P, D614G, P681H
Hpl 19	0.23%	C23271A, A23403G, A23503G, C23604A	A570D, D614G, Syn, P681H
Hpl 20	0.23%	C23271A, A23403G, T23421C, C23604A	A570D, D614G, V620A, P681H
Hpl 21	0.23%	C23271A, A23403G, T23406C, C23604A	A570D, D614G, V615A, P681H
Hpl 22	0.22%	C23271A, T23335C, A23403G, C23604A	A570D, Syn, D614G, P681H
Hpl 23	0.22%	C23271A, T23389C, A23403G, C23604A	A570D, Syn, D614G, P681H
Hpl 24	0.22%	C23271A, A23403G, A23476G, C23604A	A570D, D614G, Syn, P681H
Hpl 25	0.22%	C23271A, A23403G, T23479C, C23604A	A570D, D614G, Syn, P681H
Hpl 26	0.22%	C23271A, T23289C, A23403G, C23604A	A570D, V576A, D614G, P681H
Hpl 27	0.21%	C23271A, A23403G, T23452C, C23604A	A570D, D614G, Syn, P681H
Hpl 28	0.21%	C23271A, A23326G, A23403G, C23604A	A570D, Syn, D614G, P681H
Hpl 29	0.21%	C23271A, T23344C, A23403G, C23604A	A570D, Syn, D614G, P681H
Hpl 30	0.20%	C23271A, T23392C, A23403G, C23604A	A570D, Syn, D614G, P681H

Hpl 31	0.20%	C23271A, A23403G, T23433C, C23604A	A570D, D614G, I624T, P681H	
Hpl 32	0.20%	C23271A, A23403G, A23497G, C23604A	A570D, D614G, Syn, P681H	
Hpl 33	0.20%	C23271A, A23403G, T23489C, C23604A	A570D, D614G, F643L, P681H	
Hpl 34	0.20%	C23271A, A23303G, A23403G, C23604A	A570D, T581A, D614G, P681H	
Hpl 35	0.20%	C23271A, A23403G, T23466C, C23604A	A570D, D614G, V635A, P681H	
Hpl 36	0.20%	C23271A, A23403G, T23404C, C23604A	A570D, D614G, Syn, P681H	
Hpl 37	0.20%	C23271A, A23400G, A23403G, C23604A	A570D, Q613R, D614G, P681H	
Hpl 38	0.20%	C23271A, T23296C, A23403G, C23604A	A570D, Syn, D614G, P681H	
Hpl 39	0.20%	C23271A, A23403G, A23414G, C23604A	A570D, D614G, T618A, P681H	
Hpl 40	0.20%	C23271A, A23310G, A23403G, C23604A	A570D, E583G, D614G, P681H	
Hpl 41	0.19%	C23271A, A23319G, A23403G, C23604A	A570D, D586G, D614G, P681H	
Hpl 42	0.19%	C23271A, A23403G, T23487C, C23604A	A570D, D614G, V642A, P681H	
Hpl 43	0.19%	C23271A, T23287C, A23403G, C23604A	A570D, Syn, D614G, P681H	
Hpl 44	0.19%	C23271A, A23403G, T23411C, C23604A	A570D, D614G, C617R, P681H	
Hpl 45[e]	0.19%	C23271A, A23403G, C23604G,	A570D, D614G, P681R,	

Hpl 46	0.18%	C23271A, A23403G, T23529C, C23604A	A570D, D614G, V656A, P681H
Hpl 47	0.18%	C23271A, A23403G, C23604A, A23623G	A570D, D614G, P681H, Syn
Hpl 48	0.18%	C23271A, T23307C, A23403G, C23604A	A570D, L582P, D614G, P681H
Hpl 49	0.18%	C23271A, T23293C, A23403G, C23604A	A570D, Syn, D614G, P681H
Hpl 50	0.18%	C23271A, T23272C, A23403G, C23604A	A570D, Syn, D614G, P681H

[a]The genomic region covered by amplicons A1 to A6 of the S-coding region is: A1: nucleotides 21,448 to 21,841; A2: nucleotides 21,727 to 21,217; A3: nucleotides 22,111 to 22,515; A4: nucleotides 22,487 to 22,882; A5: nucleotides 22,827 to 23,268; A6: nucleotides 23,259 to 23,645. Residue numbering according to NCBI reference sequence: NC_045512.2

[b]The SARS-CoV-2 genome residue numbering is according to the NCBI reference sequence: NC_045512.2. Those haplotypes which do not present any variation respect the reference sequence are called Wild type.

[c]Amino acid residues (single-letter code) are numbered from N- to C- terminus of each protein (ORF1b or S). Syn: synonymous mutation. All substitutions were located in S except those indicated with ORF1b. For amplicons A1 all haplotypes included deletion 21,765-21,770 that gives rise to deletion of amino acids H69 and V70. For amplicon A2 all haplotypes included deletion 21,765-21,770 that gives rise to deletion of amino acids H69, V70, and 21,992-21,994 that gives rise to deletion of amino acid Y144.

[d]For Hpl 10, the V70T amino acid is due to the combination of deletion 21,765-21,770 and mutation T21,771C; and the deleted amino acids are I68 and H69.

[e]Haplotypes with amino acid substitutions or deletions characteristic of a different variant that the consensus variant.

Table S6. Haplotypes detected amplicons in A1 to A6 of S – coding region in sample from patient Pt456[a].

Spike A1

Haplotype number	Frequency	Nucleotide mutation[b]	Amino acid substitutions[c]	Nucleotide deletions[b]	Deleted amino acids[c]
Hpl 0	92.54%				
Hpl 1	0.43%	T21722C	Syn		
Hpl 2	0.34%	T21737C	F59L		
Hpl 3	0.28%	A21489G	Syn		
Hpl 4	0.26%	A21599G	S13G		
Hpl 5	0.25%	T21479C	ORF1b:L2671P		
Hpl 6	0.25%	A21779G	T73A		
Hpl 7	0.24%	T21477C	Syn		
Hpl 8	0.23%	T21594C	V11A		
Hpl 9	0.23%	T21539C	ORF1b:V2691A		
Hpl 10	0.22%	A21720G	D53G		
Hpl 11	0.22%	T21596C	S12P		
Hpl 12	0.21%	T21655C	Syn	21765-21770	H69Δ, V70Δ
Hpl 13	0.21%	T21475C	ORF1b:S2670P		
Hpl 14	0.21%	T21755C	F65L		
Hpl 15	0.21%	T21497C	ORF1b:L2677P		
Hpl 16	0.21%	A21685G	Syn		
Hpl 17	0.20%	T21579C	V6A		
Hpl 18	0.20%	T21570C	V3A		
Hpl 19	0.20%	A21794G	R78G		
Hpl 20	0.20%	T21522C	Syn		
Hpl 21	0.20%	T21540C	Syn		
Hpl 22	0.19%	T21733C	Syn		
Hpl 23	0.19%	A21505G	ORF1b:R2680G		
Hpl 24	0.19%	T21524C	ORF1b:V2686A		
Hpl 25	0.19%			21764-21769	I68Δ, H69Δ
Hpl 26	0.18%	T21566C	F2L		
Hpl 27	0.18%	T21702C	V47A		
Hpl 28	0.18%	A21804G	N81S		
Hpl 29	0.16%	T21797C	F79L		
Hpl 30	0.16%	T21576C	L5P		
Hpl 31	0.15%	T21598C	Syn	21765-21770	H69Δ, V70Δ
Hpl 32	0.15%	A21730G	Syn		
Hpl 33	0.15%	A21792G	K77R		
Hpl 34	0.14%	T21649C	Syn		
Hpl 35	0.13%	T21591C	L10P		
Hpl 36	0.11%	T21610C	Syn		

Spike A2

Haplotype number	Frequency	Nucleotide mutation[b]	Amino acid substitutions[c]	Nucleotide deletions[b]	Deleted amino acids[c]

Hpl 0	89.85%			21765-21770, 21992-21994	H69Δ, V70Δ, Y144Δ
Hpl 1	0.41%	T21773C	S71P		
Hpl 2	0.39%	T21737C	F59L		
Hpl 3	0.34%	A22102G	Syn		
Hpl 4	0.33%	T21733C	Syn		
Hpl 5	0.33%	T21755C	F65L		
Hpl 6	0.33%	A22025G	S155G		
Hpl 7	0.32%	T21797C	F79L		
Hpl 8	0.30%	A22095G	D178G		
Hpl 9	0.30%	T22084C	Syn		
Hpl 10	0.27%	T21841C	Syn		
Hpl 11	0.26%	A22029G	E156G		
Hpl 12	0.26%	A22101G	E180G		
Hpl 13[d]	0.25%	T21810C	V83A		
Hpl 14	0.24%	A22108G	Syn		
Hpl 15	0.24%	T21831C	V90A		
Hpl 16	0.23%	A21852G	K97R		
Hpl 17	0.23%	T21808C	Syn		
Hpl 18	0.23%	T21747C	V62A		
Hpl 19	0.23%	T21842C	S94P		
Hpl 20[e]	0.23%	T21771C	V70T		I68Δ, H69Δ, Y144Δ
Hpl 21	0.22%	T21908C	S116P		H69Δ, V70Δ Y144Δ
Hpl 22	0.22%	T21735C	F58S		
Hpl 23[d]	0.22%	A22034G	R158G		
Hpl 24	0.22%			21764-21769, 21992-21994	I68Δ, H69Δ, Y144Δ
Hpl 25[d]	0.22%	T22031C	F157L	21765-21770, 21992-21994	H69Δ, V70Δ, Y144Δ
Hpl 26	0.22%	A22107G	K182R		
Hpl 27	0.21%	A21851G	K97E		
Hpl 28	0.21%	A22008G	N149S		
Hpl 29	0.20%	T21829C	Syn		
Hpl 30	0.20%	T21856C	Syn		
Hpl 31	0.20%	A22106G	K182E		
Hpl 32	0.20%	T21756C	F65S		
Hpl 33	0.20%	A22105G	Syn		
Hpl 34	0.20%	T21728C	Syn		
Hpl 35	0.19%	A21900G	K113R		
Hpl 36	0.19%	A22056G	N165S		
Hpl 37	0.19%	T21889C	Syn		
Hpl 38	0.19%	A22013G	S151G		
Hpl 39	0.19%	A21902G	T114A		
Hpl 40	0.18%	T22076C	S172P		
Hpl 41	0.17%	T22063C	Syn		
Hpl 42	0.17%	T21751C	Syn		

Spike A3

Haplotype number	Frequency	Nucleotide mutation[b]	Amino acid substitutions[c]	Nucleotide deletions[b]	Deleted amino acids[c]
Hpl 0	89.68%	Wild type			
Hpl 1	0.48%	T22384C	Syn		
Hpl 2	0.30%	T22291C	Syn		
Hpl 3	0.30%	A22412G	T284A		
Hpl 4	0.30%	T22510C	Syn		
Hpl 5	0.29%	A22492G	Syn		
Hpl 6	0.29%	T22209C	L216P		
Hpl 7	0.25%	T22371C	L270P		
Hpl 8	0.25%	T22219C	Syn		
Hpl 9	0.25%	A22457G	T299A		
Hpl 10	0.25%	T22514C	Syn		
Hpl 11	0.23%	A22351G	Syn		
Hpl 12	0.23%	A22496G	I312V		
Hpl 13	0.22%	A22443G	D294G		
Hpl 14	0.22%	T22228C	Syn		
Hpl 15	0.22%	T22177C	Syn		
Hpl 16	0.21%	T22478C	F306L		
Hpl 17	0.21%	A22461G	K300R		
Hpl 18	0.21%	A22337G	T259A		
Hpl 19	0.21%	T22324C	Syn		
Hpl 20	0.21%	T22274C	F238L		
Hpl 21	0.21%	A22505G	T315A		
Hpl 22	0.21%	A22234G	Syn		
Hpl 23	0.21%	A22301G	S247G		
Hpl 24	0.21%	T22207C	Syn		
Hpl 25	0.21%	A22455G	E298G		
Hpl 26	0.20%	A22411G	Syn		
Hpl 27	0.20%	A22495G	Syn		
Hpl 28	0.20%	A22474G	Syn		
Hpl 29	0.20%	A22431G	D290G		
Hpl 30	0.20%	T22449C	L296P		
Hpl 31	0.20%	T22137C	F192S		
Hpl 32	0.20%	A22456G	Syn		
Hpl 33	0.20%	T22282C	Syn		
Hpl 34	0.19%	T22475C	S305P		
Hpl 35	0.19%	A22310G	T250A		
Hpl 36	0.19%	T22386C	F275S		
Hpl 37	0.19%	T22213C	Syn		
Hpl 38	0.19%	T22287C	L242P		
Hpl 39	0.19%	A22375G	Syn		
Hpl 40	0.19%	T22447C	Syn		
Hpl 41	0.18%	T22507C	Syn		
Hpl 42	0.18%	A22491G	K310R		
Hpl 43	0.18%	T22321C	Syn		

Hpl 44	0.18%	T22327C	Syn		
Hpl 45	0.17%	T22426C	Syn		
Hpl 46	0.17%	A22407G	N282S		
Hpl 47	0.17%	T22354C	Syn		

Spike A4

Haplotype number	Frequency	Nucleotide mutation[b]	Amino acid substitutions[c]	Nucleotide deletions[b]	Deleted amino acids[c]
Hpl 0	89.33%	Wild type			
Hpl 1	1.61%	G22487C	E309Q		
Hpl 2	1.14%	T22690C	Syn		
Hpl 3	0.35%	A22780G	Syn		
Hpl 4	0.32%	T22736C	F392L		
Hpl 5	0.30%	T22510C	Syn		
Hpl 6	0.29%	A22812G	K417R		
Hpl 7	0.26%	T22507C	Syn		
Hpl 8	0.26%	T22514C	F318L		
Hpl 9	0.24%	A22544G	R328G		
Hpl 10	0.24%	A22786G	Syn		
Hpl 11	0.24%	A22810G	Syn		
Hpl 12	0.24%	A22492G	Syn		
Hpl 13	0.23%	A22623G	N354S		
Hpl 14	0.23%	A22878G	N439S		
Hpl 15	0.23%	T22501C	Syn		
Hpl 16	0.22%	A22852G	Syn		
Hpl 17	0.22%	T22565C	Syn		
Hpl 18	0.21%	T22856C	C432R		
Hpl 19	0.21%	T22579C	Syn		
Hpl 20	0.21%	T22711C	Syn		
Hpl 21	0.20%	A22842G	D427G		
Hpl 22	0.20%	A22771G	Syn		
Hpl 23	0.20%	T22573C	Syn		
Hpl 24	0.20%	T22741C	Syn		
Hpl 25	0.20%	A22629G	K356R		
Hpl 26	0.19%	A22776G	D405G		
Hpl 27	0.19%	T22552C	Syn		
Hpl 28	0.19%	T22709C	S383P		
Hpl 29	0.19%	A22582G	Syn		
Hpl 30	0.19%	T22611C	V350A		
Hpl 31	0.18%	A22864G	I434M		
Hpl 32	0.18%	A22641G	N360S		
Hpl 33	0.18%	A22769G	R403G		
Hpl 34	0.18%	T22707C	V382A		
Hpl 35	0.17%	A22534G	Syn		
Hpl 36	0.16%	T22547C	F329L		
Hpl 37	0.15%	T22647C	V362A		
Hpl 38	0.14%	T22774C	Syn		

Hpl 39	0.13%	T22537C	Syn		

Spike A5

Haplotype number	Frequency	Nucleotide mutation[b]	Amino acid substitutions[c]	Nucleotide deletions[b]	Deleted amino acids[c]
Hpl 0	80.25%	A23063T	N501Y		
Hpl 1	0.37%	T22874C, A23063T	S438P, N501Y		
Hpl 2	0.33%	A23063T, A23148G	N501Y, K529R		
Hpl 3	0.32%	A23063T, A23265G	N501Y, D568G		
Hpl 4	0.32%	A23063T, A23263G	N501Y, Syn		
Hpl 5	0.31%	T23030C, A23063T	F490L, N501Y		
Hpl 6	0.31%	T22873C, A23063T	Syn, N501Y		
Hpl 7	0.29%	A23063T, T23245C	N501Y, Syn		
Hpl 8	0.28%	A22960G, A23063T	Syn, N501Y		
Hpl 9	0.28%	T22942C, A23063T	Syn, N501Y		
Hpl 10	0.28%	A23063T, T23100C	N501Y, L513P		
Hpl 11	0.28%	A23063T, A23140G	N501Y, Syn		
Hpl 12	0.28%	Wild type			
Hpl 13	0.28%	T22944C, A23063T	L461P, N501Y		
Hpl 14	0.27%	A22935G, A23063T	K458R, N501Y		
Hpl 15	0.26%	T22937C, A23063T	S459P, N501Y		
Hpl 16	0.26%	A22958G, A23063T	R466G, N501Y		
Hpl 17	0.26%	A23063T, A23223G	N501Y, E554G		
Hpl 18	0.26%	A23063T, T23214C	N501Y, V551A		
Hpl 19	0.25%	T22876C, A23063T	Syn, N501Y		
Hpl 20	0.25%	T22951C, A23063T	Syn, N501Y		
Hpl 21	0.25%	A23063T, A23110G	N501Y, Syn		
Hpl 22	0.25%	A22956G, A23063T	E465G, N501Y		
Hpl 23	0.24%	A23063T, T23150C	N501Y, S530P		
Hpl 24	0.24%	T22999C, A23063T	Syn, N501Y		
Hpl 25	0.24%	A23063T, T23104C	N501Y, Syn		
Hpl 26	0.24%	A23063T, A23207G	N501Y, T549A		

Hpl 27	0.24%	A23063T, T23178C	N501Y, V539A
Hpl 28	0.24%	A23063T, A23261G	N501Y, R567G
Hpl 29	0.24%	A23063T, T23112C	N501Y, L517P
Hpl 30	0.24%	A22962G, A23063T	D467G, N501Y
Hpl 31	0.23%	A23063T, A23169G	N501Y, N536S
Hpl 32	0.23%	A23063T, A23122G	N501Y, Syn
Hpl 33	0.23%	T23017C, A23063T	Syn, N501Y
Hpl 34	0.23%	T22888C, A23063T	Syn, N501Y
Hpl 35	0.23%	A23041G, A23063T	Syn, N501Y
Hpl 36	0.23%	A23063T, A23092G	N501Y, Syn
Hpl 37	0.23%	T23026C, A23063T	Syn, N501Y
Hpl 38	0.22%	T23042C, A23063T	S494P, N501Y
Hpl 39	0.22%	T22917C, A23063T	L452P, N501Y
Hpl 40	0.22%	A23063T, A23203G	N501Y, Syn
Hpl 41	0.21%	A23063T, T23068C	N501Y, Syn
Hpl 42	0.21%	T23050C, A23063T	Syn, N501Y
Hpl 43	0.21%	A23063T, A23168G	N501Y, N536D
Hpl 44	0.21%	A23063T, A23209G	N501Y, Syn
Hpl 45	0.21%	T23008C, A23063T	Syn, N501Y
Hpl 46	0.21%	T23032C, A23063T	Syn, N501Y
Hpl 47	0.21%	A23063T, A23118G	N501Y, H519R
Hpl 48	0.21%	A23063T, T23215C	N501Y, Syn
Hpl 49	0.21%	A23063T, A23087G	N501Y, R509G
Hpl 50	0.21%	T22896C, A23063T	V445A, N501Y
Hpl 51	0.21%	A23063T, A23089G	N501Y, Syn
Hpl 52	0.21%	A23063T, A23201G	N501Y, T547A
Hpl 53	0.21%	A23063T, A23251G	N501Y, Syn
Hpl 54	0.20%	A23063T, T23225C	N501Y, S555P
Hpl 55	0.20%	A23063T, A23129G	N501Y, T523A

Hpl 56	0.20%	A23063T, A23267G	N501Y, Syn
Hpl 57	0.20%	T22882C, A23063T	Syn, N501Y
Hpl 58	0.20%	A23014G, A23063T	Syn, N501Y
Hpl 59	0.20%	T22889C, A23063T	S443P, N501Y
Hpl 60	0.20%	A23063T, T23246C	N501Y, F562L
Hpl 61	0.20%	A22948G, A23063T	Syn, N501Y
Hpl 62	0.20%	A23063T, A23233G	N501Y, Syn
Hpl 63	0.20%	A23063T, T23074C	N501Y, Syn
Hpl 64	0.20%	T23035C, A23063T	Syn, N501Y
Hpl 65	0.19%	A23063T, A23173G	N501Y, Syn
Hpl 66	0.19%	A23063T, T23133C	N501Y, V524A
Hpl 67	0.19%	T22990C, A23063T	Syn, N501Y
Hpl 68	0.19%	A23063T, A23229G	N501Y, N556S
Hpl 69	0.19%	T22928C, A23063T	F456L, N501Y
Hpl 70	0.19%	T23051C, A23063T	F497L, N501Y
Hpl 71	0.19%	A23063T, T23189C	N501Y, F543L
Hpl 72	0.19%	A22996G, A23063T	Syn, N501Y
Hpl 73	0.18%	A23063T, T23176C	N501Y, Syn
Hpl 74	0.18%	T23010C, A23063T	V483A, N501Y
Hpl 75	0.18%	A23063T, A23250G	N501Y, Q563R
Hpl 76	0.18%	A23063T, A23180G	N501Y, N540D
Hpl 77	0.18%	A23063T, A23109G	N501Y, E516G
Hpl 78	0.18%	A23063T, A23095G	N501Y, Syn
Hpl 79	0.18%	T23062C, A23063T	Syn, N501Y
Hpl 80	0.17%	A23063T, T23217C	N501Y, L552P
Hpl 81	0.17%	A23063T, T23102C	N501Y, S514P
Hpl 82	0.17%	T22939C, A23063T	Syn, N501Y
Hpl 83	0.17%	A23063T, T23163C	N501Y, V534A
Hpl 84	0.17%	A23056G, A23063T	Syn, N501Y

Hpl 85	0.16%	A23063T, A23125G	N501Y, Syn		
Hpl 86	0.16%	T23036C, A23063T	Syn, N501Y		
Hpl 87	0.16%	A22994G, A23063T	T478A, N501Y		
Hpl 88	0.16%	A23063T, A23079G	N501Y, Q506R		
Hpl 89	0.14%	A23063T, T23182C	N501Y, Syn		

Spike A6

Haplotype number	Frequency	Nucleotide mutation[b]	Amino acid substitutions[c]	Nucleotide deletions[b]	Deleted amino acids[c]
Hpl 0	80.90%	C23271A, A23403G, C23604A	A570D, D614G, P681H		
Hpl 1	0.70%	C23271A, A23403G, A23419G, C23604A	A570D, D614G, Syn, P681H		
Hpl 2	0.55%	C23271A, A23403G, A23544G, C23604A	A570D, D614G, E661G, P681H		
Hpl 3	0.34%	C23271A, T23394C, A23403G, C23604A	A570D, L611P, D614G, P681H		
Hpl 4	0.29%	C23271A, A23403G, T23421C, C23604A	A570D, D614G, V620A, P681H		
Hpl 5	0.29%	C23271A, T23391C, A23403G, C23604A	A570D, V610A, D614G, P681H		
Hpl 6	0.29%	C23271A, T23350C, A23403G, C23604A	A570D, Syn, D614G, P681H		
Hpl 7	0.28%	C23271A, T23332C, A23403G, C23604A	A570D, Syn, D614G, P681H		
Hpl 8	0.28%	C23271A, T23344C, A23403G, C23604A	A570D, Syn, D614G, P681H		
Hpl 9	0.28%	C23271A, T23374C, A23403G, C23604A	A570D, Syn, D614G, P681H		
Hpl 10	0.27%	C23271A, A23403G, A23495G, C23604A	A570D, D614G, T645A, P681H		
Hpl 11	0.27%	A23403G	D614G		

Hpl 12	0.26%	C23271A,<br>A23366G,<br>A23403G,<br>C23604A	A570D,<br>T602A,<br>D614G,<br>P681H
Hpl 13	0.26%	C23271A,<br>A23403G,<br>A23566G,<br>C23604A	A570D,<br>D614G,<br>Syn,<br>P681H
Hpl 14	0.26%	C23271A,<br>A23403G,<br>A23550G,<br>C23604A	A570D,<br>D614G,<br>D663G,<br>P681H
Hpl 15	0.26%	C23271A,<br>A23403G,<br>T23479C,<br>C23604A	A570D,<br>D614G,<br>Syn,<br>P681H
Hpl 16	0.26%	C23271A,<br>A23403G,<br>A23594G,<br>C23604A	A570D,<br>D614G,<br>T678A,<br>P681H
Hpl 17	0.25%	C23271A,<br>A23403G,<br>T23452C,<br>C23604A	A570D,<br>D614G,<br>Syn,<br>P681H
Hpl 18	0.25%	C23271A,<br>T23289C,<br>A23403G,<br>C23604A	A570D,<br>V576A,<br>D614G,<br>P681H
Hpl 19	0.24%	C23271A,<br>T23335C,<br>A23403G,<br>C23604A	A570D,<br>Syn,<br>D614G,<br>P681H
Hpl 20	0.24%	C23271A,<br>T23307C,<br>A23403G,<br>C23604A	A570D,<br>L582P,<br>D614G,<br>P681H
Hpl 21	0.24%	C23271A,<br>A23403G,<br>T23406C,<br>C23604A	A570D,<br>D614G,<br>V615A,<br>P681H
Hpl 22	0.24%	C23271A,<br>A23403G,<br>A23524G,<br>C23604A	A570D,<br>D614G,<br>Syn,<br>P681H
Hpl 23	0.23%	C23271A,<br>A23403G,<br>T23448C,<br>C23604A	A570D,<br>D614G,<br>L629P,<br>P681H
Hpl 24	0.23%	C23271A,<br>A23403G,<br>C23604A,<br>A23627G	A570D,<br>D614G,<br>P681H,<br>S689G
Hpl 25	0.23%	C23271A,<br>T23287C,<br>A23403G,<br>C23604A	A570D,<br>Syn,<br>D614G,<br>P681H
Hpl 26	0.23%	C23271A,<br>T23333C,<br>A23403G,	A570D,<br>S591P,<br>D614G,

		C23604A	P681H
Hpl 27	0.23%	C23271A,<br>A23403G,<br>A23588G,<br>C23604A	A570D,<br>D614G,<br>T676A,<br>P681H
Hpl 28	0.23%	C23271A,<br>A23303G,<br>A23403G,<br>C23604A	A570D,<br>T581A,<br>D614G,<br>P681H
Hpl 29	0.23%	C23271A,<br>T23346C,<br>A23403G,<br>C23604A	A570D,<br>V595A,<br>D614G,<br>P681H
Hpl 30	0.22%	C23271A,<br>A23403G,<br>A23440G,<br>C23604A	A570D,<br>D614G,<br>Syn,<br>P681H
Hpl 31	0.22%	C23271A,<br>A23403G,<br>A23476G,<br>C23604A	A570D,<br>D614G,<br>Syn,<br>P681H
Hpl 32	0.22%	C23271A,<br>A23403G,<br>A23503G,<br>C23604A	A570D,<br>D614G,<br>Syn,<br>P681H
Hpl 33	0.22%	C23271A,<br>T23296C,<br>A23403G,<br>C23604A	A570D,<br>Syn,<br>D614G,<br>P681H
Hpl 34	0.22%	C23271A,<br>A23403G,<br>T23428C,<br>C23604A	A570D,<br>D614G,<br>Syn,<br>P681H
Hpl 35	0.22%	C23271A,<br>A23403G,<br>A23414G,<br>C23604A	A570D,<br>D614G,<br>T618A,<br>P681H
Hpl 36	0.22%	C23271A,<br>A23403G,<br>T23433C,<br>C23604A	A570D,<br>D614G,<br>I624T,<br>P681H
Hpl 37	0.22%	C23271A,<br>A23403G,<br>T23489C,<br>C23604A	A570D,<br>D614G,<br>F643L,<br>P681H
Hpl 38	0.21%	C23271A,<br>A23403G,<br>T23427C,<br>C23604A	A570D,<br>D614G,<br>V622A,<br>P681H
Hpl 39	0.21%	C23271A,<br>T23392C,<br>A23403G,<br>C23604A	A570D,<br>Syn,<br>D614G,<br>P681H
Hpl 40	0.21%	C23271A,<br>T23272C,<br>A23403G,<br>C23604A	A570D,<br>Syn,<br>D614G,<br>P681H
Hpl 41	0.21%	C23271A,<br>A23403G,	A570D,<br>D614G,

		A23531G,<br>C23604A	N657D,<br>P681H
Hpl 42	0.21%	C23271A,<br>A23403G,<br>T23487C,<br>C23604A	A570D,<br>D614G,<br>V642A,<br>P681H
Hpl 43	0.21%	C23271A,<br>A23403G,<br>T23466C,<br>C23604A	A570D,<br>D614G,<br>V635A,<br>P681H
Hpl 44	0.21%	C23271A,<br>T23293C,<br>A23403G,<br>C23604A	A570D,<br>Syn,<br>D614G,<br>P681H
Hpl 45	0.20%	C23271A,<br>A23310G,<br>A23403G,<br>C23604A	A570D,<br>E583G,<br>D614G,<br>P681H
Hpl 46	0.20%	C23271A,<br>T23352C,<br>A23403G,<br>C23604A	A570D,<br>V597A,<br>D614G,<br>P681H
Hpl 47	0.20%	C23271A,<br>A23400G,<br>A23403G,<br>C23604A	A570D,<br>Q613R,<br>D614G,<br>P681H
Hpl 48	0.20%	C23271A,<br>A23403G,<br>T23548C,<br>C23604A	A570D,<br>D614G,<br>Syn,<br>P681H
Hpl 49	0.20%	C23271A,<br>A23326G,<br>A23403G,<br>C23604A	A570D,<br>Syn,<br>D614G,<br>P681H
Hpl 50	0.20%	C23271A,<br>T23330C,<br>A23403G,<br>C23604A	A570D,<br>C590R,<br>D614G,<br>P681H
Hpl 51	0.20%	C23271A,<br>A23403G,<br>T23411C,<br>C23604A	A570D,<br>D614G,<br>C617R,<br>P681H
Hpl 52	0.19%	C23271A,<br>A23403G,<br>A23592G,<br>C23604A	A570D,<br>D614G,<br>Q677R,<br>P681H
Hpl 53	0.19%	C23271A,<br>A23403G,<br>T23480C,<br>C23604A	A570D,<br>D614G,<br>S640P,<br>P681H
Hpl 54	0.19%	C23271A,<br>A23319G,<br>A23403G,<br>C23604A	A570D,<br>D586G,<br>D614G,<br>P681H
Hpl 55	0.19%	C23271A,<br>A23359G,<br>A23403G,<br>C23604A	A570D,<br>Syn,<br>D614G,<br>P681H

Hpl 56	0.19%	C23271A,<br>T23389C,<br>A23403G,<br>C23604A	A570D,<br>Syn,<br>D614G,<br>P681H
Hpl 57	0.19%	C23271A,<br>T23322C,<br>A23403G,<br>C23604A	A570D,<br>I587T,<br>D614G,<br>P681H
Hpl 58	0.19%	C23271A,<br>T23385C,<br>A23403G,<br>C23604A	A570D,<br>V608A,<br>D614G,<br>P681H
Hpl 59	0.19%	C23271A,<br>A23403G,<br>T23509C,<br>C23604A	A570D,<br>D614G,<br>Syn,<br>P681H
Hpl 60	0.19%	C23271A,<br>A23403G,<br>C23604A,<br>A23618G	A570D,<br>D614G,<br>P681H,<br>S686G
Hpl 61	0.19%	C23271A,<br>A23403G,<br>A23445G,<br>C23604A	A570D,<br>D614G,<br>Q628R,<br>P681H
Hpl 62	0.19%	C23271A,<br>A23403G,<br>A23497G,<br>C23604A	A570D,<br>D614G,<br>Syn,<br>P681H
Hpl 63	0.18%	C23271A,<br>A23403G,<br>T23507C,<br>C23604A	A570D,<br>D614G,<br>C649R,<br>P681H
Hpl 64[d]	0.18%	C23271A,<br>A23403G,<br>C23604G,	A570D,<br>D614G,<br>P681R,
Hpl 65	0.18%	C23271A,<br>T23281C,<br>A23403G,<br>C23604A	A570D,<br>Syn,<br>D614G,<br>P681H
Hpl 66	0.18%	C23271A,<br>T23308C,<br>A23403G,<br>C23604A	A570D,<br>Syn,<br>D614G,<br>P681H
Hpl 67	0.18%	C23271A,<br>A23403G,<br>T23434C,<br>C23604A	A570D,<br>D614G,<br>Syn,<br>P681H
Hpl 68	0.18%	C23271A,<br>A23403G,<br>T23529C,<br>C23604A	A570D,<br>D614G,<br>V656A,<br>P681H
Hpl 69	0.18%	C23271A,<br>A23403G,<br>A23579G,<br>C23604A	A570D,<br>D614G,<br>S673G,<br>P681H
Hpl 70	0.18%	C23271A,<br>A23403G,<br>A23598G,<br>C23604A	A570D,<br>D614G,<br>N679S,<br>P681H

Hpl 71	0.18%	C23271A, A23403G, T23521C, C23604A	A570D, D614G, Syn, P681H
Hpl 72	0.18%	A23263G, C23271A, A23403G, C23604A	Syn, A570D, D614G, P681H
Hpl 73	0.18%	C23271A, A23403G, C23604A, A23623G	A570D, D614G, P681H, Syn
Hpl 74	0.18%	C23271A, A23295G, A23403G, C23604A	A570D, D578G, D614G, P681H
Hpl 75	0.17%	A23265G, C23271A, A23403G, C23604A	D568G, A570D, D614G, P681H
Hpl 76	0.17%	C23271A, A23274G, A23403G, C23604A	A570D, D571G, D614G, P681H
Hpl 77	0.17%	C23271A, A23403G, C23604A, A23614G	A570D, D614G, P681H, Syn
Hpl 78	0.17%	C23271A, A23403G, A23515G, C23604A	A570D, D614G, I651M, P681H
Hpl 79	0.17%	C23271A, T23284C, A23403G, C23604A	A570D, Syn, D614G, P681H
Hpl 80	0.16%	C23271A, A23301G, A23403G, C23604A	A570D, Q580R, D614G, P681H
Hpl 81	0.16%	C23271A, A23403G, A23532G, C23604A	A570D, D614G, N657S, P681H
Hpl 82	0.16%	C23271A, A23403G, T23464C, C23604A	A570D, D614G, Syn, P681H
Hpl 83	0.16%	C23271A, A23382G, A23403G, C23604A	A570D, Q607R, D614G, P681H
Hpl 84	0.15%	C23271A, A23403G, A23409G, C23604A	A570D, D614G, N616S, P681H
Hpl 85	0.15%	C23271A, A23403G, A23586G,	A570D, D614G, Q675R,

		C23604A	P681H	
Hpl 86	0.13%	C23271A,<br>A23403G,<br>A23450G,<br>C23604A	A570D,<br>D614G,<br>T630A,<br>P681H	
Hpl 87	0.13%	C23271A,<br>A23403G,<br>A23456G,<br>C23604A	A570D,<br>D614G,<br>T632A,<br>P681H	

[a]The genomic region covered by amplicons A1 to A6 of the S-coding region is: A1: nucleotides 21,448 to 21,841; A2: nucleotides 21,727 to 21,217; A3: nucleotides 22,111 to 22,515; A4: nucleotides 22,487 to 22,882; A5: nucleotides 22,827 to 23,268; A6: nucleotides 23,259 to 23,645. Residue numbering according to NCBI reference sequence: NC_045512.2

[b]The SARS-CoV-2 genome residue numbering is according to the NCBI reference sequence: NC_045512.2. Those haplotypes which do not present any variation respect the reference sequence are called Wild type.

[c]Amino acid residues (single-letter code) are numbered from N- to C- terminus of each protein (ORF1b or S). Syn: synonymous mutation. All substitutions were located in S except those indicated with ORF1b. For amplicons A1 all haplotypes included deletion 21,765-21,770 that gives rise to deletion of amino acids H69 and V70 except Hpl 25. For amplicon A2 all haplotypes included deletion 21,765-21,770 that gives rise to deletion of amino acids H69, V70, except Hpl24, and 21,992-21,994 that gives rise to deletion of amino acid Y144. Hpl 35 of A1 and Hpl 24 of A2, includes deletion 21,764-21,769 that gives rise to deletion of amino acids I68 and H69.

[d]Haplotypes with amino acid substitutions or deletions characteristic of a different variant that the consensus variant.

[e]For Hpl 20, the V70T amino acid is due to the combination of deletion 21,765-21,770 and mutation T21,771C; and the deleted amino acids are I68 and H69.

Table S7. Haplotypes detected amplicons A1 to A6 of S – coding region in sample from patient Pt457[a].

<table>
<tr><th colspan="6">Spike A1</th></tr>
<tr><th>Haplotype number</th><th>Frequency</th><th>Nucleotide mutation[b]</th><th>Amino acid substitutions[c]</th><th>Nucleotide deletions[b]</th><th>Deleted amino acids[c]</th></tr>
<tr><td>Hpl 0</td><td>97.25%</td><td></td><td></td><td rowspan="13">21765-21770</td><td rowspan="13">H69Δ, V70Δ</td></tr>
<tr><td>Hpl 1</td><td>0.30%</td><td>A21489G</td><td>Syn</td></tr>
<tr><td>Hpl 2</td><td>0.29%</td><td>T21737C</td><td>F59L</td></tr>
<tr><td>Hpl 3</td><td>0.26%</td><td>T21529C</td><td>ORF1b:S2688P</td></tr>
<tr><td>Hpl 4</td><td>0.23%</td><td>T21655C</td><td>Syn</td></tr>
<tr><td>Hpl 5</td><td>0.22%</td><td>T21479C</td><td>ORF1b:L2671P</td></tr>
<tr><td>Hpl 6</td><td>0.22%</td><td>T21477C</td><td>Syn</td></tr>
<tr><td>Hpl 7</td><td>0.21%</td><td>T21521C</td><td>ORF1b:V2685A</td></tr>
<tr><td>Hpl 8</td><td>0.21%</td><td>T21539C</td><td>ORF1b:V2691A</td></tr>
<tr><td>Hpl 9</td><td>0.21%</td><td>A21450G</td><td>Syn</td></tr>
<tr><td>Hpl 10</td><td>0.21%</td><td>A21560G</td><td>Syn</td></tr>
<tr><td>Hpl 11</td><td>0.21%</td><td>A21779G</td><td>T73A</td></tr>
<tr><td>Hpl 12</td><td>0.19%</td><td>A21449G</td><td>ORF1b:E2661G</td></tr>
</table>

<table>
<tr><th colspan="6">Spike A2</th></tr>
<tr><th>Haplotype number</th><th>Frequency</th><th>Nucleotide mutation[b]</th><th>Amino acid substitutions[c]</th><th>Nucleotide deletions[b]</th><th>Deleted amino acids[c]</th></tr>
<tr><td>Hpl 0</td><td>94.58%</td><td></td><td></td><td rowspan="23">21765-21770, 21992-21994</td><td rowspan="14">H69Δ, V70Δ, Y144Δ</td></tr>
<tr><td>Hpl 1</td><td>0.35%</td><td>T21737C</td><td>F59L</td></tr>
<tr><td>Hpl 2</td><td>0.32%</td><td>T21829C</td><td>Syn</td></tr>
<tr><td>Hpl 3</td><td>0.31%</td><td>T21797C</td><td>F79L</td></tr>
<tr><td>Hpl 4</td><td>0.29%</td><td>A22029G</td><td>E156G</td></tr>
<tr><td>Hpl 5</td><td>0.28%</td><td>T21733C</td><td>Syn</td></tr>
<tr><td>Hpl 6</td><td>0.28%</td><td>T21755C</td><td>F65L</td></tr>
<tr><td>Hpl 7</td><td>0.26%</td><td>A22095G</td><td>D178G</td></tr>
<tr><td>Hpl 8</td><td>0.25%</td><td>A22102G</td><td>Syn</td></tr>
<tr><td>Hpl 9</td><td>0.25%</td><td>A22106G</td><td>K182E</td></tr>
<tr><td>Hpl 10</td><td>0.24%</td><td>T21831C</td><td>V90A</td></tr>
<tr><td>Hpl 11</td><td>0.24%</td><td>A22025G</td><td>S155G</td></tr>
<tr><td>Hpl 12</td><td>0.23%</td><td>A22105G</td><td>Syn</td></tr>
<tr><td>Hpl 13</td><td>0.23%</td><td>T21841C</td><td>Syn</td></tr>
<tr><td>Hpl 14[d]</td><td>0.23%</td><td>T21771C</td><td>V70T</td><td>I68Δ, H69Δ, Y144Δ</td></tr>
<tr><td>Hpl 15</td><td>0.22%</td><td>T22084C</td><td>Syn</td><td rowspan="8">H69Δ, V70Δ, Y144Δ</td></tr>
<tr><td>Hpl 16</td><td>0.22%</td><td>A22107G</td><td>K182R</td></tr>
<tr><td>Hpl 17</td><td>0.22%</td><td>A22101G</td><td>E180G</td></tr>
<tr><td>Hpl 18</td><td>0.21%</td><td>A22108G</td><td>Syn</td></tr>
<tr><td>Hpl 19</td><td>0.21%</td><td>T21808C</td><td>Syn</td></tr>
<tr><td>Hpl 20</td><td>0.21%</td><td>T22074C</td><td>V171A</td></tr>
<tr><td>Hpl 21</td><td>0.20%</td><td>T21735C</td><td>F58S</td></tr>
<tr><td>Hpl 22</td><td>0.20%</td><td>A21868G</td><td>Syn</td></tr>
</table>

Spike A3

Haplotype number	Frequency	Nucleotide mutation[b]	Amino acid substitutions[c]	Nucleotide deletions[b]	Deleted amino acids[c]
Hpl 0	97.36%	Wild type			
Hpl 1	0.41%	T22209C	L216P		
Hpl 2	0.33%	A22443G	D294G		
Hpl 3	0.30%	A22412G	T284A		
Hpl 4	0.26%	A22431G	D290G		
Hpl 5	0.23%	A22411G	Syn		
Hpl 6	0.23%	T22228C	Syn		
Hpl 7	0.23%	T22219C	Syn		
Hpl 8	0.22%	A22234G	Syn		
Hpl 9	0.22%	T22213C	Syn		
Hpl 10	0.22%	A22492G	Syn		

Spike A4

Haplotype number	Frequency	Nucleotide mutation[b]	Amino acid substitutions[c]	Nucleotide deletions[b]	Deleted amino acids[c]
Hpl 0	95.19%	Wild type			
Hpl 1	1.48%	T22690C	Syn		
Hpl 2	0.45%	G22487C	E309Q		
Hpl 3	0.34%	A22780G	Syn		
Hpl 4	0.29%	T22736C	F392L		
Hpl 5	0.27%	A22771G	Syn		
Hpl 6	0.25%	A22776G	D405G		
Hpl 7	0.23%	A22852G	Syn		
Hpl 8	0.23%	A22786G	Syn		
Hpl 9	0.22%	A22790G	I410V		
Hpl 10	0.22%	A22629G	K356R		
Hpl 11	0.21%	A22810G	Syn		
Hpl 12	0.21%	A22623G	N354S		
Hpl 13	0.19%	A22705G	Syn		
Hpl 14	0.19%	T22501C	Syn		

Spike A5

Haplotype number	Frequency	Nucleotide mutation[b]	Amino acid substitutions[c]	Nucleotide deletions[b]	Deleted amino acids[c]
Hpl 0	83.15%	A23063T	N501Y		
Hpl 1	0.31%	T22951C, A23063T	Syn, N501Y		
Hpl 2	0.29%	A23063T, A23265G	N501Y, D568G		
Hpl 3	0.29%	T23030C, A23063T	F490L, N501Y		
Hpl 4	0.28%	T22874C, A23063T	S438P, N501Y		
Hpl 5	0.28%	T22942C, A23063T	Syn, N501Y		

Hpl 6	0.27%	A22960G, A23063T	Syn, N501Y
Hpl 7	0.27%	A23063T, T23100C	N501Y, L513P
Hpl 8	0.27%	T22944C, A23063T	L461P, N501Y
Hpl 9	0.26%	T22873C, A23063T	Syn, N501Y
Hpl 10	0.26%	A22958G, A23063T	R466G, N501Y
Hpl 11	0.26%	A23063T, A23140G	N501Y, Syn
Hpl 12	0.26%	T22888C, A23063T	Syn, N501Y
Hpl 13	0.25%	A23063T, A23223G	N501Y, E554G
Hpl 14	0.25%	A22956G, A23063T	E465G, N501Y
Hpl 15	0.24%	A23063T, A23122G	N501Y, Syn
Hpl 16	0.24%	A23063T, T23112C	N501Y, L517P
Hpl 17	0.24%	T23017C, A23063T	Syn, N501Y
Hpl 18	0.24%	A23063T, T23245C	N501Y, Syn
Hpl 19	0.24%	A23063T, A23203G	N501Y, Syn
Hpl 20	0.24%	A23063T, A23169G	N501Y, N536S
Hpl 21	0.24%	T23026C, A23063T	Syn, N501Y
Hpl 22	0.23%	T23050C, A23063T	Syn, N501Y
Hpl 23	0.23%	A23063T, T23150C	N501Y, S530P
Hpl 24	0.23%	T22917C, A23063T	L452P, N501Y
Hpl 25	0.23%	A23063T, T23104C	N501Y, Syn
Hpl 26	0.23%	A23063T, A23263G	N501Y, Syn
Hpl 27	0.23%	A23063T, T23176C	N501Y, Syn
Hpl 28	0.22%	T22889C, A23063T	S443P, N501Y
Hpl 29	0.22%	A23063T, A23089G	N501Y, Syn
Hpl 30	0.22%	A23063T, A23207G	N501Y, T549A
Hpl 31	0.22%	A23063T, A23080G	N501Y, Syn
Hpl 32	0.22%	T22884C, A23063T	L441P, N501Y
Hpl 33	0.22%	T22999C, A23063T	Syn, N501Y
Hpl 34	0.22%	A23014G, A23063T	Syn, N501Y

Hpl 35	0.22%	A23063T, T23215C	N501Y, Syn
Hpl 36	0.22%	T23035C, A23063T	Syn, N501Y
Hpl 37	0.21%	T22882C, A23063T	Syn, N501Y
Hpl 38	0.21%	A23063T, A23261G	N501Y, R567G
Hpl 39	0.21%	T22937C, A23063T	S459P, N501Y
Hpl 40	0.21%	T23010C, A23063T	V483A, N501Y
Hpl 41	0.21%	A23063T, A23201G	N501Y, T547A
Hpl 42	0.21%	A22935G, A23063T	K458R, N501Y
Hpl 43	0.21%	A23063T, A23233G	N501Y, Syn
Hpl 44	0.21%	A23063T, A23110G	N501Y, Syn
Hpl 45	0.20%	A23063T, T23178C	N501Y, V539A
Hpl 46	0.20%	T22896C, A23063T	V445A, N501Y
Hpl 47	0.20%	A22962G, A23063T	D467G, N501Y
Hpl 48	0.20%	T23032C, A23063T	Syn, N501Y
Hpl 49	0.20%	A23063T, T23225C	N501Y, S555P
Hpl 50	0.20%	T22876C, A23063T	Syn, N501Y
Hpl 51	0.20%	A23063T, T23133C	N501Y, V524A
Hpl 52	0.20%	A23063T, A23229G	N501Y, N556S
Hpl 53	0.19%	A23063T, T23197C	N501Y, Syn
Hpl 54	0.19%	A23063T, A23125G	N501Y, Syn
Hpl 55	0.19%	T22900C, A23063T	Syn, N501Y
Hpl 56	0.19%	A23063T, T23102C	N501Y, S514P
Hpl 57	0.19%	A23063T, A23251G	N501Y, Syn
Hpl 58	0.19%	A23056G, A23063T	Syn, N501Y
Hpl 59	0.19%	A23063T, T23182C	N501Y, Syn
Hpl 60	0.19%	A23063T, T23163C	N501Y, V534A
Hpl 61	0.19%	A23063T, T23214C	N501Y, V551A
Hpl 62	0.19%	A23063T, T23241C	N501Y, L560P
Hpl 63	0.18%	T23008C, A23063T	Syn, N501Y

Hpl 64	0.18%	T23051C, A23063T	F497L, N501Y		
Hpl 65	0.18%	A23063T, T23074C	N501Y, Syn		
Hpl 66	0.18%	T22928C, A23063T	F456L, N501Y		
Hpl 67	0.18%	A23063T, A23267G	N501Y, Syn		
Hpl 68	0.18%	A22996G, A23063T	Syn, N501Y		
Hpl 69	0.18%	A22994G, A23063T	T478A, N501Y		
Hpl 70	0.18%	T23036C, A23063T	Syn, N501Y		
Hpl 71	0.18%	A23063T, T23068C	N501Y, Syn		
Hpl 72	0.18%	A23063T, A23209G	N501Y, Syn		
Hpl 73	0.18%	A23063T, A23118G	N501Y, H519R		
Hpl 74	0.18%	A23063T, A23148G	N501Y, K529R		
Hpl 75	0.17%	A23063T, A23168G	N501Y, N536D		
Hpl 76	0.17%	A23063T, A23129G	N501Y, T523A		
Hpl 77	0.17%	A23063T, T23247C	N501Y, F562S		
Hpl 78	0.16%	A23063T, T23070C	N501Y, V503A		

Spike A6

Haplotype number	Frequency	Nucleotide mutation[b]	Amino acid substitutions[c]	Nucleotide deletions[b]	Deleted amino acids[c]
Hpl 0	87.84%	C23271A, A23403G, C23604A	A570D, D614G, P681H,		
Hpl 1	0.31%	C23271A, T23332C, A23403G, C23604A	A570D, Syn, D614G, P681H		
Hpl 2	0.30%	C23271A, T23346C, A23403G, C23604A	A570D, V595A, D614G, P681H		
Hpl 3	0.29%	C23271A, T23391C, A23403G, C23604A	A570D, V610A, D614G, P681H		
Hpl 4	0.29%	C23271A, A23403G, A23495G, C23604A	A570D, D614G, T645A, P681H		
Hpl 5	0.28%	C23271A, T23394C, A23403G, C23604A	A570D, L611P, D614G, P681H		
Hpl 6	0.28%	C23271A, A23403G,	A570D, D614G,		

		A23566G, C23604A	Syn, P681H
Hpl 7	0.28%	C23271A, A23403G, A23550G, C23604A	A570D, D614G, D663G, P681H
Hpl 8	0.27%	C23271A, A23403G, T23452C, C23604A	A570D, D614G, Syn, P681H
Hpl 9	0.26%	C23271A, A23403G, T23487C, C23604A	A570D, D614G, V642A, P681H
Hpl 10	0.25%	C23271A, A23403G, T23479C, C23604A	A570D, D614G, Syn, P681H
Hpl 11	0.25%	C23271A, A23403G, A23419G, C23604A	A570D, D614G, Syn, P681H
Hpl 12	0.25%	C23271A, T23333C, A23403G, C23604A	A570D, S591P, D614G, P681H
Hpl 13	0.24%	C23271A, A23403G, A23594G, C23604A	A570D, D614G, T678A, P681H
Hpl 14	0.24%	C23271A, A23366G, A23403G, C23604A	A570D, T602A, D614G, P681H
Hpl 15	0.24%	C23271A, T23350C, A23403G, C23604A	A570D, Syn, D614G, P681H
Hpl 16	0.24%	C23271A, A23403G, T23421C, C23604A	A570D, D614G, V620A, P681H
Hpl 17	0.24%	C23271A, T23289C, A23403G, C23604A	A570D, V576A, D614G, P681H
Hpl 18	0.23%	C23271A, A23403G, A23503G, C23604A	A570D, D614G, Syn, P681H
Hpl 19	0.23%	C23271A, A23403G, A23440G, C23604A	A570D, D614G, Syn, P681H
Hpl 20	0.23%	C23271A, T23296C, A23403G, C23604A	A570D, Syn, D614G, P681H

Hpl 21	0.23%	C23271A,<br>T23335C,<br>A23403G,<br>C23604A	A570D,<br>Syn,<br>D614G,<br>P681H
Hpl 22	0.23%	C23271A,<br>T23284C,<br>A23403G,<br>C23604A	A570D,<br>Syn,<br>D614G,<br>P681H
Hpl 23	0.23%	C23271A,<br>T23374C,<br>A23403G,<br>C23604A	A570D,<br>Syn,<br>D614G,<br>P681H
Hpl 24	0.23%	C23271A,<br>A23403G,<br>A23588G,<br>C23604A	A570D,<br>D614G,<br>T676A,<br>P681H
Hpl 25	0.23%	C23271A,<br>A23403G,<br>T23406C,<br>C23604A	A570D,<br>D614G,<br>V615A,<br>P681H
Hpl 26	0.23%	C23271A,<br>A23403G,<br>A23414G,<br>C23604A	A570D,<br>D614G,<br>T618A,<br>P681H
Hpl 27	0.22%	C23271A,<br>A23403G,<br>T23489C,<br>C23604A	A570D,<br>D614G,<br>F643L,<br>P681H
Hpl 28	0.22%	C23271A,<br>T23352C,<br>A23403G,<br>C23604A	A570D,<br>V597A,<br>D614G,<br>P681H
Hpl 29	0.22%	C23271A,<br>T23287C,<br>A23403G,<br>C23604A	A570D,<br>Syn,<br>D614G,<br>P681H
Hpl 30	0.22%	C23271A,<br>A23403G,<br>A23524G,<br>C23604A	A570D,<br>D614G,<br>Syn,<br>P681H
Hpl 31	0.21%	C23271A,<br>A23403G,<br>T23529C,<br>C23604A	A570D,<br>D614G,<br>V656A,<br>P681H
Hpl 32	0.21%	C23271A,<br>A23403G,<br>T23433C,<br>C23604A	A570D,<br>D614G,<br>I624T,<br>P681H
Hpl 33	0.21%	C23271A,<br>A23403G,<br>T23548C,<br>C23604A	A570D,<br>D614G,<br>Syn,<br>P681H
Hpl 34	0.21%	C23271A,<br>T23344C,<br>A23403G,<br>C23604A	A570D,<br>Syn,<br>D614G,<br>P681H
Hpl 35	0.21%	C23271A,<br>T23330C,<br>A23403G,	A570D,<br>C590R,<br>D614G,

		C23604A	P681H
Hpl 36	0.20%	C23271A,<br>A23403G,<br>T23427C,<br>C23604A	A570D,<br>D614G,<br>V622A,<br>P681H
Hpl 37	0.20%	C23271A,<br>T23307C,<br>A23403G,<br>C23604A	A570D,<br>L582P,<br>D614G,<br>P681H
Hpl 38	0.20%	C23271A,<br>T23392C,<br>A23403G,<br>C23604A	A570D,<br>Syn,<br>D614G,<br>P681H
Hpl 39	0.20%	C23271A,<br>A23403G,<br>T23428C,<br>C23604A	A570D,<br>D614G,<br>Syn,<br>P681H
Hpl 40	0.20%	C23271A,<br>A23276G,<br>A23403G,<br>C23604A	A570D,<br>T572A,<br>D614G,<br>P681H
Hpl 41	0.20%	C23271A,<br>T23385C,<br>A23403G,<br>C23604A	A570D,<br>V608A,<br>D614G,<br>P681H
Hpl 42	0.20%	C23271A,<br>A23403G,<br>T23466C,<br>C23604A	A570D,<br>D614G,<br>V635A,<br>P681H
Hpl 43	0.20%	C23271A,<br>T23341C,<br>A23403G,<br>C23604A	A570D,<br>Syn,<br>D614G,<br>P681H
Hpl 44	0.19%	C23271A,<br>A23301G,<br>A23403G,<br>C23604A	A570D,<br>Q580R,<br>D614G,<br>P681H
Hpl 45	0.19%	C23271A,<br>A23326G,<br>A23403G,<br>C23604A	A570D,<br>Syn,<br>D614G,<br>P681H
Hpl 46	0.19%	C23271A,<br>T23293C,<br>A23403G,<br>C23604A	A570D,<br>Syn,<br>D614G,<br>P681H
Hpl 47	0.19%	C23271A,<br>T23313C,<br>A23403G,<br>C23604A	A570D,<br>I584T,<br>D614G,<br>P681H
Hpl 48	0.19%	C23271A,<br>A23403G,<br>A23476G,<br>C23604A	A570D,<br>D614G,<br>Syn,<br>P681H
Hpl 49	0.19%	C23271A,<br>A23403G,<br>A23552G,<br>C23604A	A570D,<br>D614G,<br>I664V,<br>P681H
Hpl 50	0.19%	C23271A,<br>A23403G,	A570D,<br>D614G,

		C23604A, A23623G	P681H, Syn
Hpl 51	0.18%	C23271A, A23403G, T23480C, C23604A	A570D, D614G, S640P, P681H
Hpl 52	0.18%	C23271A, A23403G, A23531G, C23604A	A570D, D614G, N657D, P681H
Hpl 53[e]	0.18%	C23271A, A23403G, C23604G,	A570D, D614G, P681R
Hpl 54	0.18%	C23271A, A23319G, A23403G, C23604A	A570D, D586G, D614G, P681H

[a]The genomic region covered by amplicons A1 to A6 of the S-coding region is: A1: nucleotides 21,448 to 21,841; A2: nucleotides 21,727 to 21,217; A3: nucleotides 22,111 to 22,515; A4: nucleotides 22,487 to 22,882; A5: nucleotides 22,827 to 23,268; A6: nucleotides 23,259 to 23,645. Residue numbering according to NCBI reference sequence: NC_045512.2

[b]The SARS-CoV-2 genome residue numbering is according to the NCBI reference sequence: NC_045512.2. Those haplotypes which do not present any variation respect the reference sequence are called Wild type.

[c]Amino acid residues (single-letter code) are numbered from N- to C- terminus of each protein (ORF1b or S). Syn: synonymous mutation. All substitutions were located in S except those indicated with ORF1b. For amplicons A1 all haplotypes included deletion 21,765-21,770 that gives rise to deletion of amino acids H69 and V70 except Hpl 25. For amplicon A2 all haplotypes included deletion 21,765-21,770 that gives rise to deletion of amino acids H69, V70, except Hpl 24, and 21,992-21,994 that gives rise to deletion of amino acid Y144.

[d]For Hpl 14, the V70T amino acid is due to the combination of deletion 21,765-21,770 and mutation T21,771C; and the deleted amino acids are I68 and H69.

[e]Haplotypes with amino acid substitutions or deletions characteristic of a different variant that the consensus variant.

Table S8. Haplotypes detected amplicons A1 to A6 of S-coding region in sample from patient Pt458[a].

<table>
<tr><th colspan="6">Spike A1</th></tr>
<tr><th>Haplotype number</th><th>Frequency</th><th>Nucleotide mutations[b]</th><th>Amino acid substitutions[c]</th><th>Nucleotide deletions[b]</th><th>Deleted amino acids[c]</th></tr>
<tr><td>Hpl 0</td><td>94.35%</td><td>T21579C</td><td>V6A</td><td rowspan="24">21765-21770</td><td rowspan="24">H69Δ, V70Δ</td></tr>
<tr><td>Hpl 1</td><td>0.32%</td><td>T21579C,
T21737C</td><td>V6A,
F59L</td></tr>
<tr><td>Hpl 2</td><td>0.30%</td><td>T21477C,
T21579C</td><td>Syn,
V6A</td></tr>
<tr><td>Hpl 3</td><td>0.27%</td><td>T21579C,
A21779G</td><td>V6A,
T73A</td></tr>
<tr><td>Hpl 4</td><td>0.25%</td><td>T21579C,
T21596C</td><td>V6A,
S12P</td></tr>
<tr><td>Hpl 5</td><td>0.25%</td><td>T21542C,
T21579C</td><td>ORF1b:L2692P,
V6A</td></tr>
<tr><td>Hpl 6</td><td>0.25%</td><td>A21489G,
T21579C</td><td>Syn,
V6A</td></tr>
<tr><td>Hpl 7</td><td>0.24%</td><td>T21479C,
T21579C</td><td>ORF1b:L2671P,
V6A</td></tr>
<tr><td>Hpl 8</td><td>0.23%</td><td>T21475C,
T21579C</td><td>ORF1b:S2670P,
V6A</td></tr>
<tr><td>Hpl 9</td><td>0.23%</td><td>T21579C,
T21733C</td><td>V6A,
Syn</td></tr>
<tr><td>Hpl 10</td><td>0.22%</td><td>T21539C,
T21579C</td><td>ORF1b:V2691A,
V6A</td></tr>
<tr><td>Hpl 11</td><td>0.22%</td><td>T21579C,
T21580C</td><td>V6A,
Syn</td></tr>
<tr><td>Hpl 12</td><td>0.22%</td><td></td><td></td></tr>
<tr><td>Hpl 13</td><td>0.22%</td><td>T21579C,
A21794G</td><td>V6A,
R78G</td></tr>
<tr><td>Hpl 14</td><td>0.21%</td><td>T21497C,
T21579C</td><td>ORF1b:L2677P,
V6A</td></tr>
<tr><td>Hpl 15</td><td>0.21%</td><td>T21579C,
A21720G</td><td>V6A,
D53G</td></tr>
<tr><td>Hpl 16</td><td>0.21%</td><td>T21579C,
T21594C</td><td>V6A,
V11A</td></tr>
<tr><td>Hpl 17</td><td>0.21%</td><td>T21579C,
T21669C</td><td>V6A,
V36A</td></tr>
<tr><td>Hpl 18</td><td>0.21%</td><td>T21579C,
A21685G</td><td>V6A,
Syn</td></tr>
<tr><td>Hpl 19</td><td>0.20%</td><td>T21521C,
T21579C</td><td>ORF1b:V2685A,
V6A</td></tr>
<tr><td>Hpl 20</td><td>0.20%</td><td>T21579C,
T21755C</td><td>V6A,
F65L</td></tr>
<tr><td>Hpl 21</td><td>0.20%</td><td>T21579C,
A21626G</td><td>V6A,
T22A</td></tr>
<tr><td>Hpl 22</td><td>0.20%</td><td>T21579C,
T21657C</td><td>V6A,
F32S</td></tr>
<tr><td>Hpl 23</td><td>0.20%</td><td>T21572C,
T21579C</td><td>F4L,
V6A</td></tr>
<tr><td>Hpl 24</td><td>0.20%</td><td>Wild type</td><td></td><td></td><td></td></tr>
<tr><td>Hpl 25</td><td>0.20%</td><td>A21560G,
T21579C</td><td>Syn,
V6A</td><td>21765-21770</td><td>H69Δ, V70Δ</td></tr>
<tr><th colspan="6">Spike A2</th></tr>
</table>

<table>
<tr><th>Haplotype number</th><th>Frequency</th><th>Nucleotide mutations[b]</th><th>Amino acid substitutions[c]</th><th>Nucleotide deletions[b]</th><th>Deleted amino acids[c]</th></tr>
<tr><td>Hpl 0</td><td>92.70%</td><td></td><td></td><td rowspan="28">21765-21770, 21992-21994</td><td rowspan="28">H69Δ, V70Δ, Y144Δ</td></tr>
<tr><td>Hpl 1</td><td>0.48%</td><td>T21773C</td><td>S71P</td></tr>
<tr><td>Hpl 2</td><td>0.37%</td><td>A22102G</td><td>Syn</td></tr>
<tr><td>Hpl 3</td><td>0.37%</td><td>T21737C</td><td>F59L</td></tr>
<tr><td>Hpl 4</td><td>0.31%</td><td>T21797C</td><td>F79L</td></tr>
<tr><td>Hpl 5</td><td>0.30%</td><td>T21755C</td><td>F65L</td></tr>
<tr><td>Hpl 6</td><td>0.29%</td><td>T21841C</td><td>Syn</td></tr>
<tr><td>Hpl 7</td><td>0.29%</td><td>A22095G</td><td>D178G</td></tr>
<tr><td>Hpl 8</td><td>0.27%</td><td>A22106G</td><td>K182E</td></tr>
<tr><td>Hpl 9</td><td>0.26%</td><td>T21733C</td><td>Syn</td></tr>
<tr><td>Hpl 10</td><td>0.26%</td><td>A22025G</td><td>S155G</td></tr>
<tr><td>Hpl 11</td><td>0.25%</td><td>T21842C</td><td>S94P</td></tr>
<tr><td>Hpl 12[d]</td><td>0.25%</td><td>T21810C</td><td>V83A</td></tr>
<tr><td>Hpl 13</td><td>0.25%</td><td>T21908C</td><td>S116P</td></tr>
<tr><td>Hpl 14</td><td>0.24%</td><td>T21831C</td><td>V90A</td></tr>
<tr><td>Hpl 15</td><td>0.23%</td><td>T21829C</td><td>Syn</td></tr>
<tr><td>Hpl 16</td><td>0.23%</td><td>A21868G</td><td>Syn</td></tr>
<tr><td>Hpl 17</td><td>0.23%</td><td>T21756C</td><td>F65S</td></tr>
<tr><td>Hpl 18</td><td>0.22%</td><td>T22084C</td><td>Syn</td></tr>
<tr><td>Hpl 19</td><td>0.22%</td><td>A22107G</td><td>K182R</td></tr>
<tr><td>Hpl 20</td><td>0.22%</td><td>T21735C</td><td>F58S</td></tr>
<tr><td>Hpl 21</td><td>0.22%</td><td>A22029G</td><td>E156G</td></tr>
<tr><td>Hpl 22</td><td>0.21%</td><td>T21747C</td><td>V62A</td></tr>
<tr><td>Hpl 23</td><td>0.21%</td><td>A22105G</td><td>Syn</td></tr>
<tr><td>Hpl 24</td><td>0.20%</td><td>A22108G</td><td>Syn</td></tr>
<tr><td>Hpl 25</td><td>0.19%</td><td>A22008G</td><td>N149S</td></tr>
<tr><td>Hpl 26</td><td>0.19%</td><td>T22074C</td><td>V171A</td></tr>
<tr><td>Hpl 27</td><td>0.19%</td><td>T21854C</td><td>S98P</td></tr>
<tr><td>Hpl 28[e]</td><td>0.19%</td><td>T21771C</td><td>V70T</td><td>21765-21770, 21992-21994</td><td>I68Δ, H69Δ, Y144Δ</td></tr>
<tr><td>Hpl 29</td><td>0.15%</td><td>G21898A</td><td>Syn</td><td></td><td></td></tr>
</table>

Spike A3

Haplotype number	Frequency	Nucleotide mutations[b]	Amino acid substitutions[c]	Nucleotide deletions[b]	Deleted amino acids[c]
Hpl 0	94.26%	Wild type			
Hpl 1	0.33%	T22209C	L216P		
Hpl 2	0.32%	A22492G	Syn		
Hpl 3	0.32%	A22412G	T284A		
Hpl 4	0.31%	T22291C	Syn		
Hpl 5	0.30%	A22443G	D294G		
Hpl 6	0.25%	A22431G	D290G		
Hpl 7	0.25%	T22274C	F238L		
Hpl 8	0.24%	A22457G	T299A		
Hpl 9	0.24%	T22384C	Syn		
Hpl 10	0.24%	T22478C	F306L		

Hpl 11	0.23%	T22371C	L270P		
Hpl 12	0.23%	A22337G	T259A		
Hpl 13	0.22%	T22207C	Syn		
Hpl 14	0.22%	T22435C	Syn		
Hpl 15	0.22%	A22234G	Syn		
Hpl 16	0.22%	A22375G	Syn		
Hpl 17	0.21%	T22228C	Syn		
Hpl 18	0.21%	A22301G	S247G		
Hpl 19	0.21%	T22142C	F194L		
Hpl 20	0.21%	T22324C	Syn		
Hpl 21[d]	0.20%	C22227T	A222V		
Hpl 22	0.20%	A22474G	Syn		
Hpl 23	0.20%	T22219C	Syn		
Hpl 24	0.20%	A22456G	Syn		

Spike A4

Haplotype number	Frequency	Nucleotide mutations[b]	Amino acid substitutions[c]	Nucleotide deletions[b]	Deleted amino acids[c]
Hpl 0	93.58%	Wild type			
Hpl 1	1.53%	T22690C	Syn		
Hpl 2	0.37%	A22780G	Syn		
Hpl 3	0.32%	T22736C	F392L		
Hpl 4	0.28%	A22786G	Syn		
Hpl 5	0.26%	A22852G	Syn		
Hpl 6	0.26%	A22776G	D405G		
Hpl 7	0.26%	G22487C	E309Q		
Hpl 8	0.24%	T22507C	Syn		
Hpl 9	0.23%	A22629G	K356R		
Hpl 10	0.22%	A22812G	K417R		
Hpl 11	0.22%	A22784G	R408G		
Hpl 12	0.22%	T22514C	F318L		
Hpl 13	0.22%	T22501C	Syn		
Hpl 14	0.21%	A22631G	R357G		
Hpl 15	0.21%	T22611C	V350A		
Hpl 16	0.21%	A22805G	T415A		
Hpl 17	0.20%	T22709C	S383P		
Hpl 18	0.20%	A22633G	Syn		
Hpl 19	0.20%	A22519G	Syn		
Hpl 20	0.19%	A22810G	Syn		
Hpl 21	0.19%	A22623G	N354S		
Hpl 22	0.19%	A22771G	Syn		

Spike A5

Haplotype number	Frequency	Nucleotide mutations[b]	Amino acid substitutions[c]	Nucleotide deletions[b]	Deleted amino acids[c]
Hpl 0	94.95%	A23063T	N501Y		
Hpl 1	0.34%	A23063T, A23265G	N501Y, D568G		

Hpl 2	0.29%	A23063T, A23263G	N501Y, Syn		
Hpl 3	0.27%	T23030C, A23063T	F490L, N501Y		
Hpl 4	0.26%	Wild type			
Hpl 5	0.25%	T22876C, A23063T	Syn, N501Y		
Hpl 6	0.25%	A23063T, A23203G	N501Y, Syn		
Hpl 7	0.24%	A22958G, A23063T	R466G, N501Y		
Hpl 8	0.23%	A23063T, T23100C	N501Y, L513P		
Hpl 9	0.23%	A23063T, A23223G	N501Y, E554G		
Hpl 10	0.22%	A22960G, A23063T	Syn, N501Y		
Hpl 11	0.22%	T22951C, A23063T	Syn, N501Y		
Hpl 12	0.21%	A23063T, A23140G	N501Y, Syn		
Hpl 13	0.21%	T23026C, A23063T	Syn, N501Y		
Hpl 14	0.21%	A23063T, T23214C	N501Y, V551A		
Hpl 15	0.21%	A23063T, T23245C	N501Y, Syn		
Hpl 16	0.21%	A23063T, A23169G	N501Y, N536S		
Hpl 17	0.20%	A23063T, A23122G	N501Y, Syn		
Hpl 18	0.20%	A23063T, T23225C	N501Y, S555P		
Hpl 19	0.20%	A22935G, A23063T	K458R, N501Y		
Hpl 20	0.20%	T23050C, A23063T	Syn, N501Y		
Hpl 21	0.20%	T22944C, A23063T	L461P, N501Y		
Hpl 22	0.19%	A22956G, A23063T	E465G, N501Y		

Spike A6

Haplotype number	Frequency	Nucleotide mutations[b]	Amino acid substitutions[c]	Nucleotide deletions[b]	Deleted amino acids[c]
Hpl 0	95.88%	C23271A, A23403G, C23604A	A570D, D614G, P681H		
Hpl 1	0.53%	C23271A, A23403G, A23544G, C23604A	A570D, D614G, E661G, P681H		
Hpl 2	0.51%	A23403G	D614G		
Hpl 3	0.25%	C23271A, A23403G, A23550G, C23604A	A570D, D614G, D663G, P681H		
Hpl 4	0.24%	C23271A, A23403G,	A570D, D614G,		

		A23594G, C23604A	T678A, P681H
Hpl 5	0.24%	C23271A, T23332C, A23403G, C23604A	A570D, Syn, D614G, P681H
Hpl 6	0.23%	C23271A, T23391C, A23403G, C23604A	A570D, V610A, D614G, P681H
Hpl 7	0.23%	C23271A, T23346C, A23403G, C23604A	A570D, V595A, D614G, P681H
Hpl 8	0.22%	C23271A, T23289C, A23403G, C23604A	A570D, V576A, D614G, P681H
Hpl 9	0.22%	C23271A, A23403G, A23588G, C23604A	A570D, D614G, T676A, P681H
Hpl 10	0.22%	C23271A, A23403G, T23452C, C23604A	A570D, D614G, Syn, P681H
Hpl 11	0.21%	C23271A, T23287C, A23403G, C23604A	A570D, Syn, D614G, P681H
Hpl 12	0.21%	C23271A, T23344C, A23403G, C23604A	A570D, Syn, D614G, P681H
Hpl 13	0.21%	C23271A, A23403G, T23421C, C23604A	A570D, D614G, V620A, P681H
Hpl 14	0.21%	C23271A, A23403G, T23466C, C23604A	A570D, D614G, V635A, P681H
Hpl 15	0.20%	C23271A, A23403G, A23495G, C23604A	A570D, D614G, T645A, P681H
Hpl 16	0.20%	C23271A, A23403G, T23487C, C23604A	A570D, D614G, V642A, P681H

[a]The genomic region covered by amplicons A1 to A6 of the S-coding region is: A1: nucleotides 21,448 to 21,841; A2: nucleotides 21,727 to 21,217; A3: nucleotides 22,111 to 22,515; A4: nucleotides 22,487 to 22,882; A5: nucleotides 22,827 to 23,268; A6: nucleotides 23,259 to 23,645. Residue numbering according to NCBI reference sequence: NC_045512.2

[b]The SARS-CoV-2 genome residue numbering is according to the NCBI reference sequence: NC_045512.2. Those haplotypes which do not present any variation with respect the reference sequence are termed Wild type.

[c]Amino acid residues (single-letter code) are numbered from N- to C- terminus of each protein (ORF1b or S); Syn: synonymous mutation. All substitutions were located in S except those indicated with ORF1b. For amplicons A1 all haplotypes included deletion 21,765-21,770 that gives rise to deletion of amino acids H69 and V70 except Hpl 24. For amplicon A2 all haplotypes included deletion 21,765-21,770 that gives rise to deletion of amino acids H69, V70, except Hpl 29, and 21,992-21,994 that gives rise to deletion of amino acid Y144.

[d]Haplotypes with amino acid substitutions or deletions characteristic of a different variant that the consensus variant.

[e]For Hpl 28, the V70T amino acid is due to the combination of deletion 21,765-21,770 and mutations T21,771C; and the deleted amino acids are I68 and H69.

Table S9. Haplotypes detected amplicons A1 to A6 of S – coding region in sample from patient Pt459[a].

Spike A1

Haplotype number	Frequency	Nucleotide mutations[b]	Amino acid substitutions[c]	Nucleotide deletions[b]	Deleted amino acids[c]
Hpl 0	95.64%	Wild type			
Hpl 1	0.33%	T21737C	F59L		
Hpl 2	0.29%	A21779G	T73A		
Hpl 3	0.28%	T21477C	Syn		
Hpl 4	0.28%	T21628C	Syn		
Hpl 5	0.27%	T21475C	ORF1b:S2670P		
Hpl 6	0.26%	A21720G	D53G		
Hpl 7	0.25%	A21794G	R78G		
Hpl 8	0.24%	A21489G	Syn		
Hpl 9	0.24%	A21825G	D88G		
Hpl 10	0.24%	T21479C	ORF1b:L2671P		
Hpl 11	0.23%	T21539C	ORF1b:V2691A		
Hpl 12	0.23%	T21655C	Syn		
Hpl 13[d]	0.23%	C21575T	L5F		
Hpl 14	0.22%	T21542C	ORF1b:L2692P		
Hpl 15	0.22%	T21540C	Syn		
Hpl 16	0.21%	T21594C	V11A		
Hpl 17	0.20%	T21596C	S12P		
Hpl 18	0.15%	T21579C	V6A		

Spike A2

Haplotype number	Frequency	Nucleotide mutations[b]	Amino acid substitutions[c]	Nucleotide deletions[b]	Deleted amino acids[c]
Hpl 0	92.27%	G21898A	Syn		
Hpl 1	0.38%	T21737C, G21898A	F59L, Syn		
Hpl 2	0.31%	T21771C, G21898A	V70A, Syn		
Hpl 3	0.30%	G21898A, A22102G	Syn, Syn		
Hpl 4	0.29%	T21773C, G21898A	S71P, Syn		
Hpl 5	0.27%	T21733C, G21898A	Syn, Syn		
Hpl 6	0.27%	G21898A, A22107G	Syn, K182R		
Hpl 7	0.26%	G21898A, A22095G	Syn, D178G		
Hpl 8	0.25%	G21898A, A22108G	Syn, Syn		
Hpl 9	0.25%	T21755C, G21898A	F65L, Syn		
Hpl 10	0.25%	T21841C, G21898A	Syn, Syn		
Hpl 11	0.24%	G21898A, T22084C	Syn, Syn		

Hpl 12	0.24%	G21898A, A22106G	Syn, K182E		
Hpl 13	0.23%	G21898A, A22029G	Syn, E156G		
Hpl 14	0.23%	T21842C, G21898A	S94P, Syn		
Hpl 15	0.23%	T21797C, G21898A	F79L, Syn		
Hpl 16	0.23%	G21898A, A22025G	Syn, S155G		
Hpl 17	0.22%	T21831C, G21898A	V90A, Syn		
Hpl 18	0.21%	T21854C, G21898A	S98P, Syn		
Hpl 19	0.21%	T21808C, G21898A	Syn, Syn		
Hpl 20	0.21%	G21898A, A22101G	Syn, E180G		
Hpl 21	0.21%	G21898A, A22013G	Syn, S151G		
Hpl 22	0.21%	G21898A, A21957G	Syn, E132G		
Hpl 23	0.20%	G21898A, A22105G	Syn, Syn		
Hpl 24	0.20%	T21735C, G21898A	F58S, Syn		
Hpl 25	0.20%	G21898A, A22008G	Syn, N149S		
Hpl 26[d]	0.20%	G21898A	Syn	21992-21994	Y144Δ
Hpl 27	0.20%	G21898A, T21990C	Syn, V143A		
Hpl 28	0.20%	A21868G, G21898A	Syn, Syn		
Hpl 29[d]	0.20%	T21810C, G21898A	V83A, Syn		
Hpl 30	0.19%	G21898A, T22074C	Syn, V171A		
Hpl 31	0.19%	T21760C, G21898A	Syn, Syn		
Hpl 32	0.18%	G21898A, T22076C	Syn, S172P		
Hpl 33[d]	0.16%			21765-21770, 21992-21994	H69Δ, V70Δ, Y144Δ
Hpl 34	0.11%	G21898A	Syn	21888-21891	L110Δ

Spike A3

Haplotype number	Frequency	Nucleotide mutations[b]	Amino acid substitutions[c]	Nucleotide deletions[b]	Deleted amino acids[c]
Hpl 0	95.45%	C22227T	A222V		
Hpl 1	0.33%	T22209C, C22227T	L216P, A222V		
Hpl 2	0.31%	C22227T, A22492G	A222V, Syn		
Hpl 3	0.30%	C22227T, A22412G	A222V, T284A		
Hpl 4	0.27%	C22227T, T22291C	A222V, Syn		

Hpl 5	0.26%	C22227T,<br>A22443G	A222V,<br>D294G		
Hpl 6	0.25%	C22227T,<br>T22384C	A222V,<br>Syn		
Hpl 7	0.24%	C22227T,<br>A22457G	A222V,<br>T299A		
Hpl 8	0.24%	C22227T,<br>A22431G	A222V,<br>D290G		
Hpl 9	0.23%	C22227T,<br>A22411G	A222V,<br>Syn		
Hpl 10	0.23%	C22227T,<br>A22496G	A222V,<br>I312V		
Hpl 11	0.22%	C22227T,<br>A22301G	A222V,<br>S247G		
Hpl 12	0.22%	C22227T,<br>T22371C	A222V,<br>L270P		
Hpl 13	0.22%	C22227T,<br>A22375G	A222V,<br>Syn		
Hpl 14	0.21%	C22227T,<br>T22478C	A222V,<br>F306L		
Hpl 15	0.21%	C22227T,<br>T22274C	A222V,<br>F238L		
Hpl 16	0.21%	T22219C,<br>C22227T	Syn,<br>A222V		
Hpl 17	0.21%	T22207C,<br>C22227T	Syn,<br>A222V		
Hpl 18	0.20%	C22227T,<br>A22382G	A222V,<br>T274A		
Hpl 19	0.19%	C22227T,<br>T22282C	A222V,<br>Syn		

Spike A4

**Haplotype number**	**Frequency**	**Nucleotide mutations[b]**	**Amino acid substitutions[c]**	**Nucleotide deletions[b]**	**Deleted amino acids[c]**
Hpl 0	92.02%	Wild type			
Hpl 1	1.95%	G22487C	E309Q		
Hpl 2	1.58%	T22690C	Syn		
Hpl 3	0.29%	T22736C	F392L		
Hpl 4	0.29%	A22780G	Syn		
Hpl 5	0.25%	A22492G	Syn		
Hpl 6	0.24%	A22786G	Syn		
Hpl 7	0.24%	T22521C	V320A		
Hpl 8	0.24%	A22776G	D405G		
Hpl 9	0.22%	A22771G	Syn		
Hpl 10	0.22%	A22623G	N354S		
Hpl 11	0.22%	T22795C	Syn		
Hpl 12	0.22%	A22582G	Syn		
Hpl 13	0.21%	A22705G	Syn		
Hpl 14	0.21%			22586-22588	F342Δ
Hpl 15	0.21%	T22514C	F318L		
Hpl 16	0.21%	A22810G	Syn		
Hpl 17	0.21%	T22709C	S383P		
Hpl 18	0.21%	T22497C	I312T		

Hpl 19	0.20%	A22633G	Syn		
Hpl 20	0.20%	T22711C	Syn		
Hpl 21	0.20%	A22852G	Syn		
Hpl 22	0.19%	A22812G	K417R		

Spike A5

Haplotype number	Frequency	Nucleotide mutations[b]	Amino acid substitutions[c]	Nucleotide deletions[b]	Deleted amino acids[c]
Hpl 0	93.36%	Wild type			
Hpl 1	0.29%	A23265G	D568G		
Hpl 2	0.29%	T22874C	S438P		
Hpl 3[d]	0.28%	A23063T	N501Y		
Hpl 4	0.27%	T23030C	F490L		
Hpl 5	0.27%	T22951C	Syn		
Hpl 6	0.27%	A23203G	Syn		
Hpl 7	0.25%	T22944C	L461P		
Hpl 8	0.24%	A23223G	E554G		
Hpl 9	0.23%	A23263G	Syn		
Hpl 10	0.23%	T22942C	Syn		
Hpl 11	0.23%	T22917C	L452P		
Hpl 12	0.23%	T23100C	L513P		
Hpl 13	0.23%	A23261G	R567G		
Hpl 14	0.23%	T22937C	S459P		
Hpl 15	0.23%	T22888C	Syn		
Hpl 16	0.22%	A23169G	N536S		
Hpl 17	0.22%	A23140G	Syn		
Hpl 18	0.21%	T22884C	L441P		
Hpl 19	0.21%	A22956G	E465G		
Hpl 20	0.21%	T23102C	S514P		
Hpl 21	0.21%	T22882C	Syn		
Hpl 22	0.21%	A23207G	T549A		
Hpl 23	0.21%	A23122G	Syn		
Hpl 24	0.21%	A23251G	Syn		
Hpl 25	0.20%	A23229G	N556S		
Hpl 26	0.20%	T23245C	Syn		
Hpl 27	0.20%	T23104C	Syn		
Hpl 28	0.19%	A22935G	K458R		
Hpl 29	0.15%	T23182C	Syn		

Spike A6

Haplotype number	Frequency	Nucleotide mutations[b]	Amino acid substitutions[c]	Nucleotide deletions[b]	Deleted amino acids[c]
Hpl 0	95.09%	A23403G	D614G		
Hpl 1	0.26%	T23332C, A23403G	Syn, D614G		
Hpl 2	0.26%	A23261G, A23403G	R567G, D614G		

Hpl 3	0.26%	A23265G, A23403G	D568G, D614G		
Hpl 4	0.25%	T23394C, A23403G	L611P, D614G		
Hpl 5	0.23%	A23403G, A23550G	D614G, D663G		
Hpl 6	0.22%	A23403G, A23588G	D614G, T676A		
Hpl 7	0.22%	A23403G, A23566G	D614G, Syn		
Hpl 8	0.22%	T23296C, A23403G	Syn, D614G		
Hpl 9	0.22%	A23403G, A23440G	D614G, Syn		
Hpl 10	0.21%	A23263G, A23403G	Syn, D614G		
Hpl 11	0.21%	A23403G	D614G	23555-23582	P665Δ, I666Δ, G667Δ, A668Δ, G669Δ, I670Δ, C671Δ, A672Δ, S673Δ, Y674Δ
Hpl 12	0.21%	T23272C, A23403G	Syn, D614G		
Hpl 13	0.21%	T23293C, A23403G	Syn, D614G		
Hpl 14	0.20%	T23391C, A23403G	V610A, D614G		
Hpl 15	0.20%	A23366G, A23403G	T602A, D614G		
Hpl 16	0.20%	A23403G, T23479C	D614G, Syn		
Hpl 17	0.20%	A23276G, A23403G	T572A, D614G		
Hpl 18	0.20%	T23289C, A23403G	V576A, D614G		
Hpl 19	0.20%	T23333C, A23403G	S591P, D614G		
Hpl 20	0.20%	T23374C, A23403G	Syn, D614G		
Hpl 21	0.19%	A23403G, A23414G	D614G, T618A		
Hpl 22	0.19%	T23307C, A23403G	L582P, D614G		
Hpl 23	0.15%	A23403G	D614G	23561-23582	G667Δ, A668Δ, G669Δ, I670Δ, C671Δ, A672Δ, S673Δ, Y674Δ

[a]The genomic region covered by amplicons A1 to A6 of the S-coding region is: A1: nucleotides 21,448 to 21,841; A2: nucleotides 21,727 to 21,217; A3: nucleotides 22,111 to 22,515; A4: nucleotides 22,487 to 22,882; A5: nucleotides 22,827 to 23,268; A6: nucleotides 23,259 to 23,645. Residue numbering according to NCBI reference sequence: NC_045512.2

[b]The SARS-CoV-2 genome residue numbering is according to the NCBI reference sequence: NC_045512.2. Those haplotypes which do not present any variation with respect the reference sequence are termed Wild type.

[c]Amino acid residues (single-letter code) are numbered from N- to C- terminus of each protein (ORF1b or S); Syn: synonymous mutation.

[e]Haplotypes with amino acid substitutions or deletions characteristic of a different variant that the consensus variant.

Table S10. Haplotypes detected amplicons A1 to A6 of S – coding region in sample from patient Pt460[a].

Spike A1

Haplotype number	Frequency	Nucleotide mutations[b]	Amino acid substitutions[c]	Nucleotide deletions[b]	Deleted amino acids[c]
Hpl 0	94.89%	Wild type			
Hpl 1	0.36%	T21477C	Syn		
Hpl 2	0.35%	A21779G	T73A		
Hpl 3	0.31%	T21737C	F59L		
Hpl 4	0.29%	T21539C	ORF1b:V2691A		
Hpl 5	0.27%	A21489G	Syn		
Hpl 6	0.27%	T21475C	ORF1b:S2670P		
Hpl 7	0.26%	T21479C	ORF1b:L2671P		
Hpl 8	0.24%	A21560G	Syn		
Hpl 9	0.24%	T21594C	V11A		
Hpl 10	0.22%	T21596C	S12P		
Hpl 11[d]	0.22%			21765-21770	H69Δ, V70Δ
Hpl 12	0.22%	T21657C	F32S		
Hpl 13	0.22%	T21540C	Syn		
Hpl 14	0.21%	A21681G	D40G		
Hpl 15	0.21%	A21685G	Syn		
Hpl 16	0.21%	A21720G	D53G		
Hpl 17[d]	0.20%	C21575T	L5F		
Hpl 18	0.20%	T21733C	Syn		
Hpl 19	0.20%	T21841C	Syn		
Hpl 20	0.20%	A21626G	T22A		
Hpl 21	0.20%	T21566C	F2L		

Spike A2

Haplotype number	Frequency	Nucleotide mutations[b]	Amino acid substitutions[c]	Nucleotide deletions[b]	Deleted amino acids[c]
Hpl 0	92.08%	Wild type			
Hpl 1	0.36%	T21737C	F59L		
Hpl 2	0.31%	A22102G	Syn		
Hpl 3	0.29%	T21773C	S71P		
Hpl 4	0.29%	T21755C	F65L		
Hpl 5	0.29%	T21733C	Syn		
Hpl 6	0.26%	A22105G	Syn		
Hpl 7	0.26%	T21841C	Syn		
Hpl 8	0.26%	A22025G	S155G		
Hpl 9	0.25%	A22095G	D178G		
Hpl 10	0.25%	T21771C	V70A		
Hpl 11	0.24%	A22107G	K182R		
Hpl 12	0.24%	T21797C	F79L		
Hpl 13	0.23%	T21908C	S116P		
Hpl 14[d]	0.23%	T21810C	V83A		
Hpl 15	0.23%	A22029G	E156G		

Hpl 16	0.22%	A22101G	E180G		
Hpl 17	0.22%	A22106G	K182E		
Hpl 18	0.22%	T21854C	S98P		
Hpl 19	0.22%	T21808C	Syn		
Hpl 20	0.22%	A22024G	Syn		
Hpl 21	0.22%	T21760C	Syn		
Hpl 22	0.21%	T21831C	V90A		
Hpl 23	0.21%	T22074C	V171A		
Hpl 24	0.20%	A22108G	Syn		
Hpl 25	0.20%	A21852G	K97R		
Hpl 26	0.20%	T21735C	F58S		
Hpl 27	0.20%	T21990C	V143A		
Hpl 28	0.20%	T22084C	Syn		
Hpl 29	0.20%	A22008G	N149S		
Hpl 30	0.20%	T21747C	V62A		
Hpl 31	0.19%	A21900G	K113R		
Hpl 32	0.19%	T22076C	S172P		
Hpl 33[d]	0.18%			21992-21993	Y144Δ
Hpl 34[d]	0.11%			21765-21770. 21992-21994	H69Δ, V70Δ, Y144Δ
Hpl 35[d]	0.11%			21980-21990	F140Δ, L141Δ, G142Δ, V143Δ

Spike A3

Haplotype number	Frequency	Nucleotide mutations[b]	Amino acid substitutions[c]	Nucleotide deletions[b]	Deleted amino acids[c]
Hpl 0	92.79%	C22227T A22488G	A222V, E309G		
Hpl 1	0.65%	C22227T	A222V		
Hpl 2	0.46%	A22488G	E309G		
Hpl 3	0.33%	C22227T A22457G A22488G	A222V, T299A, E309G		
Hpl 4	0.32%	C22227T A22412G A22488G	A222V, T284A, E309G		
Hpl 5	0.31%	C22227T A22431G A22488G	A222V, D290G, E309G		
Hpl 6	0.31%	C22227T A22443G A22488G	A222V, D294G, E309G		
Hpl 7	0.30%	T22209C C22227T A22488G	L216P, A222V, E309G		
Hpl 8	0.29%	C22227T A22488G A22492G	A222V, E309G, Syn		
Hpl 9	0.27%	C22227T T22291C A22488G	A222V, Syn, E309G		

Hpl 10	0.26%	C22227T<br>T22384C<br>A22488G	A222V,<br>Syn,<br>E309G		
Hpl 11	0.26%	T22219C<br>C22227T<br>A22488G	Syn,<br>A222V,<br>E309G		
Hpl 12	0.25%	C22227T<br>A22488G<br>A22496G	A222V,<br>E309G,<br>I312V		
Hpl 13	0.24%	C22227T<br>T22274C<br>A22488G	A222V,<br>F238L,<br>E309G		
Hpl 14	0.23%	T22207C<br>C22227T<br>A22488G	Syn,<br>A222V,<br>E309G		
Hpl 15	0.23%	C22227T<br>A22488G<br>A22495G	A222V,<br>E309G,<br>Syn		
Hpl 16	0.23%	T22213C<br>C22227T<br>A22488G	Syn,<br>A222V,<br>E309G		
Hpl 17	0.22%	C22227T<br>T22371C<br>A22488G	A222V,<br>L270P,<br>E309G		
Hpl 18	0.22%	C22227T<br>T22478C<br>A22488G	A222V,<br>F306L,<br>E309G		
Hpl 19	0.22%	C22227T<br>A22337G<br>A22488G	A222V,<br>T259A,<br>E309G		
Hpl 20	0.21%	C22227T<br>A22301G<br>A22488G	A222V,<br>S247G,<br>E309G		
Hpl 21	0.20%	C22227T<br>A22411G<br>A22488G	A222V,<br>Syn,<br>E309G		
Hpl 22	0.20%	C22227T<br>A22339G<br>A22488G	A222V,<br>Syn,<br>E309G		
Hpl 23	0.20%	C22227T<br>T22348C<br>A22488G	A222V,<br>Syn,<br>E309G		
Hpl 24	0.20%	C22227T<br>A22461G<br>A22488G	A222V,<br>K300R,<br>E309G		
Hpl 25	0.20%	C22227T<br>A22455G<br>A22488G	A222V,<br>E298G,<br>E309G		
Hpl 26	0.19%	C22227T<br>T22324C<br>A22488G	A222V,<br>Syn,<br>E309G		
Hpl 27	0.19%	C22227T<br>A22481G<br>A22488G	A222V,<br>T307A,<br>E309G		

Spike A4

Haplotype number	Frequency	Nucleotide mutations[b]	Amino acid substitutions[c]	Nucleotide deletions[b]	Deleted amino acids[c]

Hpl 0	90.94%	A22488G<br>A22622C	E309G,<br>N354H		
Hpl 1	3.48%	A22622C	Syn	22469-22488	L303Δ, K304Δ,<br>S305Δ, F306Δ,<br>T307Δ, V308Δ,<br>E309Δ
Hpl 2	1.57%	A22488G<br>A22622C<br>T22690C	E309G,<br>N354H,<br>Syn		
Hpl 3	0.32%	A22488G<br>A22622C<br>A22780G	E309G,<br>N354H,<br>Syn		
Hpl 4	0.31%	A22488G<br>A22622C<br>T22736C	E309G,<br>N354H,<br>F392L		
Hpl 5	0.24%	A22488G<br>A22622C<br>A22810G	E309G,<br>N354H,<br>Syn		
Hpl 6	0.24%	A22488G<br>T22497C<br>A22622C	E309G,<br>I312T,<br>N354H		
Hpl 7	0.22%	A22488G<br>A22622C<br>T22711C	E309G,<br>N354H,<br>Syn		
Hpl 8	0.22%	A22488G<br>A22622C<br>A22852G	E309G,<br>N354H,<br>Syn		
Hpl 9	0.22%	A22488G<br>A22622C<br>A22776G	E309G,<br>N354H,<br>D405G		
Hpl 10	0.22%	A22488G<br>A22622C<br>A22771G	E309G,<br>N354H,<br>Syn		
Hpl 11	0.21%	A22488G<br>A22622C<br>A22812G	E309G,<br>N354H,<br>K417R		
Hpl 12	0.21%	A22488G<br>A22582G<br>A22622C	E309G,<br>Syn,<br>N354H		
Hpl 13	0.21%	A22488G<br>T22574C<br>A22622C	E309G,<br>F338L,<br>N354H		
Hpl 14	0.20%	A22488G<br>A22622C<br>A22705G	E309G,<br>N354H,<br>Syn		
Hpl 15	0.20%	A22488G<br>A22622C<br>A22786G	E309G,<br>N354H,<br>Syn		
Hpl 16	0.20%	A22488G<br>T22514C<br>A22622C	E309G,<br>F318L,<br>N354H		
Hpl 17	0.20%	A22488G<br>A22622C<br>A22753G	E309G,<br>N354H,<br>Syn		
Hpl 18	0.20%	A22488G<br>A22622C<br>A22629G	E309G,<br>N354H,<br>K356R		

Hpl 19	0.19%	A22488G A22622C A22631G	E309G, N354H, R357G		
Hpl 20	0.18%	A22488G A22622C T22746C	E309G, N354H, V395A		

Spike A5

Haplotype number	Frequency	Nucleotide mutations[b]	Amino acid substitutions[c]	Nucleotide deletions[b]	Deleted amino acids[c]
Hpl 0	93.14%	Wild type			
Hpl 1	0.30%	A23265G	D568G		
Hpl 2	0.29%	T22944C	L461P		
Hpl 3	0.29%	A23263G	Syn		
Hpl 4[d]	0.29%	A23063T	N501Y		
Hpl 5	0.25%	T22874C	S438P		
Hpl 6	0.25%	T22888C	Syn		
Hpl 7	0.24%	A23223G	E554G		
Hpl 8	0.24%	T23178C	V539A		
Hpl 9	0.24%	T22942C	Syn		
Hpl 10	0.23%	T23030C	F490L		
Hpl 11	0.23%	T23104C	Syn		
Hpl 12	0.23%	T22951C	Syn		
Hpl 13	0.23%	A22956G	E465G		
Hpl 14	0.22%	A23140G	Syn		
Hpl 15	0.22%	A23169G	N536S		
Hpl 16	0.22%	T22876C	Syn		
Hpl 17	0.22%	A23261G	R567G		
Hpl 18	0.22%	A22960G	Syn		
Hpl 19	0.21%	T22937C	S459P		
Hpl 20	0.21%	T23017C	Syn		
Hpl 21	0.21%	A23203G	Syn		
Hpl 22	0.21%	A23209G	Syn		
Hpl 23	0.21%	A22958G	R466G		
Hpl 24	0.20%	A23122G	Syn		
Hpl 25	0.20%	A23089G	Syn		
Hpl 26	0.20%	T23112C	L517P		
Hpl 27	0.20%	T23050C	Syn		
Hpl 28	0.20%	T23032C	Syn		
Hpl 29	0.19%	A23251G	Syn		
Hpl 30	0.19%	A23250G	Q563R		

Spike A6

Haplotype number	Frequency	Nucleotide mutations[b]	Amino acid substitutions[c]	Nucleotide deletions[b]	Deleted amino acids[c]
Hpl 0	96.46%	A23403G	D614G		
Hpl 1	0.46%	A23403G	D614G	23555-23582	P665Δ, I666Δ, G667Δ, A668Δ, G669Δ, I670Δ,

					C671Δ, A672Δ, S673Δ, Y674Δ
Hpl 2	0.26%	A23263G A23403G	Syn, D614G		
Hpl 3	0.25%	A23403G A23594G	D614G, T678A		
Hpl 4	0.24%	A23261G A23403G	R567G, D614G		
Hpl 5	0.23%	A23366G A23403G	T602A, D614G		
Hpl 6	0.22%	A23403G A23524G	D614G, Syn		
Hpl 7	0.22%	A23403G A23566G	D614G, Syn		
Hpl 8	0.22%	A23403G A23550G	D614G, D663G		
Hpl 9	0.21%	T23346C A23403G	V595A, D614G		
Hpl 10	0.21%	A23403G	D614G	23561-23582	G667Δ, A668Δ, G669Δ, I670Δ, C671Δ, A672Δ, S673Δ, Y674Δ
Hpl 11[d]	0.21%	C23271A A23403G C23604A	A570D, D614G, P681H		
Hpl 12	0.21%	T23391C A23403G	V610A, D614G		
Hpl 13	0.20%	A23403G A23503G	D614G, Syn		
Hpl 14	0.20%	A23403G A23588G	D614G, T676A		
Hpl 15	0.20%	T23394C A23403G	L611P, D614G		

[a]The genomic region covered by amplicons A1 to A6 of the S-coding region is: A1: nucleotides 21,448 to 21,841; A2: nucleotides 21,727 to 21,217; A3: nucleotides 22,111 to 22,515; A4: nucleotides 22,487 to 22,882; A5: nucleotides 22,827 to 23,268; A6: nucleotides 23,259 to 23,645. Residue numbering according to NCBI reference sequence: NC_045512.2

[b]The SARS-CoV-2 genome residue numbering is according to the NCBI reference sequence: NC_045512.2. Those haplotypes which do not present any variation with respect the reference sequence are termed Wild type.

[c]Amino acid residues (single-letter code) are numbered from N- to C- terminus of each protein (ORF1b or S); Syn: synonymous mutation.

[d]Haplotypes with amino acid substitutions or deletions characteristic of a different variant that the consensus variant.

Table S11. Haplotypes detected amplicons A1 to A6 of S – coding region in sample from patient Pt461[a].

Spike A1

Haplotype number	Frequency	Nucleotide mutations[b]	Amino acid substitutions[c]	Nucleotide deletions[b]	Deleted amino acids[c]
Hpl 0	91.41%	Wild type			
Hpl 1	0.50%	C21622A	Syn		
Hpl 2	0.43%	T21737C	F59L		
Hpl 3	0.34%	T21596C	S12P		
Hpl 4	0.31%	T21477C	Syn		
Hpl 5	0.26%	T21601C	Syn		
Hpl 6	0.26%	A21720G	D53G		
Hpl 7	0.26%	A21779G	T73A		
Hpl 8	0.25%	T21479C	ORF1b:L2671P		
Hpl 9	0.25%	T21613C	Syn		
Hpl 10	0.25%	T21475C	ORF1b:S2670P		
Hpl 11	0.25%	T21657C	F32S		
Hpl 12	0.23%	A21489G	Syn		
Hpl 13	0.23%	T21539C	ORF1b:V2691A		
Hpl 14	0.23%	A21626G	T22A		
Hpl 15	0.23%	T21524C	ORF1b:V2686A		
Hpl 16	0.22%	A21450G	Syn		
Hpl 17	0.22%	T21655C	Syn		
Hpl 18	0.22%	A21825G	D88G		
Hpl 19	0.22%	T21722C	Syn		
Hpl 20	0.22%	T21540C	Syn		
Hpl 21	0.22%	T21841C	Syn		
Hpl 22	0.22%	A21685G	Syn		
Hpl 23	0.21%	A21794G	R78G		
Hpl 24	0.21%	T21702C	V47A		
Hpl 25	0.21%	T21755C	F65L		
Hpl 26	0.21%	T21733C	Syn		
Hpl 27	0.20%	T21534C	Syn		
Hpl 28	0.20%	A21713G	T51A		
Hpl 29	0.20%	T21818C	F86L		
Hpl 30	0.20%	T21773C	S71P		
Hpl 31	0.20%	A21620G	T20A		
Hpl 32	0.20%	T21497C	ORF1b:L2677P		
Hpl 33	0.19%	T21570C	V3A		
Hpl 34	0.18%	A21681G	D40G		
Hpl 35	0.18%	T21594C	V11A		
Hpl 36	0.17%	A21659G	T33A		

Spike A2

Haplotype number	Frequency	Nucleotide mutations[b]	Amino acid substitutions[c]	Nucleotide deletions[b]	Deleted amino acids[c]
Hpl 0	87.05%	Wild type			

Hpl 1	0.44%	T21737C	F59L		
Hpl 2[d]	0.36%			21992-21993	Y144Δ
Hpl 3	0.35%	A22095G	D178G		
Hpl 4	0.35%	T21771C	V70A		
Hpl 5	0.33%	A22102G	Syn		
Hpl 6	0.32%	A22108G	Syn		
Hpl 7	0.32%	A22025G	S155G		
Hpl 8	0.31%	T21755C	F65L		
Hpl 9	0.30%	T21773C	S71P		
Hpl 10	0.29%	A22106G	K182E		
Hpl 11	0.29%	T21842C	S94P		
Hpl 12	0.28%	A22029G	E156G		
Hpl 13	0.27%	T21733C	Syn		
Hpl 14	0.27%	A22105G	Syn		
Hpl 15	0.26%	A22107G	K182R		
Hpl 16	0.26%	A21900G	K113R		
Hpl 17	0.26%	T21797C	F79L		
Hpl 18	0.24%	A22101G	E180G		
Hpl 19	0.24%	T21735C	F58S		
Hpl 20	0.24%	T21841C	Syn		
Hpl 21	0.24%	A21852G	K97R		
Hpl 22	0.23%	T22084C	Syn		
Hpl 23	0.23%	A22110G	Q183R		
Hpl 24	0.23%	T21808C	Syn		
Hpl 25	0.22%	A21849G	E96G		
Hpl 26	0.22%	T22074C	V171A		
Hpl 27	0.22%	A22023G	E154G		
Hpl 28	0.22%	T21889C	Syn		
Hpl 29[d]	0.22%	T21810C	V83A		
Hpl 30	0.22%	T21854C	S98P		
Hpl 31	0.22%	T21953C	C131R		
Hpl 32	0.22%	A21902G	T114A		
Hpl 33	0.21%	T21760C	Syn		
Hpl 34	0.21%	T21734C	F58L		
Hpl 35	0.21%	T21955C	Syn		
Hpl 36	0.21%	A21957G	E132G		
Hpl 37	0.20%	A22019G	M153V		
Hpl 38	0.20%	A22008G	N149S		
Hpl 39	0.20%	T21831C	V90A		
Hpl 40	0.20%	T21747C	V62A		
Hpl 41	0.20%	T21951C	V130A		
Hpl 42	0.20%	T21775C	Syn		
Hpl 43	0.20%	T21813C	L84P		
Hpl 44	0.20%	A21949G	Syn		
Hpl 45	0.20%	A22056G	N165S		
Hpl 46	0.19%		L110I[e]	21888-21890	T108Δ

Hpl 47	0.19%	A21779G	T73A		
Hpl 48	0.19%	T22076C	S172P		
Hpl 49	0.18%	T21990C	V143A		
Hpl 50[d]	0.18%	A22034G	R158G		
Hpl 51	0.18%	T21787C	Syn		
Hpl 52	0.18%	T21751C	Syn		
Hpl 53	0.17%	T21739C	Syn		
Hpl 54	0.17%	T21769C	Syn		

Spike A3

Haplotype number	Frequency	Nucleotide mutations[b]	Amino acid substitutions[c]	Nucleotide deletions[b]	Deleted amino acids[c]
Hpl 0	89.36%	Wild type			
Hpl 1	0.59%	C22452T	S297L		
Hpl 2	0.36%	A22492G	Syn		
Hpl 3	0.32%	T22228C	Syn		
Hpl 4	0.32%	T22209C	L216P		
Hpl 5	0.31%	A22443G	D294G		
Hpl 6	0.31%	T22291C	Syn		
Hpl 7	0.29%	A22412G	T284A		
Hpl 8	0.28%	T22514C	Syn		
Hpl 9	0.28%	T22478C	F306L		
Hpl 10	0.27%	A22431G	D290G		
Hpl 11	0.26%	A22457G	T299A		
Hpl 12	0.26%	T22371C	L270P		
Hpl 13	0.25%	T22384C	Syn		
Hpl 14	0.25%	A22455G	E298G		
Hpl 15	0.25%	A22496G	I312V		
Hpl 16	0.25%	T22142C	F194L		
Hpl 17	0.23%	T22219C	Syn		
Hpl 18	0.23%	T22447C	Syn		
Hpl 19	0.23%	A22382G	T274A		
Hpl 20	0.23%	A22245G	D228G		
Hpl 21	0.23%	T22213C	Syn		
Hpl 22	0.23%	A22337G	T259A		
Hpl 23	0.22%	A22411G	Syn		
Hpl 24	0.22%	T22200C	V213A		
Hpl 25	0.22%	A22495G	Syn		
Hpl 26	0.22%	A22461G	K300R		
Hpl 27	0.21%	T22274C	F238L		
Hpl 28	0.21%	T22287C	L242P		
Hpl 29	0.21%	T22348C	Syn		
Hpl 30	0.20%	A22374G	Q271R		
Hpl 31	0.20%	T22282C	Syn		
Hpl 32	0.20%	T22117C	Syn		
Hpl 33	0.20%	T22449C	L296P		
Hpl 34	0.20%	A22420G	Syn		

Hpl 35	0.20%	T22207C	Syn		
Hpl 36	0.19%	T22451C	S297P		
Hpl 37	0.19%	A22148G	N196D		
Hpl 38	0.19%	A22375G	Syn		
Hpl 39	0.19%	T22386C	F275S		
Hpl 40	0.19%	T22321C	Syn		
Hpl 41	0.19%	T22324C	Syn		
Hpl 42	0.18%	T22435C	Syn		
Hpl 43	0.18%	T22322C	S254P		
Hpl 44	0.18%	A22422G	D287G		

Spike A4

Haplotype number	Frequency	Nucleotide mutations[b]	Amino acid substitutions[c]	Nucleotide deletions[b]	Deleted amino acids[c]
Hpl 0	90.98%	Wild type			
Hpl 1	1.13%	T22690C	Syn		
Hpl 2	0.41%	T22736C	F392L		
Hpl 3	0.38%	A22780G	Syn		
Hpl 4	0.31%	T22514C	F318L		
Hpl 5	0.30%	A22771G	Syn		
Hpl 6	0.27%	T22574C	F338L		
Hpl 7	0.27%	A22852G	Syn		
Hpl 8	0.26%	A22629G	K356R		
Hpl 9	0.26%	T22501C	Syn		
Hpl 10	0.25%	A22776G	D405G		
Hpl 11	0.25%	A22810G	Syn		
Hpl 12	0.24%	T22709C	S383P		
Hpl 13	0.24%	A22623G	N354S		
Hpl 14	0.24%	A22805G	T415A		
Hpl 15	0.24%	T22568C	C336R		
Hpl 16	0.24%	T22711C	Syn		
Hpl 17	0.23%	A22812G	K417R		
Hpl 18	0.23%	A22786G	Syn		
Hpl 19	0.23%	A22492G	Syn		
Hpl 20	0.22%	T22795C	Syn		
Hpl 21	0.22%	G22487C	E309Q		
Hpl 22	0.22%	A22705G	Syn		
Hpl 23	0.21%	A22625G	R355G		
Hpl 24	0.21%	A22631G	R357G		
Hpl 25	0.21%	T22497C	I312T		
Hpl 26	0.21%	T22521C	V320A		
Hpl 27	0.21%	T22579C	Syn		
Hpl 28	0.20%	A22633G	Syn		
Hpl 29	0.20%	A22582G	Syn		
Hpl 30	0.19%	A22798G	Syn		
Hpl 31	0.19%	T22507C	Syn		
Hpl 32	0.18%	T22573C	Syn		

Hpl 33	0.18%	T22548C	F329S		
Hpl 34	0.18%	T22750C	Syn		

Spike A5

Haplotype number	Frequency	Nucleotide mutations[b]	Amino acid substitutions[c]	Nucleotide deletions[b]	Deleted amino acids[c]
Hpl 0	86.32%	T23042C	S494P		
Hpl 1	0.35%	T23042C, A23265G	S494P, D568G		
Hpl 2	0.30%	T23042C, T23042C	F490L, S494P		
Hpl 3	0.30%	T23042C, T23042C	S438P, S494P		
Hpl 4	0.29%	T23042C, A23263G	S494P, Syn		
Hpl 5	0.28%	T23042C, T23042C	Syn, S494P		
Hpl 6	0.28%	T23042C, T23042C	V445A, S494P		
Hpl 7	0.27%	T23042C, T23042C	D467G, S494P		
Hpl 8	0.26%	T23042C, A23122G	S494P, Syn		
Hpl 9	0.26%	T23042C, T23042C	Syn, S494P		
Hpl 10	0.26%	T23042C, A23140G	S494P, Syn		
Hpl 11	0.26%	T23042C, T23042C	S459P, S494P		
Hpl 12	0.26%	T23042C, T23042C	Syn, S494P		
Hpl 13	0.26%	T23042C, A23207G	S494P, T549A		
Hpl 14	0.25%	T23042C, A23223G	S494P, E554G		
Hpl 15	0.25%	T23042C, A23261G	S494P, R567G		
Hpl 16	0.25%	T23042C, A23169G	S494P, N536S		
Hpl 17	0.25%	T23042C, T23042C	R466G, S494P		
Hpl 18	0.24%	T23042C, T23150C	S494P, S530P		
Hpl 19	0.24%	T23042C, T23042C	L461P, S494P		
Hpl 20	0.24%	T23042C, A23129G	S494P, T523A		
Hpl 21	0.23%	T23042C, T23100C	S494P, L513P		
Hpl 22	0.23%	T23042C, T23042C	Syn, S494P		
Hpl 23	0.23%	T23042C, T23042C	Syn, S494P		
Hpl 24	0.23%	T23042C, T23225C	S494P, S555P		
Hpl 25	0.22%	T23042C, A23203G	S494P, Syn		

Hpl 26	0.22%	T23042C, T23042C	S443P, S494P
Hpl 27	0.22%	T23042C, T23245C	S494P, Syn
Hpl 28	0.22%	T23042C, T23214C	S494P, V551A
Hpl 29	0.22%	T23042C, T23042C	Syn, S494P
Hpl 30	0.22%	T23042C, T23042C	Syn, S494P
Hpl 31	0.21%	T23042C, A23209G	S494P, Syn
Hpl 32	0.21%	T23042C, T23042C	Syn, S494P
Hpl 33	0.21%	T23042C, T23042C	Syn, S494P
Hpl 34	0.21%	T23042C, T23042C	K458R, S494P
Hpl 35	0.21%	T23042C, A23148G	S494P, K529R
Hpl 36	0.21%	T23042C, T23178C	S494P, V539A
Hpl 37	0.20%	T23042C, A23087G	S494P, R509G
Hpl 38	0.20%	T23042C, T23189C	S494P, F543L
Hpl 39	0.20%	T23042C, A23251G	S494P, Syn
Hpl 40	0.20%	T23042C, T23104C	S494P, Syn
Hpl 41	0.19%	T23042C, T23042C	Syn, S494P
Hpl 42	0.19%	T23042C, T23215C	S494P, Syn
Hpl 43	0.19%	T23042C, T23182C	S494P, Syn
Hpl 44	0.19%	T23042C, T23042C	Syn, S494P
Hpl 45	0.19%	T23042C, T23042C	L452P, S494P
Hpl 46	0.19%	T23042C, T23042C	Syn, S494P
Hpl 47	0.19%	T23042C, T23197C	S494P, Syn
Hpl 48	0.19%	T23042C, A23089G	S494P, Syn
Hpl 49	0.19%	T23042C, T23112C	S494P, L517P
Hpl 50	0.19%	T23042C, T23102C	S494P, S514P
Hpl 51	0.19%	T23042C, T23042C	Syn, S494P
Hpl 52	0.18%	T23042C, T23042C	I472V, S494P
Hpl 53	0.18%	T23042C, T23042C	E465G, S494P
Hpl 54	0.18%	T23042C, T23042C	I472T, S494P

Hpl 55	0.18%	T23042C,<br>T23042C	Syn,<br>S494P		
Hpl 56	0.18%	T23042C,<br>A23229G	S494P,<br>N556S		
Hpl 57	0.18%	T23042C,<br>A23201G	S494P,<br>T547A		
Hpl 58	0.18%	T23042C,<br>T23042C	Syn,<br>S494P		
Hpl 59	0.18%	T23042C,<br>T23042C	Syn,<br>S494P		
Hpl 60	0.17%	T23042C,<br>T23051C	S494P,<br>F497L		
Hpl 61	0.17%	T23042C,<br>T23042C	Syn,<br>S494P		
Hpl 62	0.17%	T23042C,<br>T23042C	V483A,<br>S494P		

Spike A6

Haplotype number	Frequency	Nucleotide mutations[b]	Amino acid substitutions[c]	Nucleotide deletions[b]	Deleted amino acids[c]
Hpl 0[d]	91.05%	A23403G,<br>C23604A	D614G,<br>P681H		
Hpl 1[d]	0.31%	A23263G,<br>A23403G,<br>C23604A	Syn,<br>D614G,<br>P681H		
Hpl 2[d]	0.31%	A23261G,<br>A23403G,<br>C23604A	R567G,<br>D614G,<br>P681H		
Hpl 3[d]	0.29%	A23403G,<br>A23588G,<br>C23604A	D614G,<br>T676A,<br>P681H		
Hpl 4[d]	0.27%	A23265G,<br>A23403G,<br>C23604A	D568G,<br>D614G,<br>P681H		
Hpl 5[d]	0.26%	T23332C,<br>A23403G,<br>C23604A	Syn,<br>D614G,<br>P681H		
Hpl 6[d]	0.26%	T23289C,<br>A23403G,<br>C23604A	V576A,<br>D614G,<br>P681H		
Hpl 7[d]	0.25%	A23366G,<br>A23403G,<br>C23604A	T602A,<br>D614G,<br>P681H		
Hpl 8[d]	0.25%	A23403G,<br>A23550G,<br>C23604A	D614G,<br>D663G,<br>P681H		
Hpl 9[d]	0.25%	A23403G,<br>T23452C,<br>C23604A	D614G,<br>Syn,<br>P681H		
Hpl 10[d]	0.24%	A23403G,<br>T23500A,<br>C23604A	D614G,<br>Syn,<br>P681H		
Hpl 11[d]	0.24%	A23403G,<br>T23529C,<br>C23604A	D614G,<br>V656A,<br>P681H		
Hpl 12[d]	0.23%	T23394C,<br>A23403G,<br>C23604A	L611P,<br>D614G,<br>P681H		

Hpl 13[d]	0.23%	A23403G, A23594G, C23604A	D614G, T678A, P681H
Hpl 14[d]	0.22%	A23403G, A23419G, C23604A	D614G, Syn, P681H
Hpl 15[d]	0.22%	T23296C, A23403G, C23604A	Syn, D614G, P681H
Hpl 16[d]	0.22%	T23346C, A23403G, C23604A	V595A, D614G, P681H
Hpl 17 [d]	0.22%	A23403G, T23600C, C23604A	D614G, S680P, P681H
Hpl 18[d]	0.22%	T23344C, A23403G, C23604A	Syn, D614G, P681H
Hpl 19[d]	0.22%	T23391C, A23403G, C23604A	V610A, D614G, P681H
Hpl 20[d]	0.22%	T23335C, A23403G, C23604A	Syn, D614G, P681H
Hpl 21[d]	0.22%	A23403G, A23524G, C23604A	D614G, Syn, P681H
Hpl 22[d]	0.21%	A23403G, T23434C, C23604A	D614G, Syn, P681H
Hpl 23[d]	0.21%	A23403G, A23414G, C23604A	D614G, T618A, P681H
Hpl 24[d]	0.21%	A23403G, A23566G, C23604A	D614G, Syn, P681H
Hpl 25[d]	0.21%	A23403G, T23487C, C23604A	D614G, V642A, P681H
Hpl 26[d]	0.20%	A23310G, A23403G, C23604A	E583G, D614G, P681H
Hpl 27[d]	0.20%	A23403G, A23503G, C23604A	D614G, Syn, P681H
Hpl 28[d]	0.20%	T23352C, A23403G, C23604A	V597A, D614G, P681H
Hpl 29[d]	0.20%	A23403G, C23604A, A23614G	D614G, P681H, Syn
Hpl 30[d]	0.20%	T23350C, A23403G, C23604A	Syn, D614G, P681H
Hpl 31[d]	0.20%	T23392C, A23403G, C23604A	Syn, D614G, P681H

Hpl 32[d]	0.20%	A23403G, T23421C, C23604A	D614G, V620A, P681H
Hpl 33[d]	0.20%	T23333C, A23403G, C23604A	S591P, D614G, P681H
Hpl 34[d]	0.20%	A23403G, T23406C, C23604A	D614G, V615A, P681H
Hpl 35[d]	0.19%	A23326G, A23403G, C23604A	Syn, D614G, P681H
Hpl 36[d]	0.19%	A23403G, A23476G, C23604A	D614G, Syn, P681H
Hpl 37[d]	0.19%	T23287C, A23403G, C23604A	Syn, D614G, P681H
Hpl 38[d]	0.19%	A23403G, C23604A, A23636G	D614G, P681H, I692V
Hpl 39[d]	0.19%	A23403G, T23433C, C23604A	D614G, I624T, P681H
Hpl 40[d]	0.19%	A23403G, T23537C, C23604A	D614G, S659P, P681H

[a]The genomic region covered by amplicons A1 to A6 of the S-coding region is: A1: nucleotides 21,448 to 21,841; A2: nucleotides 21,727 to 21,217; A3: nucleotides 22,111 to 22,515; A4: nucleotides 22,487 to 22,882; A5: nucleotides 22,827 to 23,268; A6: nucleotides 23,259 to 23,645. Residue numbering according to NCBI reference sequence: NC_045512.2

[b]The SARS-CoV-2 genome residue numbering is according to the NCBI reference sequence: NC_045512.2. Those haplotypes which do not present any variation respect the reference sequence are called Wild type.

[c]Amino acid residues (single-letter code) are numbered from N- to C- terminus of each protein (ORF1b or S); Syn: synonymous mutation.

[d]Haplotypes with amino acid substitutions or deletions characteristic of a different variant that the consensus variant.

[e]Amino acid substitution L110I is caused by deletion 21,888-21,890.

Table S12. Haplotypes detected amplicons A1 to A6 of S – coding region in sample from patient Pt462[a].

Spike A1					
**Haplotype number**	**Frequency**	**Nucleotide mutations[b]**	**Amino acid substitutions[c]**	**Nucleotide deletions[b]**	**Deleted amino acids[c]**
Hpl 0	90.04%	Wild type			
Hpl 1	0.42%	T21737C	F59L		
Hpl 2	0.31%	T21475C	ORF1b:S2670P		
Hpl 3	0.30%	T21539C	ORF1b:V2691A		
Hpl 4	0.28%	A21489G	Syn		
Hpl 5	0.28%	A21720G	D53G		
Hpl 6	0.26%	T21529C	ORF1b:S2688P		
Hpl 7	0.25%	A21681G	D40G		
Hpl 8	0.25%	T21477C	Syn		
Hpl 9	0.25%	T21613C	Syn		
Hpl 10	0.25%	A21804G	N81S		
Hpl 11	0.24%	A21779G	T73A		
Hpl 12	0.24%	A21560G	Syn		
Hpl 13	0.23%	T21570C	V3A		
Hpl 14	0.23%	T21755C	F65L		
Hpl 15	0.23%	T21479C	ORF1b:L2671P		
Hpl 16	0.23%	A21511G	ORF1b:N2682D		
Hpl 17	0.22%	A21825G	D88G		
Hpl 18	0.22%	T21596C	S12P		
Hpl 19	0.21%	T21771C	V70A		
Hpl 20	0.21%	T21841C	Syn		
Hpl 21	0.21%	T21655C	Syn		
Hpl 22	0.21%	T21628C	Syn		
Hpl 23	0.21%	T21739C	Syn		
Hpl 24	0.21%	T21540C	Syn		
Hpl 25	0.20%	A21694G	Syn		
Hpl 26	0.20%	T21542C	ORF1b:L2692P		
Hpl 27	0.20%	T21773C	S71P		
Hpl 28	0.20%	A21620G	T20A		
Hpl 29	0.20%	T21702C	V47A		
Hpl 30	0.20%	A21794G	R78G		
Hpl 31	0.20%	T21594C	V11A		
Hpl 32	0.19%	T21453C	Syn		
Hpl 33	0.19%	A21743G	N61D		
Hpl 34	0.19%	T21740C	S60P		
Hpl 35	0.19%	T21576C	L5P		
Hpl 36	0.19%	T21482C	ORF1b:L2672P		
Hpl 37	0.19%	A21625G	Syn		
Hpl 38	0.19%	T21733C	Syn		
Hpl 39	0.19%	T21657C	F32S		
Hpl 40	0.19%	T21572C	F4L		
Hpl 41	0.19%	A21764G	I68V		
Hpl 42	0.18%	T21522C	Syn		

Hpl 43	0.18%	A21685G	Syn
Hpl 44	0.18%	T21688C	Syn
Hpl 45	0.16%	T21497C	ORF1b:L2677P

Spike A2

Haplotype number	Frequency	Nucleotide mutations[b]	Amino acid substitutions[c]	Nucleotide deletions[b]	Deleted amino acids[c]
Hpl 0	85.53%	Wild type			
Hpl 1	0.47%	T21737C	F59L		
Hpl 2	0.33%	A22102G	Syn		
Hpl 3	0.32%	G21898A	Syn		
Hpl 4[d]	0.30%			21992-21993	Y144Δ
Hpl 5	0.30%	T21773C	S71P		
Hpl 6	0.29%	T21831C	V90A		
Hpl 7	0.27%	A21768G	H69R		
Hpl 8	0.26%	T22084C	Syn		
Hpl 9	0.26%	T21739C	Syn		
Hpl 10	0.25%	T21771C	V70A		
Hpl 11[d]	0.25%			21980-21990	F140Δ, L141Δ, G142Δ, V143Δ
Hpl 12	0.24%	T21733C	Syn		
Hpl 13	0.24%	T21883C	Syn		
Hpl 14	0.23%	A22108G	Syn		
Hpl 15	0.23%	T21976C	Syn		
Hpl 16	0.23%	A22036G	Syn		
Hpl 17	0.23%	T21735C	F58S		
Hpl 18	0.23%	A21900G	K113R		
Hpl 19	0.23%	T21775C	Syn		
Hpl 20	0.23%	T22065C	F168S		
Hpl 21	0.22%	T21841C	Syn		
Hpl 22	0.22%	T21755C	F65L		
Hpl 23	0.22%	A21851G	K97E		
Hpl 24	0.22%	A22107G	K182R		
Hpl 25	0.22%	A21868G	Syn		
Hpl 26	0.22%	A22019G	M153V		
Hpl 27	0.22%	A22029G	E156G		
Hpl 28	0.22%	A22025G	S155G		
Hpl 29	0.21%	T21854C	S98P		
Hpl 30	0.21%	A22101G	E180G		
Hpl 31	0.21%	A21779G	T73A		
Hpl 32	0.21%	T22048C	Syn		
Hpl 33	0.21%	T21889C	Syn		
Hpl 34	0.21%	T21747C	V62A		
Hpl 35	0.21%	A21957G	E132G		
Hpl 36	0.21%	T21842C	S94P		
Hpl 37	0.21%	T22072C	Syn		
Hpl 38	0.20%	T21921C	V120A		
Hpl 39	0.20%	T21951C	V130A		

Hpl 40	0.20%	A21852G	K97R		
Hpl 41	0.20%	A22013G	S151G		
Hpl 42	0.20%	T21740C	S60P		
Hpl 43	0.20%	T22085C	F175L		
Hpl 44	0.20%	A22095G	D178G		
Hpl 45[d]	0.19%			21983-21991	L141Δ, G142Δ, V143Δ
Hpl 46	0.19%	T21769C	Syn		
Hpl 47	0.19%	T21826C	Syn		
Hpl 48	0.19%	T22099C	Syn		
Hpl 49	0.19%	T22043C	S161P		
Hpl 50	0.19%	A22106G	K182E		
Hpl 51	0.19%	T21797C	F79L		
Hpl 52	0.19%	A22061G	T167A		
Hpl 53	0.19%	A21902G	T114A		
Hpl 54	0.19%	A22105G	Syn		
Hpl 55	0.19%	T21760C	Syn		
Hpl 56	0.19%	T21835C	Syn		
Hpl 57	0.19%	T21829C	Syn		
Hpl 58	0.19%	T22074C	V171A		
Hpl 59	0.19%	T22038C	V159A		
Hpl 60[d]	0.19%	A22034G	R158G		
Hpl 61	0.18%	A22110G	Q183R		
Hpl 62[d]	0.18%	T21810C	V83A		
Hpl 63	0.18%	T22032C	F157S		
Hpl 64	0.18%	A22046G	S162G		
Hpl 65	0.18%	A22024G	Syn		
Hpl 66	0.16%			21887-21891	T109Δ, L110Δ

Spike A3

Haplotype number	Frequency	Nucleotide mutations[b]	Amino acid substitutions[c]	Nucleotide deletions[b]	Deleted amino acids[c]
Hpl 0	90.76%	C22227T	A222V		
Hpl 1	0.35%	C22227T, T22291C	A222V, Syn		
Hpl 2	0.32%	C22227T, A22492G	A222V, Syn		
Hpl 3	0.31%	T22209C, C22227T	L216P, A222V		
Hpl 4	0.29%	C22227T, A22443G	A222V, D294G		
Hpl 5	0.27%	C22227T, A22412G	A222V, T284A		
Hpl 6	0.27%	C22227T, A22457G	A222V, T299A		
Hpl 7	0.26%	C22227T, T22514C	A222V, Syn		
Hpl 8	0.26%	C22227T, A22496G	A222V, I312V		
Hpl 9	0.26%	C22227T, A22488G	A222V, E309G		

Hpl 10	0.25%	T22219C, C22227T	Syn, A222V
Hpl 11	0.25%	C22227T, T22483C	A222V, Syn
Hpl 12	0.24%	C22227T, A22375G	A222V, Syn
Hpl 13	0.23%	T22142C, C22227T	F194L, A222V
Hpl 14	0.23%	C22227T, A22234G	A222V, Syn
Hpl 15	0.23%	C22227T, A22411G	A222V, Syn
Hpl 16	0.23%	C22227T, T22426C	A222V, Syn
Hpl 17	0.23%	C22227T, A22456G	A222V, Syn
Hpl 18	0.23%	C22227T, T22478C	A222V, F306L
Hpl 19	0.22%	C22227T, T22342C	A222V, Syn
Hpl 20	0.22%	C22227T, A22420G	A222V, Syn
Hpl 21	0.21%	C22227T, A22431G	A222V, D290G
Hpl 22	0.21%	C22227T, T22435C	A222V, Syn
Hpl 23	0.21%	C22227T, T22371C	A222V, L270P
Hpl 24	0.21%	C22227T, A22301G	A222V, S247G
Hpl 25	0.21%	T22207C, C22227T	Syn, A222V
Hpl 26	0.21%	C22227T, T22384C	A222V, Syn
Hpl 27	0.20%	C22227T, T22501C	A222V, Syn
Hpl 28	0.20%	C22227T, T22447C	A222V, Syn
Hpl 29	0.20%	C22227T, T22322C	A222V, S254P
Hpl 30	0.20%	T22221C, C22227T	F220S, A222V
Hpl 31	0.20%	C22227T, T22282C	A222V, Syn
Hpl 32	0.20%	C22227T, T22324C	A222V, Syn
Hpl 33	0.19%	C22227T, A22422G	A222V, D287G
Hpl 34	0.19%	C22227T, A22382G	A222V, T274A
Hpl 35	0.19%	T22137C, C22227T	F192S, A222V
Hpl 36	0.19%	C22227T, T22449C	A222V, L296P
Hpl 37	0.19%	C22227T, T22327C	A222V, Syn
Hpl 38	0.19%	T22117C, C22227T	Syn, A222V

Hpl 39	0.17%	C22227T, T22287C	A222V, L242P		
Hpl 40	0.17%	C22227T	A222V	22292-22296	L244Δ, H245Δ
Hpl 41[d]	0.11%	C22227T	A222V	22283-22291	L241Δ, L242Δ, A243Δ

Spike A4

Haplotype number	Frequency	Nucleotide mutations[b]	Amino acid substitutions[c]	Nucleotide deletions[b]	Deleted amino acids[c]
Hpl 0	78.75%	Wild type			
Hpl 1	11.42%	G22487C	E309Q		
Hpl 2	1.05%	T22690C	Syn		
Hpl 3	0.46%			22586-22588	F342Δ
Hpl 4	0.29%	T22547C	F329L		
Hpl 5	0.29%	A22488G, A22622C	E309G, N354H		
Hpl 6	0.27%	A22780G	Syn		
Hpl 7	0.26%	A22776G	D405G		
Hpl 8	0.26%	C22498T	Syn		
Hpl 9	0.25%	T22736C	F392L		
Hpl 10	0.25%	A22842G	D427G		
Hpl 11	0.23%	A22810G	Syn		
Hpl 12	0.23%	T22846C	Syn		
Hpl 13	0.22%	T22643C	C361R		
Hpl 14	0.21%	A22633G	Syn		
Hpl 15	0.21%	T22709C	S383P		
Hpl 16	0.21%	T22756C	Syn		
Hpl 17	0.20%	A22705G	Syn		
Hpl 18	0.20%	T22514C	F318L		
Hpl 19	0.19%	T22746C	V395A		
Hpl 20	0.19%	A22623G	N354S		
Hpl 21	0.19%	T22497C	I312T		
Hpl 22	0.19%	T22760C	F400L		
Hpl 23	0.19%	A22786G	Syn		
Hpl 24	0.19%	T22684C	Syn		
Hpl 25	0.19%	A22852G	Syn		
Hpl 26	0.19%	T22501C	Syn		
Hpl 27	0.19%	A22812G	K417R		
Hpl 28	0.19%	A22771G	Syn		
Hpl 29	0.18%	A22519G	Syn		
Hpl 30	0.18%	A22803G	Q414R		
Hpl 31	0.18%	A22784G	R408G		
Hpl 32	0.18%	T22548C	F329S		
Hpl 33	0.18%	T22586C	F342L		
Hpl 34	0.17%	A22628G	K356E		
Hpl 35	0.17%	A22845G	D428G		
Hpl 36	0.17%	T22507C	Syn		
Hpl 37	0.17%	A22629G	K356R		
Hpl 38	0.17%	A22582G	Syn		

Hpl 39	0.17%	T22574C	F338L		
Hpl 40	0.16%	A22872G	N437S		
Hpl 41	0.16%	A22753G	Syn		
Hpl 42	0.16%	T22711C	Syn		
Hpl 43	0.16%	A22496G	I312V		
Hpl 44	0.16%	A22728G	D389G		
Hpl 45	0.14%	A22641G	N360S		

Spike A5

Haplotype number	Frequency	Nucleotide mutations[b]	Amino acid substitutions[c]	Nucleotide deletions[b]	Deleted amino acids[c]
Hpl 0	87.07%	Wild type			
Hpl 1	0.34%	T22873C	Syn		
Hpl 2	0.31%	A23265G	D568G		
Hpl 3	0.31%	A23223G	E554G		
Hpl 4	0.30%	T23030C	F490L		
Hpl 5	0.30%	T22874C	S438P		
Hpl 6	0.30%	A23263G	Syn		
Hpl 7	0.28%	T22951C	Syn		
Hpl 8	0.27%	T23245C	Syn		
Hpl 9	0.27%	A22956G	E465G		
Hpl 10	0.27%	T22917C	L452P		
Hpl 11	0.26%	T22888C	Syn		
Hpl 12	0.26%	T22944C	L461P		
Hpl 13	0.25%	A23203G	Syn		
Hpl 14	0.25%	T23215C	Syn		
Hpl 15	0.25%	T23112C	L517P		
Hpl 16	0.24%	A22958G	R466G		
Hpl 17	0.24%	A23169G	N536S		
Hpl 18	0.24%	A23140G	Syn		
Hpl 19	0.24%	T23051C	F497L		
Hpl 20	0.24%	A23251G	Syn		
Hpl 21	0.24%	T22937C	S459P		
Hpl 22	0.24%	T23100C	L513P		
Hpl 23	0.24%	A23207G	T549A		
Hpl 24	0.23%	A23209G	Syn		
Hpl 25	0.23%	A23229G	N556S		
Hpl 26	0.22%	A23014G	Syn		
Hpl 27	0.22%	A22960G	Syn		
Hpl 28	0.22%	T23035C	Syn		
Hpl 29	0.22%	A23201G	T547A		
Hpl 30	0.22%	T23010C	V483A		
Hpl 31	0.22%	A23129G	T523A		
Hpl 32	0.22%	A22935G	K458R		
Hpl 33	0.21%	A22962G	D467G		
Hpl 34	0.21%	T22889C	S443P		
Hpl 35	0.20%	T22876C	Syn		
Hpl 36	0.20%	T22990C	Syn		

Hpl 37	0.20%	T22882C	Syn
Hpl 38	0.20%	A23261G	R567G
Hpl 39	0.20%	A23118G	H519R
Hpl 40	0.20%	T23247C	F562S
Hpl 41	0.20%	T23050C	Syn
Hpl 42	0.20%	A23122G	Syn
Hpl 43	0.20%	T22929C	F456S
Hpl 44	0.19%	T22884C	L441P
Hpl 45	0.19%	T23214C	V551A
Hpl 46	0.19%	T22942C	Syn
Hpl 47	0.19%	T23246C	F562L
Hpl 48	0.19%	A23087G	R509G
Hpl 49	0.18%	A23250G	Q563R
Hpl 50	0.18%	T23189C	F543L
Hpl 51	0.18%	T23032C	Syn
Hpl 52	0.18%	A23168G	N536D
Hpl 53	0.18%	T22999C	Syn
Hpl 54	0.18%	T23176C	Syn
Hpl 55	0.18%	T23178C	V539A
Hpl 56	0.17%	T23068C	Syn
Hpl 57	0.15%	T23182C	Syn

Spike A6

Haplotype number	Frequency	Nucleotide mutations[b]	Amino acid substitutions[c]	Nucleotide deletions[b]	Deleted amino acids[c]
Hpl 0	84.96%	C23277T, A23403G	T572I, D614G		
Hpl 1	1.38%	C23277T, A23403G	T572I, D614G	23555-23582	P665Δ, I666Δ, G667Δ, A668Δ, G669Δ, I670Δ, C671Δ, A672Δ, S673Δ, Y674Δ
Hpl 2	1.12%	A23403G	D614G		
Hpl 3	0.52%	A23263G, C23277T, A23403G	Syn, T572I, D614G		
Hpl 4	0.47%	C23277T, A23403G, A23544G	T572I, D614G, E661G		
Hpl 5	0.45%	C23277T, A23403G	T572I, D614G	23561-23582	G667Δ, A668Δ, G669Δ, I670Δ, C671Δ, A672Δ, S673Δ, Y674Δ
Hpl 6	0.44%	A23265G, C23277T, A23403G	D568G, T572I, D614G		
Hpl 7	0.38%	A23261G, C23277T, A23403G	R567G, T572I, D614G		
Hpl 8	0.28%	C23277T, T23289C, A23403G	T572I, V576A, D614G		

Hpl 9	0.27%	C23277T,<br>A23403G,<br>T23600C	T572I,<br>D614G,<br>S680P
Hpl 10	0.26%	C23277T,<br>T23394C,<br>A23403G	T572I,<br>L611P,<br>D614G
Hpl 11	0.25%	C23277T,<br>A23403G,<br>A23594G	T572I,<br>D614G,<br>T678A
Hpl 12	0.25%	C23277T,<br>A23403G,<br>A23495G	T572I,<br>D614G,<br>T645A
Hpl 13	0.25%	C23277T,<br>T23293C,<br>A23403G	T572I,<br>Syn,<br>D614G
Hpl 14	0.24%	C23277T,<br>T23332C,<br>A23403G	T572I,<br>Syn,<br>D614G
Hpl 15	0.24%	C23277T,<br>A23366G,<br>A23403G	T572I,<br>T602A,<br>D614G
Hpl 16	0.23%	C23277T,<br>A23403G,<br>A23503G	T572I,<br>D614G,<br>Syn
Hpl 17	0.23%	C23277T,<br>A23403G,<br>T23489C	T572I,<br>D614G,<br>F643L
Hpl 18	0.23%	C23277T,<br>A23403G,<br>T23411C	T572I,<br>D614G,<br>C617R
Hpl 19	0.22%	C23277T,<br>T23333C,<br>A23403G	T572I,<br>S591P,<br>D614G
Hpl 20	0.22%	C23277T,<br>A23403G,<br>T23645C	T572I,<br>D614G,<br>Syn
Hpl 21	0.22%	C23277T,<br>A23301G,<br>A23403G	T572I,<br>Q580R,<br>D614G
Hpl 22	0.22%	C23277T,<br>A23403G,<br>T23590C	T572I,<br>D614G,<br>Syn
Hpl 23	0.21%	C23277T,<br>A23403G,<br>T23437C	T572I,<br>D614G,<br>Syn
Hpl 24	0.21%	C23277T,<br>A23403G,<br>A23524G	T572I,<br>D614G,<br>Syn
Hpl 25	0.21%	C23277T,<br>A23303G,<br>A23403G	T572I,<br>T581A,<br>D614G
Hpl 26	0.21%	C23277T,<br>T23346C,<br>A23403G	T572I,<br>V595A,<br>D614G
Hpl 27	0.21%	C23277T,<br>A23403G,<br>T23529C	T572I,<br>D614G,<br>V656A

Hpl 28	0.21%	C23277T, A23403G, A23419G	T572I, D614G, Syn
Hpl 29	0.20%	C23277T, A23403G, A23588G	T572I, D614G, T676A
Hpl 30	0.20%	C23277T, A23403G, T23452C	T572I, D614G, Syn
Hpl 31	0.20%	C23277T, A23403G, T23433C	T572I, D614G, I624T
Hpl 32	0.20%	C23277T, A23403G, A23636G	T572I, D614G, I692V
Hpl 33	0.20%	C23277T, A23403G, T23428C	T572I, D614G, Syn
Hpl 34	0.20%	C23277T, T23374C, A23403G	T572I, Syn, D614G
Hpl 35	0.20%	C23277T, T23344C, A23403G	T572I, Syn, D614G
Hpl 36	0.20%	C23277T, A23403G, T23563C	T572I, D614G, Syn
Hpl 37	0.20%	C23277T, T23391C, A23403G	T572I, V610A, D614G
Hpl 38	0.20%	C23277T, A23403G, T23466C	T572I, D614G, V635A
Hpl 39	0.19%	C23277T, T23296C, A23403G	T572I, Syn, D614G
Hpl 40	0.19%	C23277T, A23403G, T23640C	T572I, D614G, I693T
Hpl 41	0.19%	C23277T, A23295G, A23403G	T572I, D578G, D614G
Hpl 42	0.19%	C23277T, A23310G, A23403G	T572I, E583G, D614G
Hpl 43	0.18%	C23277T, A23403G, A23550G	T572I, D614G, D663G
Hpl 44	0.18%	C23277T, A23403G, T23633C	T572I, D614G, S691P
Hpl 45	0.18%	C23277T, A23403G, T23602C	T572I, D614G, Syn
Hpl 46	0.18%	C23277T, A23403G, T23548C	T572I, D614G, Syn

Hpl 47	0.18%	C23277T, A23403G, A23566G	T572I, D614G, Syn		
Hpl 48	0.18%	C23277T, A23403G, A23476G	T572I, D614G, Syn		
Hpl 49	0.17%	C23277T, A23403G, T23479C	T572I, D614G, Syn		
Hpl 50	0.17%	C23277T, A23312G, A23403G	T572I, I584V, D614G		
Hpl 51	0.17%	C23277T, A23326G, A23403G	T572I, Syn, D614G		
Hpl 52	0.17%	C23277T, A23403G, A23414G	T572I, D614G, T618A		
Hpl 53	0.17%	C23277T, A23403G, T23487C	T572I, D614G, V642A		
Hpl 54	0.17%	C23277T, T23350C, A23403G	T572I, Syn, D614G		
Hpl 55	0.16%	C23277T, A23403G	T572I, D614G	23550-23583	I664Δ, P665Δ, I666Δ, G667Δ, A668Δ, G669Δ, I670Δ, C671Δ, A672Δ, S673Δ, Y674Δ
Hpl 56	0.16%	C23277T, A23403G, A23497G	T572I, D614G, Syn		
Hpl 57	0.11%	C23277T, A23403G	T572I, D614G	23542-23560	E661Δ, C662Δ, D663Δ, I664Δ, P665Δ, I666Δ
Hpl 58	0.11%	C23277T, A23403G	T572I, D614G, Y674N[e]	23571-23582	I670Δ, C671Δ, A672Δ, S673Δ

[a]The genomic region covered by amplicons A1 to A6 of the S-coding region is: A1: nucleotides 21,448 to 21,841; A2: nucleotides 21,727 to 21,217; A3: nucleotides 22,111 to 22,515; A4: nucleotides 22,487 to 22,882; A5: nucleotides 22,827 to 23,268; A6: nucleotides 23,259 to 23,645. Residue numbering according to NCBI reference sequence: NC_045512.2

[b]The SARS-CoV-2 genome residue numbering is according to the NCBI reference sequence: NC_045512.2. Those haplotypes which do not present any variation with respect the reference sequence are termed Wild type.

[c]Amino acid residues (single-letter code) are numbered from N- to C- terminus of each protein (ORF1b or S); Syn: synonymous mutation.

[d]Haplotypes with amino acid substitutions or deletions characteristic of a different variant that the consensus variant.

[e]Amino acid substitution Y674N is caused by deletion 23,571 – 23,582

Table S13. Haplotypes detected amplicons A1 to A6 of S – coding region in sample from patient Pt463[a].

Spike A1

Haplotype number	Frequency	Nucleotide mutations[b]	Amino acid substitutions[c]	Nucleotide deletions[b]	Deleted amino acids[c]
Hpl 0	95.46%	Wild type			
Hpl 1	0.31%	T21737C	F59L		
Hpl 2	0.27%	A21630T	Q23L		
Hpl 3	0.26%	T21477C	Syn		
Hpl 4	0.25%	A21779G	T73A		
Hpl 5	0.24%	A21720G	D53G		
Hpl 6	0.24%	T21540C	Syn		
Hpl 7	0.24%	A21489G	Syn		
Hpl 8	0.23%	T21475C	ORF1b:S2670P		
Hpl 9	0.23%	T21596C	S12P		
Hpl 10	0.23%	T21594C	V11A		
Hpl 11[d]	0.21%	C21575T	L5F		
Hpl 12	0.21%	T21479C	ORF1b:L2671P		
Hpl 13	0.21%	T21841C	Syn		
Hpl 14	0.21%	T21566C	F2L		
Hpl 15	0.21%	T21539C	ORF1b:V2691A		
Hpl 16	0.21%	A21825G	D88G		
Hpl 17	0.20%	T21733C	Syn		
Hpl 18	0.20%	A21685G	Syn		
Hpl 19	0.19%	A21560G	Syn		
Hpl 20	0.19%	T21576C	L5P		

Spike A2

Haplotype number	Frequency	Nucleotide mutations[b]	Amino acid substitutions[c]	Nucleotide deletions[b]	Deleted amino acids[c]
Hpl 0	91.18%	Wild type			
Hpl 1	3.07%	G22113A	G184D		
Hpl 2	0.34%	A22102G	Syn		
Hpl 3	0.30%	T21737C	F59L		
Hpl 4	0.29%	T21773C	S71P		
Hpl 5	0.29%	T21755C	F65L		
Hpl 6	0.28%	T21771C	V70A		
Hpl 7	0.26%	T21733C	Syn		
Hpl 8[d]	0.26%	T21810C	V83A		
Hpl 9	0.24%	A22106G	K182E		
Hpl 10	0.24%	A22025G	S155G		
Hpl 11	0.23%	A22108G	Syn		
Hpl 12	0.23%	T22084C	Syn		
Hpl 13	0.23%	T21842C	S94P		
Hpl 14	0.22%	T21797C	F79L		
Hpl 15	0.22%	A22105G	Syn		
Hpl 16	0.22%	A22029G	E156G		
Hpl 17	0.21%	T21854C	S98P		

Hpl 18	0.21%	A22107G	K182R		
Hpl 19	0.21%	A21868G	Syn		
Hpl 20	0.20%	T21841C	Syn		
Hpl 21	0.20%	A22095G	D178G		
Hpl 22	0.20%	A22101G	E180G		
Hpl 23	0.20%	A22008G	N149S		
Hpl 24	0.19%	T21831C	V90A		
Hpl 25	0.17%			21888-21891	L110Δ
Hpl 26[d]	0.12%			21983-21994	L141Δ, G142Δ, V143Δ, Y144Δ

Spike A3

Haplotype number	Frequency	Nucleotide mutations[b]	Amino acid substitutions[c]	Nucleotide deletions[b]	Deleted amino acids[c]
Hpl 0	91.32%	Wild type			
Hpl 1	2.89%	G22113A	G184D		
Hpl 2	0.34%	T22209C	L216P		
Hpl 3	0.33%	A22412G	T284A		
Hpl 4	0.31%	A22492G	Syn		
Hpl 5	0.29%	A22443G	D294G		
Hpl 6	0.28%	T22213C	Syn		
Hpl 7	0.28%	T22219C	Syn		
Hpl 8	0.27%	A22457G	T299A		
Hpl 9	0.26%	T22228C	Syn		
Hpl 10	0.25%	T22291C	Syn		
Hpl 11	0.24%	T22384C	Syn		
Hpl 12	0.23%	A22496G	I312V		
Hpl 13	0.23%	T22274C	F238L		
Hpl 14	0.23%	A22411G	Syn		
Hpl 15	0.22%	A22431G	D290G		
Hpl 16	0.22%	T22371C	L270P		
Hpl 17	0.21%	A22337G	T259A		
Hpl 18	0.21%	A22461G	K300R		
Hpl 19	0.20%	T22478C	F306L		
Hpl 20	0.20%	A22495G	Syn		
Hpl 21	0.20%	T22321C	Syn		
Hpl 22	0.19%	A22234G	Syn		
Hpl 23	0.19%	A22455G	E298G		
Hpl 24	0.19%	T22447C	Syn		
Hpl 25	0.18%	A22301G	S247G		

Spike A4

Haplotype number	Frequency	Nucleotide mutations[b]	Amino acid substitutions[c]	Nucleotide deletions[b]	Deleted amino acids[c]
Hpl 0	94.88%	Wild type			
Hpl 1	1.50%	T22690C	Syn		
Hpl 2	0.48%	G22487C	E309Q		
Hpl 3	0.36%	A22780G	Syn		
Hpl 4	0.26%	T22736C	F392L		

Hpl 5	0.24%	A22812G	K417R		
Hpl 6	0.24%	A22771G	Syn		
Hpl 7	0.24%	A22705G	Syn		
Hpl 8	0.22%	A22810G	Syn		
Hpl 9	0.22%	A22776G	D405G		
Hpl 10	0.21%			22586-22588	F342Δ
Hpl 11	0.21%	T22507C	Syn		
Hpl 12	0.21%	A22623G	N354S		
Hpl 13	0.21%	A22805G	T415A		
Hpl 14	0.20%	T22584C	V341A		
Hpl 15	0.20%	T22521C	V320A		
Hpl 16	0.10%			22739-22742	T393Δ, N394Δ

Spike A5

Haplotype number	Frequency	Nucleotide mutations[b]	Amino acid substitutions[c]	Nucleotide deletions[b]	Deleted amino acids[c]
Hpl 0[d]	79.35%	G22992A	S477N		
Hpl 1[d]	16.00%	G22992A, C23185T	S477N, Syn		
Hpl 2	0.28%	Wild type			
Hpl 3[d]	0.24%	G22992A, A23265G	S477N, D568G		
Hpl 4[d]	0.22%	G22992A, A23263G	S477N, Syn		
Hpl 5[d]	0.22%	T22951C, G22992A	Syn, S477N		
Hpl 6[d]	0.22%	G22992A, A23223G	S477N, E554G		
Hpl 7[d]	0.20%	G22992A, T23100C	S477N, L513P		
Hpl 8[d]	0.19%	G22992A, A23207G	S477N, T549A		
Hpl 9[d]	0.19%	G22992A, A23140G	S477N, Syn		
Hpl 10[d]	0.18%	G22992A, A23169G	S477N, N536S		
Hpl 11[d]	0.18%	T22874C, G22992A	S438P, S477N		
Hpl 12[d]	0.17%	A22960G, G22992A	Syn, S477N		
Hpl 13[d]	0.17%	G22992A, A23089G	S477N, Syn		
Hpl 14[d]	0.17%	G22992A, T23030C	S477N, F490L		
Hpl 15[d]	0.17%	T22944C, G22992A	L461P, S477N		
Hpl 16[d]	0.17%	G22992A, T23026C	S477N, Syn		
Hpl 17[d]	0.17%	T22942C, G22992A	Syn, S477N		
Hpl 18[d]	0.17%	T22917C, G22992A	L452P, S477N		
Hpl 19[d]	0.17%	G22992A, T23245C	S477N, Syn		

Hpl 20[d]	0.17%	T22888C, G22992A	Syn, S477N		
Hpl 21[d]	0.17%	G22992A, A23209G	S477N, Syn		
Hpl 22[d]	0.17%	G22992A, T23112C	S477N, L517P		
Hpl 23[d]	0.17%	T22876C, G22992A	Syn, S477N		
Hpl 24[d]	0.17%	G22992A, A23122G	S477N, Syn		
Hpl 25[d]	0.16%	G22992A, T23104C	S477N, Syn		
Hpl 26[d]	0.16%	T22937C, G22992A	S459P, S477N		

Spike A6

Haplotype number	Frequency	Nucleotide mutations[b]	Amino acid substitutions[c]	Nucleotide deletions[b]	Deleted amino acids[c]
Hpl 0	96.26%	A23403G	D614G		
Hpl 1	0.72%	A23403G	D614G	23555-23582	P665Δ, I666Δ, G667Δ, A668Δ, G669Δ, I670Δ, C671Δ, A672Δ, S673Δ, Y674Δ
Hpl 2	0.46%	A23403G	D614G	23561-23582	G667Δ, A668Δ, G669Δ, I670Δ, C671Δ, A672Δ, S673Δ, Y674Δ
Hpl 3	0.24%	T23332C, A23403G	Syn, D614G		
Hpl 4	0.23%	T23335C, A23403G	Syn, D614G		
Hpl 5	0.23%	A23265G, A23403G	D568G, D614G		
Hpl 6	0.22%	T23344C, A23403G	Syn, D614G		
Hpl 7	0.22%	T23346C, A23403G	V595A, D614G		
Hpl 8	0.22%	A23403G, A23550G	D614G, D663G		
Hpl 9	0.22%	A23403G, A23503G	D614G, Syn		
Hpl 10	0.21%	A23403G, A23566G	D614G, Syn		
Hpl 11	0.20%	T23394C, A23403G	L611P, D614G		
Hpl 12	0.20%	A23261G, A23403G	R567G, D614G		
Hpl 13	0.15%	A23403G	D614G, Y674N[e]	23571-23582	I670Δ, C671Δ, A672Δ, S673Δ
Hpl 14	0.13%	A23403G	D614G	23570-23571	I670Δ
Hpl 15	0.12%	A23403G	D614G	23555-23570	P665Δ, I666Δ, G667Δ, A668Δ, G669Δ, I670Δ

[a]The genomic region covered by amplicons A1 to A6 of the S-coding region is: A1: nucleotides 21,448 to 21,841; A2: nucleotides 21,727 to 21,217; A3: nucleotides 22,111 to 22,515; A4: nucleotides 22,487 to 22,882; A5: nucleotides 22,827 to 23,268; A6: nucleotides 23,259 to 23,645. Residue numbering according to NCBI reference sequence: NC_045512.2

[b]The SARS-CoV-2 genome residue numbering is according to the NCBI reference sequence: NC_045512.2. Those haplotypes which do not present any variation with respect the reference sequence are termed Wild type.

[c]Amino acid residues (single-letter code) are numbered from N- to C- terminus of each protein (ORF1b or S); Syn: synonymous mutation.

[d]Haplotypes with amino acid substitutions or deletions characteristic of a different variant that the consensus variant.

[e]Substitution Y674N is caused by deletion 23,571-23,582.

Table S14. Haplotypes detected amplicons A1 to A6 of S – coding region in sample from patient Pt500[a].

Spike A1

Haplotype number	Frequency	Nucleotide mutations[b]	Aminoacid substitutions[c]	Nucleotide deletions[b]	Deleted amino acids[c]
Hpl 0[d]	98.19%	C21614T, A21801C	L18F, D80A		
Hpl 1[d]	0.35%	C21575T, C21614T, A21801C	L5F, L18F, D80A		
Hpl 2[d]	0.30%	C21614T, A21779G, A21801C	L18F, T73A, D80A		
Hpl 3[d]	0.27%	T21477C, C21614T, A21801C	Syn, L18F, D80A,		
Hpl 4[d]	0.27%	T21594C, C21614T, A21801C	V11A, L18F, D80A		
Hpl 5[d]	0.26%	A21489G, C21614T, A21801C	Syn, L18F, D80A,		
Hpl 6[d]	0.22%	C21614T, T21737C, A21801C	L18F, F59L, D80A		
Hpl 7[d]	0.16%	C21762T	A67V	21765-21770	H69Δ, V70Δ

Spike A2

Haplotype number	Frequency	Nucleotide mutations[b]	Aminoacid substitutions[c]	Nucleotide deletions[b]	Deleted amino acids[c]
Hpl 0	90.48%	A21801C	D80A		
Hpl 1[d]	0.42%	A21801C	D80A	21992-21993	Y144Δ
Hpl 2	0.41%	A21801C, C22000T	D80A, Syn		
Hpl 3	0.34%	T21737C, A21801C	F59L, D80A		
Hpl 4	0.32%	A21801C, A22102G	D80A, Syn		
Hpl 5	0.30%	T21733C, A21801C	Syn, D80A		
Hpl 6	0.29%	A21801C, A22106G	D80A, K182E		
Hpl 7	0.28%	T21773C, A21801C	S71P, D80A		
Hpl 8	0.27%	T21771C, A21801C	V70A, D80A		
Hpl 9	0.26%	A21801C, A22095G	D80A, D178G		
Hpl 10	0.26%	A21801C, A22107G	D80A, K182R		
Hpl 11	0.26%	A21801C, A22025G	D80A, S155G		
Hpl 12	0.26%	T21755C, A21801C	F65L, D80A		
Hpl 13	0.26%	A21801C,	D80A,		

		T21841C	Syn		
Hpl 14	0.25%	A21801C, A22105G	D80A, Syn		
Hpl 15	0.24%	T21797C, A21801C	F79L, D80A		
Hpl 16	0.24%	A21801C, T22084C	D80A, Syn		
Hpl 17	0.23%	A21801C, A22101G	D80A, E180G		
Hpl 18	0.23%	A21801C, A22029G	D80A, E156G		
Hpl 19[d]	0.23%	A21801C, T21810C	D80A, V83A		
Hpl 20	0.23%	A21801C, A21900G	D80A, K113R		
Hpl 21	0.22%	A21801C, A22008G	D80A, N149S		
Hpl 22	0.22%	A21801C, T21831C	D80A, V90A		
Hpl 23	0.22%	T21747C, A21801C	V62A, D80A		
Hpl 24	0.21%	A21801C, T22076C	D80A, S172P		
Hpl 25	0.21%	A21801C, T21854C	D80A, S98P		
Hpl 26	0.21%	T21735C, A21801C	F58S, D80A		
Hpl 27	0.21%	A21801C, T22074C	D80A, V171A		
Hpl 28	0.20%	T21734C, A21801C	F58L, D80A		
Hpl 29	0.20%	A21801C, A21902G	D80A, T114A		
Hpl 30[d]	0.20%	A21801C, T22031C	D80A, F157L		
Hpl 31	0.20%	A21801C, T21889C	D80A, Syn		
Hpl 32	0.20%	A21801C, A22013G	D80A, S151G		
Hpl 33	0.20%	A21801C, T21808C	D80A, Syn		
Hpl 34	0.20%	A21801C, T21842C	D80A, S94P		
Hpl 35	0.19%	A21801C, A22108G	D80A, Syn		
Hpl 36	0.19%	A21801C, A22019G	D80A, M153V		
Hpl 37	0.19%	A21801C, T21990C	D80A, V143A		
Hpl 38	0.18%	A21801C, T22063C	D80A, Syn		
Hpl 39[d]	0.13%	A21801C	D80A	21983-21994	L141Δ, G142Δ, V143Δ, Y144Δ
Hpl 40[d]	0.12%	C21762T, C21846T	A67V, T95I	21765-21770, 21987-21995	H69Δ, V70Δ, G142Δ, V143Δ, Y144Δ

Spike A3

Haplotype number	Frequency	Nucleotide mutations[b]	Aminoacid substitutions[c]	Nucleotide deletions[b]	Deleted amino acids[c]
Hpl 0	93.14%	A22206G	D215G		
Hpl 1	0.31%	A22206G, A22443G	D215G, D294G		
Hpl 2	0.30%	A22206G, A22492G	D215G, Syn		
Hpl 3	0.30%	A22206G, T22209C	D215G, L216P		
Hpl 4	0.28%	A22206G, T22228C	D215G, Syn		
Hpl 5	0.26%	A22206G, A22457G	D215G, T299A		
Hpl 6	0.26%	A22206G, A22301G	D215G, S247G		
Hpl 7	0.25%	A22206G, A22412G	D215G, T284A		
Hpl 8	0.25%	A22206G, A22431G	D215G, D290G		
Hpl 9	0.24%	A22206G, T22384C	D215G, Syn		
Hpl 10	0.24%	A22206G, A22375G	D215G, Syn		
Hpl 11	0.23%	A22206G, A22411G	D215G, Syn		
Hpl 12	0.23%	A22206G, T22274C	D215G, F238L		
Hpl 13	0.22%	A22206G, A22495G	D215G, Syn		
Hpl 14	0.22%	A22206G, T22447C	D215G, Syn	22283-22291	L241Δ, L242Δ, A243Δ
Hpl 15	0.22%	A22206G, A22496G	D215G, I312V		
Hpl 16	0.21%	A22206G, A22339G	D215G, Syn		
Hpl 17	0.21%	A22206G, T22371C	D215G, L270P		
Hpl 18	0.21%	A22206G, T22449C	D215G, L296P		
Hpl 19	0.21%	T22142C, A22206G	F194L, D215G		
Hpl 20	0.21%	A22206G, T22207C	D215G, Syn		
Hpl 21	0.21%	A22206G, A22337G	D215G, T259A		
Hpl 22	0.21%	A22206G, T22219C	D215G, Syn		
Hpl 23	0.21%	A22206G, T22435C	D215G, Syn		
Hpl 24	0.20%	A22206G, T22213C	D215G, Syn		
Hpl 25	0.20%	A22206G, A22461G	D215G, K300R		
Hpl 26	0.20%	A22206G, A22234G	D215G, Syn		
Hpl 27	0.20%	A22206G, T22348C	D215G, Syn		
Hpl 28	0.20%	A22206G,	D215G,		

		T22324C	Syn		
Hpl 29	0.19%	A22206G, A22374G	D215G, Q271R		
Hpl 30[d,e]	0.16%		L212I[f]	22194-22196	N211Δ

Spike A4

Haplotype number	Frequency	Nucleotide mutations[b]	Aminoacid substitutions[c]	Nucleotide deletions[b]	Deleted amino acids[c]
Hpl 0	92.27%	G22813T	K417N		
Hpl 1	1.86%	T22690C, G22813T	Syn, K417N		
Hpl 2	0.39%	T22736C, G22813T	F392L, K417N		
Hpl 3	0.37%	G22487C, G22813T	E309Q, K417N		
Hpl 4	0.27%	A22771G, G22813T	Syn, K417N,		
Hpl 5	0.27%	A22780G, G22813T	Syn, K417N,		
Hpl 6	0.26%	A22786G, G22813T	Syn, K417N,		
Hpl 7	0.25%	A22629G, G22813T	K356R, K417N		
Hpl 8	0.24%	G22813T, A22852G	K417N, Syn		
Hpl 9	0.23%	T22514C, G22813T	F318L, K417N		
Hpl 10	0.23%	T22709C, G22813T	S383P, K417N		
Hpl 11	0.23%	T22507C, G22813T	Syn, K417N,		
Hpl 12	0.23%	A22753G, G22813T	Syn, K417N,		
Hpl 13	0.22%	A22776G, G22813T	D405G, K417N		
Hpl 14	0.21%	A22705G, G22813T	Syn, K417N		
Hpl 15[d]	0.21%	T22679C, G22813T	S373P, K417N		
Hpl 16	0.21%	A22492G, G22813T	Syn, K417N		
Hpl 17	0.20%	T22501C, G22813T	Syn, K417N		
Hpl 18	0.20%	A22728G, G22813T	D389G, K417N		
Hpl 19	0.20%	T22711C, G22813T	Syn, K417N		
Hpl 20	0.20%	A22623G, G22813T	N354S, K417N		
Hpl 21	0.20%	T22497C, G22813T	I312T, K417N		
Hpl 22	0.20%	A22582G, G22813T	Syn, K417N,		
Hpl 23	0.19%	T22521C, G22813T	V320A, K417N		
Hpl 24	0.19%	T22607C, G22813T	S349P, K417N		

Hpl 25	0.18%	A22810G, G22813T	Syn, K417N,		
Hpl 26[d]	0.16%	G22578A, G22599A, T22673C+C22674T, T22679C, C22686T, G22813T	G339D, R346K, S371L, S373P, S375F, K417N		
Hpl 27	0.13%	G22813T	N343Y[g], K417N	22587-22589	F342Δ

Spike A5

Haplotype number	Frequency	Nucleotide mutations[b]	Aminoacid substitutions[c]	Nucleotide deletions[b]	Deleted amino acids[c]
Hpl 0	92.17%	G23012A, A23063T	E484K, N501Y		
Hpl 1	0.28%	G23012A, A23063T, A23265G	E484K, N501Y, D568G		
Hpl 2	0.28%	G23012A, T23030C, A23063T	E484K, F490L, N501Y		
Hpl 3	0.27%	T22951C, G23012A, A23063T	Syn, E484K, N501Y,		
Hpl 4	0.26%	G23012A, A23063T, A23263G	E484K, N501Y, Syn		
Hpl 5	0.24%	A22958G, G23012A, A23063T	R466G, E484K, N501Y		
Hpl 6	0.24%	G23012A, A23063T, A23203G	E484K, N501Y, Syn		
Hpl 7	0.24%	A22960G, G23012A, A23063T	Syn, E484K, N501Y,		
Hpl 8	0.24%	G23012A, A23063T, A23140G	E484K, N501Y, Syn		
Hpl 9	0.24%	T22937C, G23012A, A23063T	S459P, E484K, N501Y		
Hpl 10	0.23%	T22917C, G23012A, A23063T	L452P, E484K, N501Y		
Hpl 11	0.23%	G23012A, A23063T, A23223G	E484K, N501Y, E554G		
Hpl 12	0.23%	G23012A, T23050C, A23063T	E484K, Syn, N501Y,		
Hpl 13	0.22%	G23012A, A23063T, A23169G	E484K, N501Y, N536S		
Hpl 14[d]	0.22%	T22882G, G22898A, G22992A, C22995A,	N440K, G446S, S477N, T478K,		

		A23013C,<br>A23040G,<br>G23048A,<br>A23055G,<br>A23063T,<br>T23075C,<br>C23202A	E484A,<br>Q493R,<br>G496S,<br>Q498R,<br>N501Y,<br>Y505H,<br>T547K
Hpl 15	0.22%	T22944C,<br>G23012A,<br>A23063T	L461P,<br>E484K,<br>N501Y
Hpl 16	0.21%	T22874C,<br>G23012A,<br>A23063T	S438P,<br>E484K,<br>N501Y
Hpl 17	0.21%	T22999C,<br>G23012A,<br>A23063T	Syn,<br>E484K,<br>N501Y,
Hpl 18	0.21%	G23012A,<br>A23063T,<br>T23178C	E484K,<br>N501Y,<br>V539A
Hpl 19	0.21%	A22935G,<br>G23012A,<br>A23063T	K458R,<br>E484K,<br>N501Y
Hpl 20	0.21%	G23012A,<br>A23063T,<br>A23251G	E484K,<br>N501Y,<br>Syn
Hpl 21	0.21%	T22942C,<br>G23012A,<br>A23063T	Syn,<br>E484K,<br>N501Y,
Hpl 22	0.20%	G23012A,<br>A23063T,<br>A23261G	E484K,<br>N501Y,<br>R567G
Hpl 23	0.20%	G23012A,<br>A23063T,<br>T23214C	E484K,<br>N501Y,<br>V551A
Hpl 24	0.20%	Wild type	
Hpl 25	0.20%	G23012A,<br>A23063T,<br>A23229G	E484K,<br>N501Y,<br>N556S
Hpl 26	0.20%	G23012A,<br>T23026C,<br>A23063T	E484K,<br>Syn,<br>N501Y
Hpl 27	0.20%	G23012A,<br>A23063T,<br>T23245C	E484K,<br>N501Y,<br>Syn
Hpl 28	0.20%	A22956G,<br>G23012A,<br>A23063T	E465G,<br>E484K,<br>N501Y
Hpl 29	0.19%	G23012A,<br>A23063T,<br>A23207G	E484K,<br>N501Y,<br>T549A
Hpl 30	0.19%	T22876C,<br>G23012A,<br>A23063T	Syn,<br>E484K,<br>N501Y,
Hpl 31	0.19%	G23012A,<br>A23063T,<br>A23122G	E484K,<br>N501Y,<br>Syn
Hpl 32	0.19%	G23012A,<br>A23063T,<br>T23215C	E484K,<br>N501Y,<br>Syn

Hpl 33	0.19%	G23012A, A23063T, T23112C	E484K, N501Y, L517P		
Hpl 34	0.19%	G23012A, A23063T, T23104C	E484K, N501Y, Syn		
Hpl 35	0.16%	G23012A, A23063T, T23182C	E484K, N501Y, Syn		
Hpl 36[d]	0.12%	G23012A, A23063T, T23075C	E484K, N501Y, Y505H		
Hpl 37	0.10%	G23012A, A23055G, A23063T	E484K, Q498R, N501Y		

Spike A6

Haplotype number	Frequency	Nucleotide mutations[b]	Aminoacid substitutions[c]	Nucleotide deletions[b]	Deleted amino acids[c]
Hpl 0	92.56%	A23403G	D614G		
Hpl 1	0.38%	A23403G, A23544G	D614G, E661G		
Hpl 2	0.28%	A23265G, A23403G	D568G, D614G		
Hpl 3	0.28%	A23261G, A23403G	R567G, D614G		
Hpl 4	0.28%	T23394C, A23403G	L611P, D614G		
Hpl 5[d]	0.27%	A23403G, C23525T, T23599G, C23604A	D614G, H655Y, N679K, P681H		
Hpl 6	0.26%	A23403G, A23588G	D614G, T676A		
Hpl 7	0.25%	A23403G, A23524G	D614G, Syn		
Hpl 8	0.24%	T23289C, A23403G	V576A, D614G		
Hpl 9	0.24%	A23263G, A23403G	Syn, D614G,		
Hpl 10	0.23%	A23403G, A23566G	D614G, Syn		
Hpl 11	0.23%	A23403G, T23489C	D614G, F643L		
Hpl 12	0.23%	A23403G, A23503G	D614G, Syn		
Hpl 13	0.23%	A23403G, A23550G	D614G, D663G		
Hpl 14	0.22%	A23403G, T23452C	D614G, Syn		
Hpl 15	0.22%	T23391C, A23403G	V610A, D614G		
Hpl 16	0.22%	T23332C, A23403G	Syn, D614G		
Hpl 17	0.22%	T23287C, A23403G	Syn, D614G		
Hpl 18	0.21%	A23366G, A23403G	T602A, D614G		

Hpl 19	0.21%	A23403G, T23479C	D614G, Syn	
Hpl 20	0.21%	T23374C, A23403G	Syn, D614G	
Hpl 21	0.20%	T23335C, A23403G	Syn, D614G	
Hpl 22	0.20%	A23403G, A23414G	D614G, T618A	
Hpl 23	0.20%	A23403G, A23419G	D614G, Syn	
Hpl 24	0.20%	A23403G, T23487C	D614G, V642A	
Hpl 25	0.20%	A23403G, T23633C	D614G, S691P	
Hpl 26	0.20%	T23344C, A23403G	Syn, D614G	
Hpl 27	0.20%	A23403G, A23594G	D614G, T678A	
Hpl 28	0.20%	A23403G, A23440G	D614G, Syn	
Hpl 29	0.19%	A23403G, T23433C	D614G, I624T	
Hpl 30	0.19%	A23403G, A23495G	D614G, T645A	
Hpl 31	0.19%	A23403G, T23470C	D614G, Syn	
Hpl 32	0.19%	A23319G, A23403G	D586G, D614G	
Hpl 33	0.18%	A23403G, T23404C	D614G, Syn	

[a]The genomic region covered by amplicons A1 to A6 of the S-coding region is: A1: nucleotides 21,448 to 21,841; A2: nucleotides 21,727 to 21,217; A3: nucleotides 22,111 to 22,515; A4: nucleotides 22,487 to 22,882; A5: nucleotides 22,827 to 23,268; A6: nucleotides 23,259 to 23,645. Residue numbering according to NCBI reference sequence: NC_045512.2

[b]The SARS-CoV-2 genome residue numbering is according to the NCBI reference sequence: NC_045512.2. Those haplotypes which do not present any variation with respect the reference sequence are termed Wild type.

[c]Amino acid residues (single-letter code) are numbered from N- to C- terminus of each protein (ORF1b or S); Syn: synonymous mutation.

[d]Haplotypes with amino acid substitutions or deletions characteristic of a different variant that the consensus variant.

[e]Hpl 30 includes insertion 22,204:GAGCCAGAA that gives rise to insertion of amino acids 214:EPE.

[f]Substitution L212I is caused by deletion 22,194-22,196.

[g]Substitution N343Y is caused by deletion 22,587-22,589.

Table S15. Haplotypes detected in amplicons A5 and A6 of the S-coding region, following infection of Vero E6 cells with SARS-CoV-2 USA-WA1/2020 in the absence or presence of remdesivir (Rdv) 5 µM and 10 µM [a].

No drug

Spike A5

Haplotype number	Frequency	Nucleotide mutations[b]	Amino acid substitutions[c]	Nucleotide deletions[b]	Deleted amino acids[c]
Hpl 0	98.85%	Wild type			
Hpl 1	0.24%	A23265G	D568G		
Hpl 2	0.23%	T23030C	F490L		
Hpl 3	0.23%	T23104C	Syn		
Hpl 4	0.21%	T22942C	Syn		
Hpl 5	0.14%			23184-23200	N542Δ, F543Δ, N544Δ, G545Δ, L546Δ
Hpl 6	0.11%	T23182C	Syn		

Spike A6

Haplotype number	Frequency	Nucleotide mutations[b]	Amino acid substitutions[c, d]	Nucleotide deletions[b]	Deleted amino acids[c]
Hpl 0	50.96%			23594-23629	T678Δ, N679Δ, S680Δ, P681Δ, R682Δ, R683Δ, A684Δ, R685Δ, S686Δ, V687Δ, A688Δ, S689Δ
Hpl 1	20.72%	C23606T	R682W		
Hpl 2[d]	16.53%	C23525T, C23606T	H655Y, R682W		
Hpl 3	5.54%	G23607T	R682L		
Hpl 4	2.72%			23597-23617	N679Δ, S680Δ, P681Δ, R682Δ, R683Δ, A684Δ, R685Δ
Hpl 5	1.18%			23585-23599	Q675Δ, T676Δ, Q677Δ, T678Δ, N679Δ
Hpl 6	1.15%	T23452A, C23606T	Syn, R682W		
Hpl 7	0.94%	G23616A	R685H		
Hpl 8	0.14%	A23544G	E661G	23594-23629	T678Δ, N679Δ, S680Δ, P681Δ, R682Δ, R683Δ, A684Δ, R685Δ, S686Δ, V687Δ, A688Δ, S689Δ
Hpl 9	0.12%	A23366G	T602A		

5 µM Rdv

Spike A5

Haplotype number	Frequency	Nucleotide mutations[b]	Amino acid substitutions[c, d]	Nucleotide deletions[b]	Deleted amino acids[c]
Hpl 0	98.73%	Wild type			
Hpl 1	0.23%	T22944C	L461P		
Hpl 2	0.22%	A23265G	D568G		
Hpl 3	0.21%	T22874C	S438P		
Hpl 4	0.21%	A23140G	Syn		
Hpl 5	0.20%	A22935G	K458R		
Hpl 6	0.20%	T23104C	Syn		

Spike A6

<table>
<tr><th>Haplotype number</th><th>Frequency</th><th>Nucleotide mutations[b]</th><th>Amino acid substitutions[c, d]</th><th>Nucleotide deletions[b]</th><th>Deleted amino acids[c]</th></tr>
<tr><td>Hpl 0</td><td>90.59%</td><td></td><td></td><td>23594-23629</td><td>T678Δ, N679Δ, S680Δ, P681Δ, R682Δ, R683Δ, A684Δ, R685Δ, S686Δ, V687Δ, A688Δ, S689Δ</td></tr>
<tr><td>Hpl 1</td><td>4.98%</td><td>C23606T</td><td>R682W</td><td></td><td></td></tr>
<tr><td>Hpl 2</td><td>3.16%</td><td>G23607T</td><td>R682L</td><td></td><td></td></tr>
<tr><td>Hpl 3[d]</td><td>0.84%</td><td>C23525T,
C23606T</td><td>H655Y,
R682W</td><td></td><td></td></tr>
<tr><td>Hpl 4</td><td>0.23%</td><td>A23544G</td><td>E661G</td><td rowspan="2">23594-23629</td><td rowspan="2">T678Δ, N679Δ, S680Δ, P681Δ, R682Δ, R683Δ, A684Δ, R685Δ, S686Δ, V687Δ, A688Δ, S689Δ</td></tr>
<tr><td>Hpl 5</td><td>0.21%</td><td>T23394C</td><td>L611P</td></tr>
</table>

10 µM Rdv

Spike A5

Haplotype number	Frequency	Nucleotide mutations[b]	Amino acid substitutions[c, d]	Nucleotide deletions[b]	Deleted amino acids[c]
Hpl 0	94.22%	Wild type			
Hpl 1	0.32%	A23265G	D568G		
Hpl 2	0.29%	T23030C	F490L		
Hpl 3	0.26%	T22874C	S438P		
Hpl 4	0.25%	A23223G	E554G		
Hpl 5	0.24%	A23261G	R567G		
Hpl 6	0.23%	A23140G	Syn		
Hpl 7	0.23%	T22944C	L461P		

Hpl 8	0.23%	T22951C	Syn		
Hpl 9	0.23%	A23263G	Syn		
Hpl 10	0.22%	A22958G	R466G		
Hpl 11	0.22%	T22876C	Syn		
Hpl 12	0.21%	T22942C	Syn		
Hpl 13	0.21%	A23169G	N536S		
Hpl 14	0.21%	T23017C	Syn		
Hpl 15	0.21%	T23050C	Syn		
Hpl 16	0.21%	T22999C	Syn		
Hpl 17	0.21%	T22917C	L452P		
Hpl 18	0.20%	A23207G	T549A		
Hpl 19	0.20%	T23035C	Syn		
Hpl 20	0.20%	T22937C	S459P		
Hpl 21	0.20%	T23225C	S555P		
Hpl 22	0.20%	A22960G	Syn		
Hpl 23	0.19%	T23178C	V539A		
Hpl 24	0.19%	A23122G	Syn		
Hpl 25	0.18%	T23112C	L517P		
Hpl 26	0.14%	T23182C	Syn		
Hpl 27[d]	0.11%	A23055G	Q498R		

Spike A6

**Haplotype number**	**Frequency**	**Nucleotide mutations[b]**	**Amino acid substitutions[c, d]**	**Nucleotide deletions[b]**	**Deleted amino acids[c]**
Hpl 0	33.40%			23597-23626	N679Δ, S680Δ, P681Δ, R682Δ, R683Δ, A684Δ, R685Δ, S686Δ, V687Δ, A688Δ
Hpl 1[d]	30.06%	C23525T, C23606T	H655Y, R682W		
Hpl 2	25.66%			23594-23629	T678Δ, N679Δ, S680Δ, P681Δ, R682Δ, R683Δ, A684Δ, R685Δ, S686Δ, V687Δ, A688Δ, S689Δ
Hpl 3	5.79%			23597-23617	N679Δ, S680Δ, P681Δ, R682Δ, R683Δ, A684Δ, R685Δ
Hpl 4[d]	2.19%	C23525T	H655Y		
Hpl 5	1.95%	A23618G	S686G		
Hpl 6	0.95%	G23607T	R682L		

[a]The genomic region covered by amplicons A5 and A6 of the S-coding region is: A5: nucleotides 22,827 to 23,268; A6: nucleotides 23,259 to 23,645. Residue numbering according to NCBI reference sequence: NC_045512.2

[b]The SARS-CoV-2 genome residue numbering is according to the NCBI reference sequence: NC_045512.2. Those haplotypes which do not present any variation with respect the reference sequence are termed Wild type.

[c]Amino acid residues (single-letter code) are numbered from N- to C- terminus of S-protein; Syn: synonymous mutation.

[d]Haplotypes with amino acid substitutions or deletions characteristic of a different variant that the consensus variant.

Table S16. Haplotypes detected in amplicons A5 and A6 of the S-coding region, following infection of Vero E6 cells with SARS-CoV-2 USA-WA1/2020 in the absence or presence of ribavirin (Rib) 100 µM and 150 µM [a].

No drug

Spike A5

Haplotype number	Frequency	Nucleotide mutations[b]	Amino acid substitutions[c]	Nucleotide deletions[b]	Deleted amino acids[c]
Hpl 0	97.41%	Wild type			
Hpl 1	0.24%	A23265G	D568G		
Hpl 2	0.24%	T22951C	Syn		
Hpl 3	0.23%	A23140G	Syn		
Hpl 4	0.23%	A23169G	N536S		
Hpl 5	0.22%	T23100C	L513P		
Hpl 6	0.21%	A23203G	Syn		
Hpl 7	0.21%	A22958G	R466G		
Hpl 8	0.21%	T22876C	Syn		
Hpl 9	0.20%	T22874C	S438P		
Hpl 10	0.20%	A23251G	Syn		
Hpl 11	0.20%	A22935G	K458R		
Hpl 12	0.19%	T23030C	F490L		

Spike A6

Haplotype number	Frequency	Nucleotide mutations[b]	Amino acid substitutions[c]	Nucleotide deletions[b]	Deleted amino acids[c]
Hpl 0	51.46%			23594-23629	T678Δ, N679Δ, S680Δ, P681Δ, R682Δ, R683Δ, A684Δ, R685Δ, S686Δ, V687Δ, A688Δ, S689Δ
Hpl 1[d]	42.27%	C23525T, C23606T	H655Y, R682W		
Hpl 2	2.16%	G23607T	R682L		
Hpl 3	1.91%			23597-23626	N679Δ, S680Δ, P681Δ, R682Δ, R683Δ, A684Δ, R685Δ, S686Δ, V687Δ, A688Δ
Hpl 4	1.00%			23597-23617	N679Δ, S680Δ, P681Δ, R682Δ, R683Δ, A684Δ, R685Δ
Hpl 5	0.45%	G23616A	R685H		
Hpl 6	0.37%			23585-23599	Q675Δ, T676Δ, Q677Δ, T678Δ, N679Δ
Hpl 7[d]	0.26%	C23525T	H655Y		

Hpl 8	0.12%	A23566G	Syn	23594-23629	T678Δ, N679Δ, S680Δ, P681Δ, R682Δ, R683Δ, A684Δ, R685Δ, S686Δ, V687Δ, A688Δ, S689Δ

Rib 100 µM

Spike A5

Haplotype number	Frequency	Nucleotide mutations[b]	Amino acid substitutions[c]	Nucleotide deletions[b]	Deleted amino acids[c]
Hpl 0	87.53%	Wild type			
Hpl 1	2.05%	C23260T	Syn		
Hpl 2	0.42%	C23170A	N536K		
Hpl 3	0.37%	G22899A	G446D		
Hpl 4	0.32%	T23030C	F490L		
Hpl 5	0.29%	A23265G	D568G		
Hpl 6	0.29%	A23140G	Syn		
Hpl 7	0.29%	T22942C	Syn		
Hpl 8	0.27%	A22958G	R466G		
Hpl 9	0.26%	A23209G	Syn		
Hpl 10	0.25%	T22874C	S438P		
Hpl 11	0.25%	A23263G	Syn		
Hpl 12	0.25%	A23229G	N556S		
Hpl 13	0.24%	T22944C	L461P		
Hpl 14	0.24%	A23203G	Syn		
Hpl 15	0.24%	T22951C	Syn		
Hpl 16	0.24%	T23245C	Syn		
Hpl 17	0.23%	A23207G	T549A		
Hpl 18	0.23%	T22917C	L452P		
Hpl 19	0.22%	T23026C	Syn		
Hpl 20	0.22%	T23178C	V539A		
Hpl 21	0.21%	A23089G	Syn		
Hpl 22	0.21%	T23017C	Syn		
Hpl 23	0.21%	T23104C	Syn		
Hpl 24	0.21%	A22960G	Syn		
Hpl 25	0.21%	T23100C	L513P		
Hpl 26	0.21%	G23161A	Syn		
Hpl 27	0.21%	A23169G	N536S		
Hpl 28	0.21%	T23050C	Syn		
Hpl 29	0.20%	T22990C	Syn		
Hpl 30	0.20%	A23125G	Syn		
Hpl 31	0.20%	A22956G	E465G		
Hpl 32	0.20%	T23150C	S530P		
Hpl 33	0.20%	A23122G	Syn		

Hpl 34	0.20%	A22935G	K458R		
Hpl 35	0.19%	T23010C	V483A		
Hpl 36	0.19%	T22876C	Syn		
Hpl 37	0.19%	A22962G	D467G		
Hpl 38	0.19%	T22937C	S459P		
Hpl 39	0.19%	T23214C	V551A		
Hpl 40	0.19%	A23223G	E554G		
Hpl 41	0.19%	T23068C	Syn		
Hpl 42	0.19%	T23070C	V503A		
Hpl 43	0.19%	T22888C	Syn		
Hpl 44	0.18%	A23056G	Syn		
Hpl 45	0.18%	A23251G	Syn		
Hpl 46	0.18%	T23008C	Syn		
Hpl 47	0.17%	A23014G	Syn		

Spike A6

<table>
<tr><th>Haplotype number</th><th>Frequency</th><th>Nucleotide mutations[b]</th><th>Amino acid substitutions[c]</th><th>Nucleotide deletions[b]</th><th>Deleted amino acids[c]</th></tr>
<tr><td>Hpl 0</td><td>53.61%</td><td></td><td></td><td>23594-23629</td><td>T678Δ, N679Δ, S680Δ, P681Δ, R682Δ, R683Δ, A684Δ, R685Δ, S686Δ, V687Δ, A688Δ, S689Δ</td></tr>
<tr><td>Hpl 1[d]</td><td>16.21%</td><td>C23525T, C23606T</td><td>H655Y, R682W</td><td></td><td></td></tr>
<tr><td>Hpl 2</td><td>12.92%</td><td>C23606T</td><td>R682W</td><td></td><td></td></tr>
<tr><td>Hpl 3</td><td>9.03%</td><td>C23260T</td><td>Syn</td><td>23594-23629</td><td>T678Δ, N679Δ, S680Δ, P681Δ, R682Δ, R683Δ, A684Δ, R685Δ, S686Δ, V687Δ, A688Δ, S689Δ</td></tr>
<tr><td>Hpl 4</td><td>1.50%</td><td>G23607T</td><td>R682L</td><td></td><td></td></tr>
<tr><td>Hpl 5</td><td>0.60%</td><td>G23561A, C23606T</td><td>G667S, R682W</td><td></td><td></td></tr>
<tr><td rowspan="2">Hpl 6[e]</td><td rowspan="2">0.34%</td><td rowspan="2"></td><td rowspan="2">Y674N</td><td>23580-23582</td><td>S673Δ</td></tr>
<tr><td>23594-23629</td><td>T678Δ, N679Δ, S680Δ, P681Δ, R682Δ, R683Δ, A684Δ, R685Δ, S686Δ, V687Δ, A688Δ, S689Δ</td></tr>
<tr><td>Hpl 7</td><td>0.26%</td><td>A23544G</td><td>E661G</td><td rowspan="2">23594-23629</td><td rowspan="2">T678Δ, N679Δ, S680Δ, P681Δ, R682Δ, R683Δ, A684Δ, R685Δ, S686Δ, V687Δ, A688Δ, S689Δ</td></tr>
<tr><td>Hpl 8</td><td>0.24%</td><td>A23265G</td><td>D568G</td></tr>
</table>

Hpl 9[d]	0.24%	A23403G	D614G		
Hpl 10[d]	0.22%	G23259A, C23525T, C23606T	Syn, H655Y, R682W		
Hpl 11	0.22%	G23426A	V622I	23594-23629	T678Δ, N679Δ, S680Δ, P681Δ, R682Δ, R683Δ, A684Δ, R685Δ, S686Δ, V687Δ, A688Δ, S689Δ
Hpl 12	0.18%	A23550G	D663G		
Hpl 13	0.17%	A23263G	Syn		
Hpl 14	0.16%	T23394C	L611P		
Hpl 15	0.15%	T23316C	L585P		
Hpl 16	0.15%	T23289C	V576A		
Hpl 17	0.15%	T23352C	V597A		
Hpl 18	0.14%	A23586G	Q675R		
Hpl 19	0.14%	T23466C	V635A		
Hpl 20	0.14%	A23566G	Syn		
Hpl 21	0.13%	T23332C	Syn		
Hpl 22	0.13%	T23344C	Syn		
Hpl 23	0.13%	T23470C	Syn		
Hpl 24	0.13%	A23414G	T618A		
Hpl 25	0.13%	T23428C	Syn		
Hpl 26	0.13%	A23495G	T645A		
Hpl 27	0.13%	T23293C	Syn		
Hpl 28	0.13%	A23419G	Syn		
Hpl 29	0.13%	A23261G	R567G		
Hpl 30	0.13%	T23330C	C590R		
Hpl 31	0.13%	A23524G	Syn		
Hpl 32	0.12%	A23531G	N657D		
Hpl 33	0.12%	T23287C	Syn		
Hpl 34	0.12%	T23346C	V595A		
Hpl 35	0.12%	A23301G	Q580R		
Hpl 36	0.12%	A23503G	Syn		
Hpl 37	0.12%	A23588G	S689Q		
Hpl 38	0.12%	T23391C	V610A		
Hpl 39	0.11%	T23489C	F643L		
Hpl 40[d]	0.11%	C23525T, C23606T	H655Y, R682W	23309-23337	E583Δ, I584Δ, L585Δ, D586Δ, I587Δ, T588Δ, P589Δ, C590Δ, S591Δ, F592Δ
Hpl 41	0.11%	T23385C	V608A	23594-23629	T678Δ, N679Δ, S680Δ, P681Δ, R682Δ, R683Δ, A684Δ, R685Δ, S686Δ, V687Δ, A688Δ, S689Δ
Hpl 42	0.11%	T23584C	Syn		
Hpl 43	0.11%	T23548C	Syn		
Hpl 44	0.11%	T23421C	V620A		
Hpl 45	0.11%	A23326G	Syn		
Hpl 46	0.11%	T23350C	Syn		
Hpl 47	0.10%	T23374C	Syn		

Rib 150 µM

Spike A5

Haplotype number	Frequency	Nucleotide mutations[b]	Amino acid substitutions[c]	Nucleotide deletions[b]	Deleted amino acids[c]
Hpl 0	89.11%	Wild type			
Hpl 1	0.29%	A23263G	Syn		
Hpl 2	0.29%	A23265G	D568G		
Hpl 3	0.28%	A23261G	R567G		
Hpl 4	0.27%	T22944C	L461P		
Hpl 5	0.26%	T23178C	V539A		
Hpl 6	0.25%	T22951C	Syn		
Hpl 7	0.25%	T23215C	Syn		
Hpl 8	0.25%	A22946G	K462E		
Hpl 9	0.25%	A23235G	K558R		
Hpl 10	0.24%	A23169G	N536S		
Hpl 11	0.24%			23157-23160	L533Δ
Hpl 12	0.24%	T23247C	F562S		
Hpl 13	0.24%	T23112C	L517P		
Hpl 14	0.24%	T22937C	S459P		
Hpl 15	0.24%	T23050C	Syn		
Hpl 16	0.23%	T22874C	S438P		
Hpl 17	0.23%	T23245C	Syn		
Hpl 18	0.23%	T22882C	Syn		
Hpl 19	0.23%	T22876C	Syn		
Hpl 20	0.23%	T23030C	F490L		
Hpl 21	0.23%	A23140G	Syn		
Hpl 22	0.22%	A22958G	R466G		
Hpl 23	0.22%	T22889C	S443P		
Hpl 24	0.22%	T23035C	Syn		
Hpl 25	0.21%	A23229G	N556S		
Hpl 26	0.21%	T22888C	Syn		
Hpl 27	0.21%	T23100C	L513P		
Hpl 28	0.21%	A22893G	K444R		
Hpl 29	0.21%	T22942C	Syn		
Hpl 30	0.21%	A23207G	T549A		
Hpl 31	0.21%	A23122G	Syn		
Hpl 32	0.20%	A22960G	Syn		
Hpl 33	0.20%	A22962G	D467G		
Hpl 34	0.20%	A23014G	Syn		
Hpl 35	0.20%	A23089G	Syn		
Hpl 36	0.20%	A23223G	E554G		
Hpl 37	0.20%	T22896C	V445A		
Hpl 38	0.20%	T22917C	L452P		

Hpl 39	0.20%	G22936A	Syn		
Hpl 40	0.20%	T23026C	Syn		
Hpl 41	0.20%	T23008C	Syn		
Hpl 42	0.20%	T23104C	Syn		
Hpl 43	0.20%	T23017C	Syn		
Hpl 44	0.19%	A22956G	E465G		
Hpl 45	0.19%	A23203G	Syn		
Hpl 46	0.19%	T23225C	S555P		
Hpl 47	0.19%	A22935G	K458R		
Hpl 48	0.19%	T23214C	V551A		
Hpl 49	0.15%	T23182C	Syn		

Spike A6

**Haplotype number**	**Frequency**	**Nucleotide mutations**[b]	**Amino acid substitutions**[c]	**Nucleotide deletions**[b]	**Deleted amino acids**[c]
Hpl 0[d]	55.21%	C23525T, C23606T	H655Y, R682W		
Hpl 1	8.64%			23597-23626	N679Δ, S680Δ, P681Δ, R682Δ, R683Δ, A684Δ, R685Δ, S686Δ, V687Δ, A688Δ
Hpl 2	8.15%	A23618G	S686G		
Hpl 3	6.76%			23597-23617	N679Δ, S680Δ, P681Δ, R682Δ, R683Δ, A684Δ, R685Δ
Hpl 4	6.03%			23594-23629	T678Δ, N679Δ, S680Δ, P681Δ, R682Δ, R683Δ, A684Δ, R685Δ, S686Δ, V687Δ, A688Δ, S689Δ
Hpl 5	4.90%	C23606T	R682W		
Hpl 6	4.34%	G23607T	R682L		
Hpl 7[d]	0.70%	G23461A, C23525T, C23606T	W633*, H655Y, R682W		
Hpl 8[d,f]	0.48%	C23525T		23468-23489	Y636Δ, S637Δ, T638Δ, G639Δ, S640Δ, N641Δ, V642Δ, F643Δ
				23598-23606	
Hpl 9	0.26%	G23517A	G652E	23594-23629	T678Δ, N679Δ, S680Δ, P681Δ, R682Δ, R683Δ, A684Δ, R685Δ, S686Δ, V687Δ, A688Δ, S689Δ

Hpl 10[d]	0.24%	A23263G, C23525T, C23606T	Syn, H655Y, R682W
Hpl 11[d]	0.21%	C23525T, C23606T, G23607T	H655Y, R682L, Syn
Hpl 12	0.19%	G23387A, C23606T	A609T, R682W
Hpl 13[d]	0.18%	T23374C, C23525T, C23606T	Syn, H655Y, R682W
Hpl 14[d]	0.17%	T23346C, C23525T, C23606T	V595A, H655Y, R682W
Hpl 15	0.16%	G23340A, C23606T	G593D, R682W
Hpl 16[d]	0.16%	A23261G, C23525T, C23606T	R567G, H655Y, R682W
Hpl 17[d]	0.16%	A23403G, C23525T, C23606T	D614G, H655Y, R682W
Hpl 18[d]	0.15%	A23440G, C23525T, C23606T	Syn, H655Y, R682W
Hpl 19[d]	0.15%	C23525T, A23550G, C23606T	H655Y, D663G, R682W
Hpl 20[d]	0.15%	T23344C, C23525T, C23606T	Syn, H655Y, R682W
Hpl 21[d]	0.14%	C23525T, A23566G, C23606T	H655Y, Syn, R682W
Hpl 22[d]	0.14%	C23525T, A23586G, C23606T	H655Y, Q675R, R682W
Hpl 23[d]	0.14%	T23284C, C23525T, C23606T	Syn, H655Y, R682W
Hpl 24[d]	0.14%	A23414G, C23525T, C23606T	T618A, H655Y, R682W
Hpl 25[d]	0.14%	T23487C, C23525T, C23606T	V642A, H655Y, R682W
Hpl 26[d]	0.14%	T23391C, C23525T, C23606T	V610A, H655Y, R682W
Hpl 27[d]	0.14%	C23525T, A23594G, C23606T	H655Y, T678A, R682W
Hpl 28[d]	0.13%	C23525T, A23588G, C23606T	H655Y, T676A, R682W

Hpl 29[d]	0.13%	T23333C, C23525T, C23606T	S591P, H655Y, R682W
Hpl 30[d]	0.13%	A23366G, C23525T, C23606T	T602A, H655Y, R682W
Hpl 31[d]	0.13%	T23350C, C23525T, C23606T	Syn, H655Y, R682W
Hpl 32[d]	0.12%	T23394C, C23525T, C23606T	L611P, H655Y, R682W
Hpl 33[d]	0.12%	A23265G, C23525T, C23606T	D568G, H655Y, R682W
Hpl 34[d]	0.12%	T23293C, C23525T, C23606T	Syn, H655Y, R682W
Hpl 35[d]	0.12%	A23419G, C23525T, C23606T	Syn, H655Y, R682W
Hpl 36[d]	0.11%	T23289C, C23525T, C23606T	V576A, H655Y, R682W
Hpl 37[d]	0.11%	A23283G, C23525T, C23606T	D574G, H655Y, R682W
Hpl 38[d]	0.11%	C23525T	H655Y
Hpl 39[d]	0.11%	A23495G, C23525T, C23606T	T645A, H655Y, R682W
Hpl 40[d]	0.11%	T23335C, C23525T, C23606T	Syn, H655Y, R682W
Hpl 41[d]	0.11%	A23503G, C23525T, C23606T	Syn, H655Y, R682W

[a]The genomic region covered by amplicons A5 and A6 of the S-coding region is: A5: nucleotides 22,827 to 23,268; A6: nucleotides 23,259 to 23,645. Residue numbering according to NCBI reference sequence: NC_045512.2

[b]The SARS-CoV-2 genome residue numbering is according to the NCBI reference sequence: NC_045512.2. Those haplotypes which do not present any variation with respect the reference sequence are termed Wild type.

[c]Amino acid residues (single-letter code) are numbered from N- to C- terminus of S-protein; Syn: synonymous mutation.

[d]Haplotypes with amino acid substitutions or deletions characteristic of a different variant that the consensus variant.

[e]Amino acid substitution Y674N is caused by deletion 23,580 – 23,582.

[f]The 23,468-23,489 deletion gives rise a stop codon.

Table S17. Haplotypes detected in amplicons A5 and A6 of S-coding region, following infection of Vero E6 cells with SARS-CoV-2 USA-WA1/2020 in the absence or presence of remdesivir (Rdv) 2.5 µM or 5 µM and ribavirin (Rib) 80 µM or 100 µM [a].

No drug

Spike A5

Haplotype number	Frequency	Nucleotide mutations[b]	Amino acid substitutions[c]	Nucleotide deletions[b]	Deleted amino acids[c]
Hpl 0	98.62%	Wild type			
Hpl 1	1.03%	C23057T	P499S		
Hpl 2	0.21%	T23100C	L513P		
Hpl 3	0.13%	T23182C	Syn		

Spike A6

Haplotype number	Frequency	Nucleotide mutations[b]	Amino acid substitutions[c]	Nucleotide deletions[b]	Deleted amino acids[c]
Hpl 0	64.92%			23594-23629	T678Δ, N679Δ, S680Δ, P681Δ, R682Δ, R683Δ, A684Δ, R685Δ, S686Δ, V687Δ, A688Δ, S689Δ
Hpl 1	10.05%	C23606T	R682W		
Hpl 2[d]	10.00%	C23525T, C23606T	H655Y, R682W		
Hpl 3	8.30%	G23607T	R682L		
Hpl 4	2.14%			23597-23617	N679Δ, S680Δ, P681Δ, R682Δ, R683Δ, A684Δ, R685Δ
Hpl 5	1.63%			23585-23599	Q675Δ, T676Δ, Q677Δ, T678Δ, N679Δ
Hpl 6	0.98%	A23618G	S686G		
Hpl 7	0.77%	G23616A	R685H		
Hpl 8[d]	0.40%	T23434C, C23525T, C23606T	Syn, H655Y, R682W		
Hpl 9	0.22%	T23434C	Syn	23594-23629	T678Δ, N679Δ, S680Δ, P681Δ, R682Δ, R683Δ, A684Δ, R685Δ, S686Δ, V687Δ, A688Δ, S689Δ
Hpl 10	0.17%	A23261G	R567G		
Hpl 11	0.16%	A23265G	D568G		
Hpl 12	0.14%	A23263G	Syn		
Hpl 13	0.13%	T23394C	L611P		

Rdv 2.5 µM + Rib 80 µM

Spike A5

Haplotype number	Frequency	Nucleotide mutations[b]	Amino acid substitutions[c]	Nucleotide deletions[b]	Deleted amino acids[c]
Hpl 0	95.62%	Wild type			
Hpl 1	2.69%	A23267C	Syn		

Hpl 2	0.89%	C23057T	P499S		
Hpl 3	0.46%	C22995T	T478I		
Hpl 4[d]	0.21%	T22882G, A22893C, T22917G, T22942A, G22992A, C22995A, A23013C, T23018G, A23055G, A23063T, T23075C	N440K, K444T, L452R, N460K, S477N, T478K, E484A, F486V, Q498R, N501Y, Y505H		
Hpl 5[d]	0.12%	A23055G	Q498R		

Spike A6

Haplotype number	Frequency	Nucleotide mutations[b]	Amino acid substitutions[c]	Nucleotide deletions[b]	Deleted amino acids[c]
Hpl 0	75.80%			23594-23629	T678Δ, N679Δ, S680Δ, P681Δ, R682Δ, R683Δ, A684Δ, R685Δ, S686Δ, V687Δ, A688Δ, S689Δ
Hpl 1[d]	13.66%	C23525T, C23606T	H655Y, R682W		
Hpl 2	4.92%	A23267C	I569L	23594-23629	T678Δ, N679Δ, S680Δ, P681Δ, R682Δ, R683Δ, A684Δ, R685Δ, S686Δ, V687Δ, A688Δ, S689Δ
Hpl 3	4.18%	G23607T	R682L		
Hpl 4[d]	0.21%	A23403G, C23525T, T23599G, C23604A	D614G, H655Y, N679K, P681H		
Hpl 5	0.20%	A23261G	R567G	23594-23629	T678Δ, N679Δ, S680Δ, P681Δ, R682Δ, R683Δ, A684Δ, R685Δ, S686Δ, V687Δ, A688Δ, S689Δ
Hpl 6	0.18%	A23263G	Syn		
Hpl 7	0.18%	A23265G	D568G		
Hpl 8	0.17%	T23489C	F643L		
Hpl 9	0.17%	T23479C	Syn		
Hpl 10	0.17%	A23544G	E661G		
Hpl 11	0.17%	A23550G	D663G		

Rdv 5 µM + Rib 80 µM

Spike A5

Haplotype number	Frequency	Nucleotide mutations[b]	Amino acid substitutions[c]	Nucleotide deletions[b]	Deleted amino acids[c]
Hpl 0	97.39%	Wild type			
Hpl 1	1.00%	C23057T	P499S		
Hpl 2	0.36%	C23127T	A522V		
Hpl 3	0.24%	T23104C	Syn		

Hpl 4	0.21%	A23265G	D568G		
Hpl 5	0.21%	A23263G	Syn		
Hpl 6	0.21%	T22944C	L461P		
Hpl 7	0.20%	T22874C	S438P		
Hpl 8	0.20%	T23050C	Syn		

Spike A6

Haplotype number	Frequency	Nucleotide mutations[b]	Amino acid substitutions[c]	Nucleotide deletions[b]	Deleted amino acids[c]
Hpl 0	59.00%			23594-23629	T678Δ, N679Δ, S680Δ, P681Δ, R682Δ, R683Δ, A684Δ, R685Δ, S686Δ, V687Δ, A688Δ, S689Δ
Hpl 1[d]	22.83%	C23525T, C23606T	H655Y, R682W		
Hpl 2	13.12%	C23606T	R682W		
Hpl 3	3.21%	G23607T	R682L		
Hpl 4	0.62%	G23616A	R685H		
Hpl 5	0.40%			23597-23617	N679Δ, S680Δ, P681Δ, R682Δ, R683Δ, A684Δ, R685Δ
Hpl 6	0.15%	A23265G	D568G	23594-23629	T678Δ, N679Δ, S680Δ, P681Δ, R682Δ, R683Δ, A684Δ, R685Δ, S686Δ, V687Δ, A688Δ, S689Δ
Hpl 7	0.15%	T23394C	L611P		
Hpl 8	0.14%	T23391C	V610A		
Hpl 9	0.14%	A23588G	S689Q		
Hpl 10	0.13%	A23261G	R567G		
Hpl 11	0.12%	A23544G	E661G		

Rdv 2.5 µM + Rib 100 µM

Spike A5

Haplotype number	Frequency	Nucleotide mutations[b]	Amino acid substitutions[c]	Nucleotide deletions[b]	Deleted amino acids[c]
Hpl 0	96.82%	Wild type			
Hpl 1	1.02%	C23057T	P499S		
Hpl 2	0.56%	G22927A	Syn		
Hpl 3	0.23%	T22951C	Syn		
Hpl 4	0.23%	A23263G	Syn		
Hpl 5	0.22%	T22874C	S438P		
Hpl 6	0.22%	A23223G	E554G		
Hpl 7	0.21%	T23246C	F562L		
Hpl 8	0.20%	A23265G	D568G		
Hpl 9	0.15%	T23182C	Syn		
Hpl 10[d]	0.14%	G23012A, A23063T	E484K, N501Y		

Spike A6

Haplotype number	Frequency	Nucleotide mutations[b]	Amino acid substitutions[c]	Nucleotide deletions[b]	Deleted amino acids[c]
Hpl 0	64.88%			23594-23629	T678Δ, N679Δ, S680Δ, P681Δ, R682Δ, R683Δ, A684Δ, R685Δ, S686Δ, V687Δ, A688Δ, S689Δ
Hpl 1[d]	14.62%	C23525T, C23606T	H655Y, R682W		
Hpl 2	10.01%	G23607T	R682L		
Hpl 3	8.18%			23597-23617	N679Δ, S680Δ, P681Δ, R682Δ, R683Δ, A684Δ, R685Δ
Hpl 4	0.36%	A23618G	S686G		
Hpl 5	0.21%	A23261G	R567G	23594-23629	T678Δ, N679Δ, S680Δ, P681Δ, R682Δ, R683Δ, A684Δ, R685Δ, S686Δ, V687Δ, A688Δ, S689Δ
Hpl 6	0.20%	A23588G	S689Q		
Hpl 7	0.20%	A23265G	D568G		
Hpl 8	0.20%			23597-23626	N679Δ, S680Δ, P681Δ, R682Δ, R683Δ, A684Δ, R685Δ, S686Δ, V687Δ, A688Δ
Hpl 9	0.19%	A23263G	Syn	23594-23629	T678Δ, N679Δ, S680Δ, P681Δ, R682Δ, R683Δ, A684Δ, R685Δ, S686Δ, V687Δ, A688Δ, S689Δ
Hpl 10	0.17%	A23544G	E661G		
Hpl 11[d]	0.14%	A23403G, C23604A	D614G, P681H		
Hpl 12	0.14%	A23419G	Syn	23594-23629	T678Δ, N679Δ, S680Δ, P681Δ, R682Δ, R683Δ, A684Δ, R685Δ, S686Δ, V687Δ, A688Δ, S689Δ
Hpl 13	0.13%	A23550G	D663G		
Hpl 14	0.13%	A23276G	T572A		
Hpl 15	0.12%	A23566G	Syn		
Hpl 16	0.12%	T23394C	L611P		

Rdv 5 µM + Rib 100 µM

Spike A5

Haplotype number	Frequency	Nucleotide mutations[b]	Amino acid substitutions[c]	Nucleotide deletions[b]	Deleted amino acids[c]
Hpl 0	94.78%	Wild type			
Hpl 1	1.00%	C23057T	P499S		
Hpl 2	0.46%	G22989A	G476D		
Hpl 3	0.32%	T23178C	V539A		
Hpl 4	0.23%	T22951C	Syn		
Hpl 5	0.23%	A23265G	D568G		

Hpl 6[d]	0.22%	T22882G, G22898A, G22992A, C22995A, A23013C, A23040G, G23048A, A23055G, A23063T, T23075C, C23202A	N440K, G446S, S477N, T478K, E484A, Q493R, G496S, Q498R, N501Y, Y505H, T547K		
Hpl 7	0.22%	T23030C	F490L		
Hpl 8	0.21%	A22956G	E465G		
Hpl 9	0.21%	T23246C	F562L		
Hpl 10	0.21%	T23183C	F541L		
Hpl 11	0.21%	A23263G	Syn		
Hpl 12	0.20%	T22944C	L461P		
Hpl 13	0.20%	T23100C	L513P		
Hpl 14	0.20%	A23014G	Syn		
Hpl 15	0.19%	T22873C	Syn		
Hpl 16	0.19%	T23225C	S555P		
Hpl 17	0.19%	A23169G	N536S		
Hpl 18	0.18%	A22935G	K458R		
Hpl 19[d]	0.11%	A23040G	Q493R		
Hpl 20[d]	0.11%	C23202A	T547K		
Hpl 21[d]	0.11%	A23055G	Q498R		

Spike A6

Haplotype number	Frequency	Nucleotide mutations[b]	Amino acid substitutions[c]	Nucleotide deletions[b]	Deleted amino acids[c]
Hpl 0	59.01%			23594-23629	T678Δ, N679Δ, S680Δ, P681Δ, R682Δ, R683Δ, A684Δ, R685Δ, S686Δ, V687Δ, A688Δ, S689Δ
Hpl 1[d]	10.84%	C23525T, C23606T	H655Y, R682W		
Hpl 2	9.91%	C23606T	R682W		
Hpl 3	6.40%			23597-23617	N679Δ, S680Δ, P681Δ, R682Δ, R683Δ, A684Δ, R685Δ
Hpl 4	3.76%	G23607T	R682L		
Hpl 5	3.46%	A23618G	S686G		
Hpl 6	2.50%	C23575T	Syn	23597-23626	N679Δ, S680Δ, P681Δ, R682Δ, R683Δ, A684Δ, R685Δ, S686Δ, V687Δ, A688Δ
Hpl 7[d]	1.64%	C23525T	H655Y		

Hpl 8	0.37%			23585-23599	Q675Δ, T676Δ, Q677Δ, T678Δ, N679Δ
Hpl 9	0.20%	A23261G	R567G	23594-23629	T678Δ, N679Δ, S680Δ, P681Δ, R682Δ, R683Δ, A684Δ, R685Δ, S686Δ, V687Δ, A688Δ, S689Δ
Hpl 10	0.19%	A23265G	D568G		
Hpl 11	0.19%	A23263G	Syn		
Hpl 12	0.15%	A23544G	E661G		
Hpl 13	0.14%	A23310G	E583G		
Hpl 14	0.14%	T23394C	L611P		
Hpl 15	0.13%	A23550G	D663G		
Hpl 16	0.13%	A23276G	T572A		
Hpl 17	0.13%	A23566G	Syn		
Hpl 18	0.13%	T23537C	S659P		
Hpl 19	0.12%	A23366G	T602A		
Hpl 20	0.12%	T23452C	Syn		
Hpl 21	0.12%	T23509C	Syn		
Hpl 22	0.11%	T23332C	Syn		
Hpl 23	0.11%	A23414G	T618A		

[a]The genomic region covered by amplicons A5 and A6 of the S-coding region is: A5: nucleotides 22,827 to 23,268; A6: nucleotides 23,259 to 23,645. Residue numbering according to NCBI reference sequence: NC_045512.2

[b]The SARS-CoV-2 genome residue numbering is according to the NCBI reference sequence: NC_045512.2. Those haplotypes which do not present any variation with respect the reference sequence are termed Wild type.

[c]Amino acid residues (single-letter code) are numbered from N- to C- terminus of S-protein; Syn: synonymous mutation.

[d]Haplotypes with amino acid substitutions or deletions characteristic of a different variant that the consensus variant.
